# Beeinflussung der psychosozialen Entwicklung von Kindern und Jugendlichen durch das Tragen von Gesichtsmasken im öffentlichen Raum zur Prävention von Infektionskrankheiten: Ein systematischer Review

**DOI:** 10.1007/s00103-021-03443-5

**Published:** 2021-10-25

**Authors:** Alice Freiberg, Katy Horvath, Taurai Monalisa Hahne, Stephanie Drössler, Daniel Kämpf, Anke Spura, Bernhard Buhs, Nadine Reibling, Freia De Bock, Christian Apfelbacher, Andreas Seidler

**Affiliations:** 1 Institut und Poliklinik für Arbeits- und Sozialmedizin, Medizinische Fakultät Carl Gustav Carus, Technische Universität Dresden, Fetscherstraße 74, 01307 Dresden, Deutschland; 2 Klinik für Kinder- und Jugendpsychiatrie, Psychosomatik und Psychotherapie, Helios Park-Klinikum Leipzig, Leipzig, Deutschland; 3 Institut für Sozialmedizin und Gesundheitssystemforschung, Medizinische Fakultät, Otto-von-Guericke Universität Magdeburg, Magdeburg, Deutschland; 4 Bundeszentrale für gesundheitliche Aufklärung, Köln, Deutschland

**Keywords:** Mund-Nasen-Schutz, Maske, Prävention, COVID-19, Infektionserkrankung, Psychosoziale Entwicklung, Kinder und Jugendliche, Mouth-nose protection, Face mask, Prevention, Infectious disease, COVID-19, Psychosocial development, Child and adolescent

## Abstract

**Hintergrund:**

Zur Prävention tröpfchenübertragener Infektionskrankheiten wird das Tragen einer Maske im öffentlichen Raum unter bestimmten Bedingungen empfohlen.

**Ziel der Arbeit:**

Ziel war, über eine sensitive Literatursuche möglichst alle deutsch- und englischsprachigen Forschungsergebnisse aus begutachteten Fachzeitschriftenartikeln zu den Auswirkungen des Masketragens zur Prävention von Infektionen auf die psychosoziale Entwicklung von Kindern und Jugendlichen zusammenzutragen.

**Methoden:**

Es wurde ein systematischer Review unter Berücksichtigung verschiedener Studiendesigns durchgeführt (Suchzeitraum bis einschließlich 12.07.2021). Das Verzerrungsrisiko der Studien wurde mit einem Risk-of-Bias-Verfahren ermittelt. Es fand eine deskriptiv-narrative Ergebnissynthese statt.

**Ergebnisse:**

Es wurden 13 Studien eingeschlossen, wobei das Gesamtverzerrungsrisiko in allen Primärstudien als hoch eingeschätzt wurde. Es gibt Hinweise aus Befragungsstudien, dass die Fähigkeit zum Lesen der Mimik von Kindern/Jugendlichen und/oder ihren Betreuer:innen im (Vor‑)Schulsetting durch das Masketragen als beeinträchtigt erlebt wird, die durch mehrere Experimentalstudien bestätigt wurden. 2 Studien berichteten über psychische Symptome wie Ängste oder Stresserleben sowie Konzentrations- und Lernschwierigkeiten durch das Masketragen während der COVID-19-Pandemie. Eine Studie während der SARS-Pandemie 2002/2003 untersuchte mündliche Prüfungsleistungen in Englisch als Fremdsprache und zeigte keinen Unterschied zwischen den Bedingungen „Maske“ versus „keine Maske“.

**Diskussion:**

Zu den Auswirkungen des Masketragens auf verschiedene Entwicklungsbereiche von Kindern und Jugendlichen lassen sich basierend auf der unzureichenden Studienlage nur wenige Erkenntnisse ableiten. Es fehlen Forschungsdaten zu den Folgen für die Endpunkte psychische Entwicklung, Sprachentwicklung, sozioemotionale Entwicklung, soziales Verhalten, Schulerfolg und Teilhabe. Weitere qualitative Studien und epidemiologische Studien sind unbedingt nötig.

**Zusatzmaterial online:**

Zusätzliche Informationen sind in der Online-Version dieses Artikels (10.1007/s00103-021-03443-5) enthalten.

## Abstract (en)

**Background:**

Wearing face masks in public is recommended under certain circumstances in order to prevent infectious diseases transmitted through droplets.

**Aim:**

The objective was to compile all German and English research results from peer-reviewed journal articles using a sensitive literature search on the effects of mask-wearing for preventing infectious diseases on the psychosocial development of children and adolescents.

**Methods:**

A systematic review was conducted considering different study designs (search period up until 12 July 2021). The risk of bias in the studies was determined using a risk of bias procedure. A descriptive-narrative synthesis of the results was performed.

**Results:**

Thirteen studies were included, and the overall risk of bias was estimated to be high in all primary studies. There are some indications from the included surveys that children, adolescents, and their teachers in (pre)schools perceived facial expression processing as impaired due to mask wearing, which were confirmed by several experimental studies. Two studies reported psychological symptoms like anxiety and stress as well as concentration and learning problems due to wearing a mask during the COVID-19 pandemic. One survey study during the 2002/2003 SARS pandemic examined oral examination performance in English as a foreign language and showed no difference between the “mask” and “no mask” conditions.

**Discussion:**

Only little evidence can be derived on the effects of wearing mouth–nose protection on different developmental areas of children and adolescents based on the small number of studies. There is a lack of research data regarding the following outcomes: psychological development, language development, emotional development, social behavior, school success, and participation. Further qualitative studies and epidemiological studies are required.

## Hintergrund

Das Tragen von Gesichtsmasken zur Prävention einer Infektion mit dem Coronavirus SARS-CoV‑2 wird von nationalen und internationalen public-health-relevanten Organisationen wie der Weltgesundheitsorganisation [1] oder dem Robert Koch-Institut [2] unter bestimmten Umständen empfohlen. Zur Verwendung von Mund-Nasen-Bedeckung oder einer anderen Maske bei Kindern ab dem 6. Lebensjahr wird unter Berücksichtigung bestimmter Aspekte wie Sicherheit und Freiwilligkeit geraten [3, 4]. Die Regelungen unterscheiden sich je nach Bundesland und der jeweiligen Schule oder Kindertagesstätte. Im Folgenden wird zur besseren Lesbarkeit der Begriff „Masken“ verwendet, der „Mund-Nasen-Bedeckungen“ („Alltagsmasken“), „medizinischen Mund-Nasen-Schutz“, „FFP-Masken“ und weitere „Gesichtsmasken“ einschließt. Mit allen soll eine Virusübertragung im Rahmen der COVID-19-Pandemie verhindert werden. Obwohl das Ausmaß der Effektivität noch diskutiert wird – insbesondere in Bezug auf die Nutzung von Masken im Freien –, wird der Einsatz von Masken als Public-Health-Maßnahme empfohlen [5–7].

Der Einsatz von Masken wurde jedoch insbesondere zu Beginn der Pandemie auch kritisch diskutiert: So können bei Erwachsenen körperliche Symptome wie Kopfschmerzen oder kardiovaskuläre Effekte [8] sowie eine beeinträchtigte Kommunikation [9], bei Kindern und Jugendlichen körperliche Beschwerden wie Hitze/Feuchtigkeit oder Kurzatmigkeit [10, 11] und bei unter 2‑Jährigen Erstickungsgefahr [12] auftreten.

In der Diskussion, insbesondere bei rückläufigen Infektionszahlen – und damit abnehmendem Nutzen von Masken in Bezug auf Infektionsübertragung – stand auch die Frage, inwiefern die psychosoziale Entwicklung von Kindern und Jugendlichen beeinträchtigt werde. Bisher existieren hier theoretische Erwartungen, basierend auf allgemeinen, wissenschaftlichen Erkenntnissen zur verbalen und nonverbalen Kommunikation [13, 14]. Dabei werden unterschiedliche Auswirkungen im familiären Umfeld [15], in der Kita [16] und im Setting Schule [17] thematisiert.

Zum spezifischen Effekt des Masketragens im Pandemiefall sind kaum empirische Forschungsbelege bekannt. Daher soll auf der Grundlage eines systematischen Reviews folgende Forschungsfrage beantwortet werden: „Wie beeinflusst das Tragen von Gesichtsmasken im öffentlichen Raum zur Prävention von Infektionskrankheiten die psychosoziale Entwicklung von Kindern und Jugendlichen?“

## Methoden

Es wurde ein systematischer Review durchgeführt, dessen Studienprotokoll bei PROSPERO (International Prospective Register of Systematic Reviews) unter der Registrierungsnummer CRD42020223217 veröffentlicht wurde [18]. Zur Gewährleistung einer hohen Berichtsqualität fand die PRISMA(Preferred Reporting Items for Systematic Reviews and Meta-Analyses)-Checkliste [19] Anwendung.

### Auswahlkriterien und Suchstrategie

Die Auswahlkriterien der Übersichtsarbeit wurden aufbauend auf dem in der evidenzbasierten Medizin angewendeten PEO-Schema (Population, Exposition, Outcome) festgelegt [20], das um die Kriterien zu den Dimensionen „Setting“ und „Studiendesign“ ergänzt wurde. Detaillierte Angaben zu den Auswahlkriterien befinden sich in Tab. 1.

**Table 1:** 

	Einschlusskriterien	Ausschlusskriterien
Population	*Kinder und Jugendliche (0–18 Jahre):*	Erwachsene (> 18 Jahre)
	Säuglinge (1. Lebensjahr)	
	Kleinkinder (2.–3. Lebensjahr)	
	Kinder in der frühen Kindheit (4.–6. Lebensjahr)	
	Kinder in der mittleren Kindheit (7.–10. Lebensjahr)	
	Kinder in der späten Kindheit (11.–14. Lebensjahr)	
	Jugendliche (15.–18. Lebensjahr)	
Setting	Öffentlicher Raum (u. a. Kindertagesstätten, Schulen, Sportvereine, Kultur- und Bildungsstätten)	Häuslich-familiäres Umfeld
		Klinisches Setting (z. B. pädiatrische Onkologie)
Exposition	*Einsatz von Gesichtsmasken im öffentlichen Raum zur Prävention von Infektionskrankheiten (sowohl im Sinne des Tragens durch Kinder und Jugendliche selbst als auch durch deren Bezugspersonen (z.* *B. Eltern, Betreuer:innen, Lehrer:innen, Erzieher:innen, Verwandte, Freund:innen, Peers))*	–
	*Infektionserkrankungen, die das Tragen von Masken im öffentlichen Raum erfordern können:*	
	Erkältung (Rhinoviren)	
	Coronavirus-Krankheit-2019 (SARS-CoV-2)	
	Schweres Akutes Respiratorisches Syndrom (SARS)	
	Middle East Respiratory Syndrome (MERS)	
	Influenza-Virus-A/H1N1 („russische Grippe“)	
	Influenza-Virus-A/H1N1 („Schweinegrippe“)	
	Influenza-Virus-A/H2N2 („asiatische Grippe“)	
	Influenza-Virus-A/H3N2 („Hongkong-Grippe“)	
	Influenza-Virus-A/H5N1 („Vogelgrippe“)	
	Influenza-Virus-A/H7N9 („Vogelgrippe“)	
	Influenza-Virus-B/Yam	
	Ebola	
	Tuberkulose	
Outcome	Psychische Entwicklung (u. a. Ängste, regressive Entwicklungen, Beeinflussung der Bindungsentwicklung bei 0‑ bis 2‑Jährigen, psychosomatische Symptome, Aggression, Zurückgezogenheit, Verhaltensstörung)	–
	Sprachentwicklung	
	Sozioemotionale Entwicklung (u. a. Emotionserkennung, zielgruppeneigene Emotionsentwicklung, Beziehungseinschätzung, zielgruppeneigene Beziehungs- und Vertrauensentwicklung)	
	Soziales Verhalten (u. a. Empathie, soziale Kompetenz)	
	Schulerfolg	
	Teilhabe	
Studiendesign	Epidemiologische Beobachtungsstudien (Kohortenstudien, Fallkontrollstudien, Querschnittsstudien)	Subjektive Studientypen (Editorials, Kommentare, Expertenmeinungen, Korrekturen, Briefe)
	Interventionsstudien (randomisierte, kontrollierte Studien, nichtrandomisierte kontrollierte Studien, kontrollierte Vorher-Nachher-Studien)	
	Klinische Beobachtungsstudien (Fallberichte, Fallserien)	
	Qualitative Studien (Interviews, Fokusgruppen)	
	Mixed-Methods-Studien	
	Experimentalstudien	
	Reviews (systematische Reviews, Rapid-Reviews, Scoping-Reviews, narrative Reviews, Metaanalysen)	
Publikationsform	Studiensprache: Deutsch, Englisch	Studiensprache: andere als Deutsch oder Englisch
	Publikationsform: Peer-Review- und Nicht-Peer-Review-Artikel	Publikationsform: Nicht-Peer-Review-Artikel
	Publikationszeitraum: ab 1980	Publikationszeitraum: vor 1980

Die Literatursuche wurde sensitiv gestaltet, um eine möglichst umfassende Identifizierung relevanter Forschungsarbeiten zu erzielen. Am 08.07.2021 wurden vier elektronische Datenbanken (PubMed, EMBASE, PsycINFO, PSYNDEX) mit Eingrenzung des Publikationszeitraums ab dem Jahr 1980 durchsucht. In den Suchen wurden Begriffe zur Population der Kinder und Jugendlichen, zur Exposition des Masketragens und zu den definierten Infektionskrankheiten miteinander verbunden. Begriffe zum Outcome wurden nicht integriert, um eine hohe Offenheit bezüglich des Endpunktes der psychosozialen Entwicklung zu gewährleisten (siehe Onlinematerial, Nr. 1). Weiterhin wurden die Preprint-Server PsyArXiv (09.07.2021), preVIEW: COVID-19 (12.07.2021) und Preprints.org (12.07.2021) nach passenden Publikationen durchsucht (siehe Onlinematerial, Nr. 1). Über Handsuchen wurden die Referenzlisten der eingeschlossenen Volltexte und von 117 themenrelevanten Artikeln (siehe Onlinematerial, Nr. 3) gesichtet. Außerdem fand eine Vorwärtssuche der eingeschlossenen Volltexte über die Web of Science Core Collection statt (siehe Onlinematerial, Nr. 2).

### Literaturauswahl, Datenextraktion, kritische Methodenbewertung

Jeweils 2 Reviewerinnen führten die *Titel- und Abstract-Sichtung *(KH und TMH) und die *Volltextsichtung* (AF und KH oder THM) unabhängig voneinander durch. Fehlende Übereinstimmungen wurden unter Einbezug einer dritten Reviewerin (KH oder THM) miteinander diskutiert. Die in der Volltextsichtung ausgeschlossenen Studien wurden einschließlich der Ausschlussgründe schriftlich tabellarisch dokumentiert (siehe Onlinematerial, Nr. 4).

Die Datenextraktion der ausgewählten Studien wurde von 2 Reviewerinnen unabhängig voneinander durchgeführt (AF und KH) und anschließend diskutiert. Dazu wurde eine standardisierte Datenextraktionstabelle verwendet. Aus den Studien wurden allgemeine Studieninformationen sowie Informationen zu Setting, Population, Exposition/Intervention, Kontrollbedingung/-intervention, Outcome, Ergebnissen sowie weiteren Angaben wie Finanzierung oder Interessenkonflikt extrahiert.

Die Methodik der Primärstudien wurde durch 2 Reviewerinnen unabhängig voneinander kritisch bewertet (AF und KH). Dazu wurde ein „Risk-of-Bias“-Verfahren in Anlehnung an Ijaz und Kuijer angewendet [21, 22]. Dabei wird das Verzerrungsrisiko für 9 Verzerrungsquellen mit „niedrig“, „hoch“ oder „unklar“ bewertet. Folgende 6 Verzerrungsquellen werden als Hauptdomänen aufgefasst: (1.) Proband:innengewinnung und (in Kohortenstudien) Follow-up, (2.) Expositionsdefinition und -messung, (3.) Outcome-Definition und -Messung, (4.) Confounding, (5.) Auswertungsmethode und (6.) Temporalität. Drei Verzerrungsquellen werden als „Nebendomänen“ gewertet: (1.) Verblindung der Untersucher:innen, (2.) Studienfinanzierung und (3.) Interessenkonflikte. Das Gesamtverzerrungsrisiko einer Studie wird bewertet, indem die Beurteilungen der 6 Hauptdomänen herangezogen werden. Erhalten alle Hauptdomänen ein niedriges Verzerrungsrisiko, ist auch das Gesamtverzerrungsrisiko einer Studie gering. Anderenfalls wird ihr Gesamtverzerrungsrisiko als hoch eingestuft. Diskordanzen bei der Bewertung wurden im Konsens gelöst und im Fall einer fehlenden Einigung wurde eine dritte Reviewerin hinzugezogen (TMH).

### Analyse und Synthese

Es wurden 2 Forschungsansätze verfolgt. Um zu untersuchen, ob ein direkter Zusammenhang zwischen dem Masketragen und der psychosozialen Entwicklung von Kindern und Jugendlichen besteht, wurden die Ergebnisse von epidemiologischen Beobachtungsstudien und von Interventionsstudien herangezogen. Um einen vertiefenden Einblick in die Thematik zu erlangen, wurden zudem im Sinne explorativer Forschung zusätzlich die Erkenntnisse aus Experimentalstudien, qualitativen Studien, Mixed-Methods-Studien und Reviews berücksichtigt. So sollen unter anderem Evidenzlücken identifiziert und Forschungsbedarfe abgeleitet werden. Neben einer tabellarischen Ergebniszusammenfassung (siehe Onlinematerial, Nr. 5 und 6) wurden die Ergebnisse jeder der 11 eingeschlossenen Einzelstudien aufgrund des hohen Verzerrungsrisikos der Primärstudien deskriptiv-narrativ aufbereitet.

## Ergebnisse

**Figure 1:**
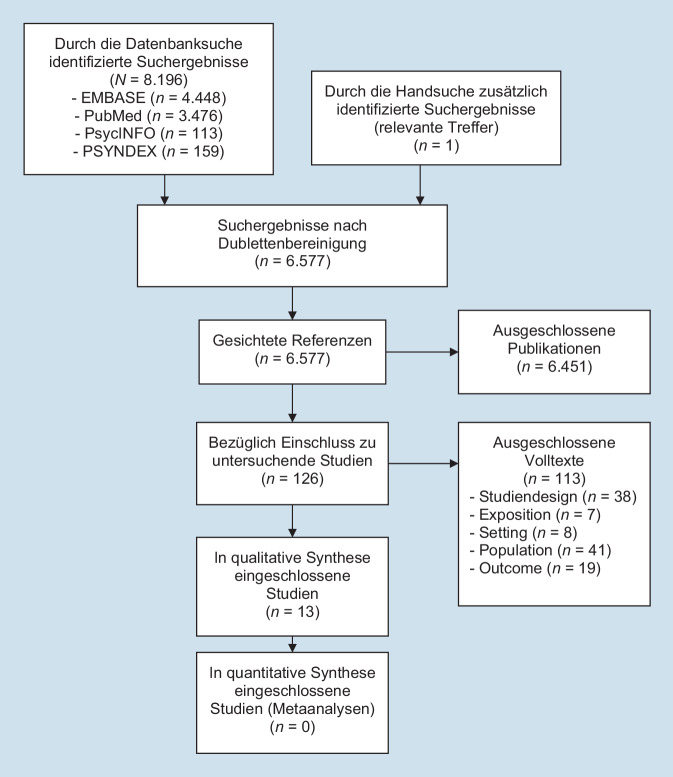

Die Ergebnisse der Literatursuche sind im PRISMA-Flussdiagramm schematisch dargestellt (Abb. 1). Der Kappa-Wert nach Cohen betrug für die Titel-Abstract-Sichtung 0,42 (deutliche Übereinstimmung) und für die Volltextsichtung 0,71 (starke Übereinstimmung; [23]).

**Figure 2:**
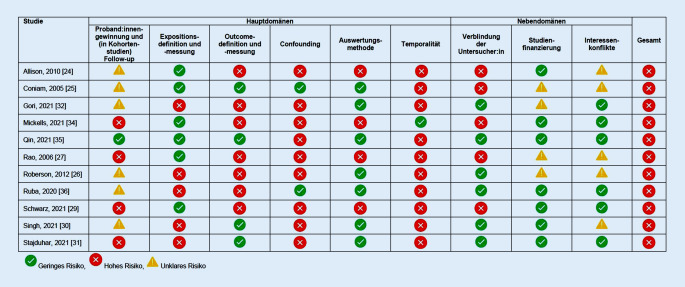

Es wurden 13 Publikationen [24–36], davon eine Längsschnittstudie [34], 2 Querschnittsstudien [29, 35], 2 Mixed-Methods-Studien [25, 27], eine unkontrollierte Interventionsstudie [24], 5 Experimentalstudien [26, 30–32, 36] und 2 Übersichtsarbeiten [28, 33], eingeschlossen. Von den Studien, die im Feld durchgeführt wurden, behandelten 3 COVID-19 [29, 34, 35], 2 das schwere akute respiratorische Syndrom (SARS; [25, 27]) und eine Influenza [24]. Alle Primärstudien wurden mit einem hohen Gesamtverzerrungsrisiko bewertet. Abb. 2 zeigt die Beurteilung des Verzerrungsrisikos der Einzelstudien.

Im Folgenden werden die Ergebnisse für jede der eingeschlossenen Studien geordnet nach Studiendesign – also Beobachtungs‑/Interventionsstudien, Experimentalstudien und Übersichtsarbeiten – deskriptiv dargestellt. Eine genaue Auflistung aller relevanten, studienspezifischen Daten erfolgt zudem im Onlinematerial, Nr. 5 und 6.

### Beobachtungs‑/Interventionsstudien

#### COVID-19

- Längsschnittstudie von Mickells et al. (2021; [34])
  In der Längsschnittstudie von Mickells et al. wurde über einen vierwöchigen Befragungszeitraum zu Beginn des Schuljahres 2020/2021 von den teilnehmenden Erzieher:innen und Lehrer:innen die Adhärenz zum Masketragen der Kinder in den Kinderkrippen, Kindergärten und der 1. und 2. Klasse der Grundschulen im gesamten katholischen Schulbezirk in Atlanta, USA, berichtet, in dem eine Maskenpflicht für alle Kinder bestand [34]. Die Masken wurden nach Angaben des pädagogischen Personals von den Kindern die meiste Zeit des Tages (76,9 % der beobachteten Zeit) ordnungsgemäß getragen. Im Rahmen der Untersuchung wurden auch unerwünschte Ereignisse im Zusammenhang mit dem Masketragen erfasst. Am häufigsten wurden von den 92 Befragten Stresserleben, Angst und psychische Zusammenbrüche bei den Kindern (*n* = 13) angegeben. Auch trat Frustration auf, wenn sie das, was durch eine Maske gesprochen wurde, nicht gut verstehen konnten oder die Kommunikation mit Erzieher:innen bzw. Lehrer:innen und Mitschüler:innen durch das Masketragen erschwert wurde (*n* = 8).
- Querschnittsstudie von Qin et al. (2021; [35])
  Qin et al. führten im März 2020 in der Guangdong-Region in China eine Befragung bei schulpflichtigen Kindern und Jugendlichen (*n* = 1.199.320) zur psychischen Belastung während der COVID-19-Pandemie durch [35]. Dabei fand sich ein negativer Zusammenhang zwischen dem Tragen von Masken und dem Auftreten einer psychischen Belastung. Kinder und Jugendliche, die angaben, niemals eine Maske zu tragen, hatten ein 2,64-fach erhöhtes Risiko (95 % Konfidenzintervall: 2,45–2,83) für eine psychische Belastung im Vergleich zu Kindern und Jugendlichen, die immer eine Maske trugen.
- Querschnittsstudie von Schwarz et al. (2021; [29])
  Mittels einer Querschnittsbefragung wurde ein deutschlandweites Onlineregister für die Zielpopulation der Kinder und Jugendlichen aufgebaut, das unter anderem subjektive Beschwerden des Masketragens abbilden soll. Dazu wurden Eltern, Ärzt:innen, Pädagog:innen und andere Personen befragt, wobei sich die hier vorgestellte Studie von Schwarz et al. auf die Angaben der Elternschaft (Befragungszeitraum: 20.–26.10.2020) bezieht. Insgesamt liegen Werte zu 25.930 Kindern und Jugendlichen im Alter von 0–17 Jahren vor, die von 17.854 Eltern eingetragen wurden. Im Hinblick auf die schulische Leistungsfähigkeit gaben die Eltern an, dass ihre Kinder unter Konzentrationsschwierigkeiten (49,5 %) bzw. Beeinträchtigung des Lernens (38,0 %) aufgrund des Masketragens litten. Verhaltensauffälligkeiten durch das Tragen von Masken waren (beginnend mit der am häufigsten genannten Störung): Gereiztheit, verminderte Fröhlichkeit, der Unwille, in die Schule oder den Kindergarten zu gehen, schlechterer Schlaf, Unruhe, Ängste, vermehrter Schlaf, geringeres Spielverhalten und größerer Bewegungsdrang. Über Freitextangaben wurden zusätzlich durch das Masketragen auftretende Ängste konkretisiert, unter anderem Zukunftsangst, Angst vor Erstickung oder Stigmatisierung durch das (Nicht‑)Tragen der Masken. Außerdem berichten Eltern von Albträumen und Angststörungen ihrer Kinder, welche sich auf maskierte Menschen beziehen, deren Mimik und Identität für die Kinder nicht erkennbar sind.

#### Influenza

##### Interventionsstudie von Allison et al. (2010; [24]).

Ähnliche Ergebnisse zeigte eine Interventionsstudie in 2 Grundschulen in den USA, die in einer vierwöchigen Interventionsphase sowohl die Nutzung von Händedesinfektion als auch das Tragen einer Maske als Influenzapräventionsmaßnahmen bei Kindern und Lehrer:innen untersuchten [24]. Die Studie gliederte sich in 2 Phasen: In den ersten 2 Wochen wurde zur Nutzung von Händedesinfektion angeregt. In den anschließenden 2 Wochen sollten Kinder und Lehrer:innen unabhängig von ihrem Infektionsstatus eine Maske tragen. Dazu wurden täglich neue Masken zur Verfügung gestellt. In der ersten Woche trugen noch 63 % der Lehrkräfte und 30 % der Schüler:innen eine Maske, in der zweiten Woche waren es noch 38 % der Lehrkräfte und 15 % der Schüler:innen. Als Hauptgründe für die Ablehnung wurden Schwierigkeiten beim Erkennen der Mimik und körperliches Unbehagen beim Tragen benannt (unter anderem sei die Maske störend, zu warm oder führe zu Juckreiz auf der Haut).

#### Schweres akutes respiratorisches Syndrom (SARS)

- Mixed-Methods-Studie von Coniam (2005; [25])
  Die Auswirkungen des Tragens von Masken auf Prüfungsleistungen im Zuge der SARS-Pandemie in Hongkong untersuchte Coniam [25] an Schülerinnen (*n* = 186) der 11. Klassenstufe einer Mädchenschule. Diese absolvierten mündliche Englischprüfungen, bestehend aus Rollenspielen und Gruppendiskussionen, je einmal mit und einmal ohne OP-Masken. Beide Bedingungen wurden im Hinblick auf die erbrachte mündliche Testleistung, die persönliche Einschätzung der Leistung durch die Prüflinge, die Sprachlautstärke und das Sprechtempo sowie die nonverbale Kommunikation verglichen. Die Testleistungen zeigten auf keiner der zu bewertenden Dimensionen (Verständlichkeit, Aussprache, Hörbarkeit, Vokabular und Grammatik) einen Unterschied. Damit hatte das Tragen der Masken keinen abträglichen Effekt auf die Prüfleistung. Die Geprüften selbst erlebten ihre mündlich erbrachten Englischleistungen allerdings in der Maskensituation als schlechter. Die Mehrzahl der geprüften Schülerinnen gab an, beim Tragen von Masken langsamer und lauter gesprochen zu haben. Auch die Untersucher:innen beschrieben, dass in der Prüfungssituation mit Masketragen von den Geprüften verschiedene Kompensationsstrategien, wie zum Beispiel lauteres Sprechen, intensiverer Augenkontakt oder eine vermehrte Körpersprache, zum Einsatz kamen. Als ein weiterer Befund der Studie wurde berichtet, dass es einigen der Schülerinnen beim Tragen der Masken schwerfiel, die Gesichter der anderen zu erkennen.
- Mixed-Methods-Studie von Rao et al. (2006; [27])
  Während der SARS-Epidemie wurden von März bis Mai 2003 die Schulen und Vorschulen in Hongkong geschlossen. Während der Wiederöffnungsphase mussten Kinder und Erzieher:innen eine Maske tragen. Die Mixed-Methods-Studie untersuchte in dieser Situation, ob sich die Tagesabläufe und das Verhalten von Kindern im Alter von 6 Wochen bis 6 Jahren in Vorschulen verändert hatten [27]. Dazu wurden Beobachtungen in 20 Vorschulen und eine Umfrage mit 10 Rektor:innen durchgeführt. Es zeigte sich ein Einfluss des Masketragens auf die Interaktionen zwischen Erzieher:innen und Kindern sowie zwischen den Kindern untereinander, da sie Gesichtsausdrücke aufgrund der Masken nicht so gut erkannten. Die Kinder erlebten das Sprechen aufgrund der Masken als unangenehm.

### Experimentalstudien

#### Fähigkeit zur Gesichtserkennung

- Experimentalstudie von Gori et al. (2021; [32])
  Gori et al. untersuchten im Rahmen ihrer Experimentalstudie, ob durch das Tragen einer Maske die Fähigkeit zur Deutung von Emotionen anhand von Gesichtsausdrücken bei Kindern im Alter von 3–5 und 6–8 Jahren sowie bei Erwachsenen beeinträchtigt wurde [32]. Dazu wurden ihnen über ein Smartphone Bilder von Gesichtern mit oder ohne Maske präsentiert, welche folgende Gefühle in 2 Intensitätsstufen (schwach oder stark ausgeprägt) darstellten: Freude, Wut, Traurigkeit, Angst und neutraler Gesichtsausdruck. Es zeigte sich ein statistisch signifikanter Effekt des Masketragens (F(1,116) = 48,7, *p* < 0,001), wobei in allen Altersklassen der Anteil richtiger Zuordnungen für die Bilder mit Maske geringer war als für die Bilder ohne Maske (Kinder 3–5 Jahre: t(30) = 11,94, *p* < 0,001, Kinder 6–8 Jahre: t(30) = 4,61, *p* < 0,001, Erwachsene: t(30) = 8,1, *p* < 0,001). Der negative Effekt des Masketragens war bei Kleinkindern statistisch signifikant stärker ausgeprägt im Vergleich zu Grundschulkindern oder Erwachsenen. Allerdings gab es dahin gehend keinen Unterschied zwischen Grundschulkindern und Erwachsenen.
- Experimentalstudie von Roberson et al. (2012; [26])
  Die Fähigkeit von Kindern (und Erwachsenen) Gesichtsausdrücke zu erkennen, war Untersuchungsgegenstand in der Experimentalstudie von Roberson et al. [26]. Dazu wurden jeweils 20 Mädchen im Alter von 3–4, 5–6, 7–8 und 9–10 Jahren (sowie erwachsenen Frauen und Männern) Fotoaufnahmen von Gesichtern mit den folgenden 5 Emotionen gezeigt: Freude, positives Erstaunen/Überraschung, Ärger, Angst und Traurigkeit. Der Emotionsgrad auf den Bildern betrug jeweils 100 %, 80 % oder 60 %, wobei bei 100 % die Gesichtsmimik hinsichtlich einer Emotion am stärksten und bei 60 % am geringsten ausgeprägt war. Die Gesichter der Personen auf den Bildern waren entweder unbedeckt oder die Personen trugen eine dunkle Maske über der Mundregion oder eine Sonnenbrille. Den Kindern wurde für jedes Bild eine Emotion vorgegeben und sie mussten entscheiden, ob diese Emotion bei der Person auf dem Bild zutraf oder nicht. Ob eine Emotion richtig zugeordnet wurde, hing statistisch signifikant von der Testbedingung (unbedecktes Gesicht oder Masketragen) ab (F(4,190) = 9,74, MSe = 0,03, *p* < 0,01). Es zeigte sich, dass bei den Kindern, die 8 Jahre oder jünger waren, der Anteil korrekter Zuordnungen zu einer Emotion unabhängig davon war, ob die Person auf dem Bild unbedeckt war oder ob sie eine Maske trug. Im Gegensatz dazu war der korrekte Zuordnungsgrad bei 9‑ bis 10-Jährigen und Erwachsenen dann höher, wenn die Gesichter unbedeckt waren. Dies deutet darauf hin, dass jüngere Kinder andere Strategien der Gesichtsverarbeitung nutzen als ältere Kinder und Erwachsene und daher bei der Emotionserkennung durch eine Maske weniger beeinträchtigt werden.
- Experimentalstudie von Ruba und Pollack (2020; [36])
  Die Experimentalstudie von Ruba und Pollack [36], die im Zusammenhang mit dem Masketragen während der COVID-19-Pandemie entstand, untersuchte ebenfalls die Fähigkeit von Kindern, unter verschiedenen Bedingungen Emotionen von Gesichtern abzulesen. Als Proband:innen dienten 81 schulpflichtige Kinder im Alter von 7 bis 13 Jahren. Diese sahen Bilder von Menschen mit den Gesichtsausdrücken „Traurigkeit“, „Wut“ oder „Angst“, wobei die Gesichter unbedeckt, mit einer OP-Maske oder einer Sonnenbrille bedeckt waren. Die Kinder sollten die Gesichtsausdrücke den folgenden Emotionen zuordnen: „glücklich“, „traurig“, „wütend“, „überrascht“, „ängstlich“ oder „angewidert“. Insgesamt konnten die 3 Gesichtsausdrücke besser identifiziert werden (F(2,154) = 27,19, *p* < 0,001, η_p_^2^ = 0,26), wenn die Person auf dem Foto keine Gesichtsbedeckung trug. Ein Unterschied zwischen dem Tragen von OP-Masken oder Sonnenbrille gab es nicht. Je mehr visuelle Informationen den Kindern zur Verfügung standen – im Sinne eines höheren Auflösungsgrades eines Bildes – desto genauer erfolgte die Zuordnung (F(18,1372) = 10,27, *p* < 0,001, η_p_^2^ = 0,12).
- Experimentalstudie von Stajduhar et al. (2021; [31])
  Stajduhar et al. untersuchten mit dieser Experimentalstudie, ob das Tragen von Masken einen Einfluss auf die Fähigkeit zur Gesichtserkennung bei 72 Schulkindern im Alter von 6 bis 14 Jahren hat [31]. Die Kinder wurden über eine Zufallsauswahl auf die Expositionsgruppe (Masketragen) oder die Vergleichsgruppe (kein Masketragen) verteilt. Sie führten von zu Hause aus auf einem Computerbildschirm einen Test basierend auf dem sogenannten Cambridge Face Memory Test (in der Version für Kinder) durch, wobei die Gesichter von 4 Jungen unter verschiedenen Bedingungen (Standpunkt, Lichtverhältnisse, visuelles Rauschen) wiedererkannt werden mussten. Das Zielkriterium war der Grad der Genauigkeit des Wiedererkennens auf einer Skala von 0 bis 48. Kinder unter 10 Jahren durften beim Lesen der Studienanweisungen von ihren Eltern unterstützt werden. Waren die Gesichter auf den Bildern maskiert, war die Fähigkeit zur Gesichtserkennung reduziert (F(1,68) = 14,31, *p* < 0,001, ηp^2^ = 0,17). Dieser Effekt des Masketragens war für verschiedene Altersgruppen – also jüngere bzw. ältere Kinder – ähnlich groß.

#### Fähigkeit zur Spracherkennung

##### Experimentalstudie von Singh et al. (2021; [30]).

In einer Experimentalstudie überprüften Singh et al., ob die Fähigkeit zum Erkennen von Wörtern bei 24 Jungen und Mädchen im Alter von 2 Jahren durch das Tragen von Masken beeinträchtigt wurde [30]. Dazu wurde den Kindern über einen Computerbildschirm das Gesicht einer Frau präsentiert, das entweder a) unbedeckt war bzw. b) eine OP-Maske oder c) einen transparenten Gesichtsschutz (Visier) trug. Die Frau sprach 1 von 18 einsilbigen Zielwörtern aus (Auswahl aus früh erworbenen Wörtern bei einsprachigen englischen Kindern), stets verbunden mit der Einleitungsfrage: „Can you see the …?“ Gleichzeitig erschienen auf dem Bildschirm 2 Bilder, eines, das das Zielwort abbildete, und eines, das den Gegenstand des Ablenkungswortes zeigte. Über die Bewegung der Augen, aufgenommen über eine Kamera, wurde ermittelt, ob die Kinder nach Aussprechen des Wortes das Zielbild länger als vor dem Aussprechen des Wortes fixierten, was für das Erkennen eines Wortes spricht. In den Situationen, in denen die Frau auf dem Bildschirm keine Maske (t(23) = 3,01, *p* = 0,006, Cohens d = 0,93) bzw. eine OP-Maske (t(23) = 3,51, *p* = 0,002, Cohens d = 0,86) trug, wurden die Zielbilder nach Aussprechen des Wortes statistisch signifikant länger fixiert. Dies war nicht der Fall, wenn die Frau einen transparenten Visor trug (t(23) = 0,71, *p* = 0,49). Weiterhin lag kein statistisch signifikanter Unterschied der Fähigkeit der Worterkennung vor, wenn die Frau keine Maske oder eine OP-Maske trug (F(1,22) = 0,57, *p* = 0,46), wohingegen ein Unterschied bestand zwischen dem Zustand „keine Maske“ und dem Zustand „Visor“ (F(1,22) = 7,63, *p* = 0,01, ηp^2^ = 0,26). Die Autor:innen der Studie gehen davon aus, dass bestimmte Oberflächeneigenschaften eines transparenten Visors wie Lichtbrechung und Reflexionen die Übertragung visueller Informationen einschränken und damit das visuelle Erkennen der Aussprache erschweren.

### Übersichtsarbeiten

#### Übersichtsarbeit von Kisielinski et al. (2021; [33])

Die Übersichtsarbeit von Kisielinski et al. suchte nach Studien zu nachteiligen Auswirkungen des Tragens von Masken in PubMED (Medline; [33]). Aus dem pädiatrischen Bereich identifizierten sie eine Studie, die sich mit den psychosozialen Folgen des Masketragens für Kinder und Jugendliche beschäftigte. Dabei handelt es sich um die Querschnittstudie von Schwarz et al. ([29]; s. oben). Die Autor:innen des Reviews schlussfolgern, basierend auf den in ihrem Review eingeschlossenen Publikationen, dass die durch das Masketragen verminderte verbale und nonverbale Kommunikation und die damit einhergehende eingeschränkte soziale Interaktion für Kinder schwerwiegende Folgen haben können. Weitere Forschung ist daher insbesondere für diese vulnerable Population notwendig.

#### Übersichtsarbeit von Sim et al. (2014; [28])

Der Literaturreview von Sim et al. [28] identifizierte keine einzige Studie zur Beeinflussung der psychosozialen Entwicklung von Kindern und Jugendlichen durch die Pflicht zum Tragen von Masken im öffentlichen Raum.

## Diskussion

Die umfassende Literatursuche identifizierte *13 Forschungsarbeiten*, die die Auswirkungen des Masketragens zur Prävention von Infektionen auf die psychosoziale Entwicklung von Kindern und Jugendlichen untersuchten [24–36]. Das Gesamtverzerrungsrisiko wurde in allen eingeschlossenen Primärstudien als hoch eingeschätzt.

3 Studien lieferten Informationen zu psychosozialen Effekten durch das Masketragen während der COVID-19-Pandemie [29, 34, 35], wobei 2 Studien über negative Folgen des Masketragens berichten wie Ängste [29, 34], Stresserleben, psychische Zusammenbrüche, Frustration wegen behinderter Kommunikation [34] sowie stärkere Gereiztheit und verschlechterte Stimmungslage (vor allem bei den älteren Kindern), Unruhe und schlechterer Schlaf [29]. Im Gegensatz dazu ermittelten Qin et al. [35] einen negativen Zusammenhang zwischen Masketragen und psychischen Belastungen. Dieses Ergebnis könnte nach Meinung der Studienautor:innen daraus resultieren, dass Schüler:innen, die häufiger eine Maske trugen, es für weniger wahrscheinlich hielten, sich mit COVID-19 zu infizieren, was wiederum Gefühle von Sorge und Ängsten senken und damit die psychische Gesundheit steigern könnte.

Bei der Interpretation dieser Ergebnisse sollte nicht außer Acht gelassen werden, dass in den beiden Studien, die abträgliche Effekte des Masketragens fanden, nicht die Kinder und Jugendlichen selbst, sondern deren Pädagog:innen bzw. Eltern befragt wurden und dass zudem in der Studie von Schwarz et al. [29] die Symptome und Verhaltensänderungen über Kausalattribution erhoben wurden: Eltern wurden gebeten, Folgen und Nebenwirkungen des Masketragens zu benennen. Damit nehmen sie implizit eine subjektive Ursachenzuschreibung vor. Außerdem ist zu berücksichtigen, dass während des Befragungszeitraumes neben dem Masketragen weitere Maßnahmen der Pandemiebekämpfung wie Social Distancing (z. B. Homeschooling) relevant waren. Beobachtete Effekte wie verschlechterte Stimmungslage in einem solch komplexen Gefüge an Maßnahmen ausschließlich auf das Masketragen zurückzuführen, könnte möglicherweise zu einer Überschätzung von Effekten führen.

Mit Blick auf schulische Leistungsfähigkeit ihrer Kinder berichteten Eltern in der Studie von Schwarz et al. durch die Maske Konzentrationsschwierigkeiten und Beeinträchtigungen beim Lernen, vor allem bei älteren Kindern. Die Studie von Coniam zeigte, nicht für die Lern-, sondern die Testsituation, dass das Tragen eines Mund-Nasen-Schutzes keinen Einfluss auf die objektiv gemessenen *Prüfungsleistungen* bei einem mündlichen Sprachtest hatte, auch wenn die Geprüften selbst ihre Leistung beim Tragen der Maske schlechter einschätzten als in der Situation ohne Maske [25].

In 7 Studien wurde das Erkennen von Gesichtsausdrücken und damit verbundenen Emotionen untersucht [24–27, 31, 32, 36]. Diese wichtige Determinante sozialer Interaktion war damit der am häufigsten untersuchte Aspekt psychosozialer Entwicklung im Zusammenhang mit dem Masketragen. In 3 dieser Studien berichteten Pädagog:innen aus dem Vorschul- bzw. Schulalltag sowie Schülerinnen in Prüfungssituationen, dass das Masketragen das Lesen von Gesichtsausdrücken sowohl bei den Kindern als auch Betreuungspersonen negativ beeinträchtigt habe und damit die soziale Interaktion gestört worden sei [24, 25, 27]. Vier Experimentalstudien untersuchten die Wirkung des Masketragens auf die Fähigkeit des Erkennens von Emotionen bei Kindern unterschiedlicher Altersgruppen. Während Ruba und Pollack [36] sowie Stajduhar et al. [31] übereinstimmend (bei 7‑ bis 13-Jährigen bzw. 6‑ bis 14-Jährigen) fanden, dass den Kindern das Erkennen von Gesichtsausdrücken unabhängig vom Alter besser gelang, wenn die abgebildeten Personen keine Maske trugen, untersuchten Gori et al. [32] und Roberson et al. [26] auch jüngere Kinder (ab 3 Jahre) sowie Erwachsene. Mit Blick auf jüngere Kinder kamen sie zu unterschiedlichen Ergebnissen: In einer Studie hatten besonders die jüngeren Kinder Probleme bei der Emotionserkennung in der Maskenbedingung [32], während es in der anderen Studie gerade bei den jüngeren Kindern keinen Unterschied in der Genauigkeit der Zuordnung machte, ob die betrachtete Person eine Maske trug oder nicht [26]. Erst ab einem Alter von 9 Jahren konnten Emotionen besser von Gesichtern ohne Maske abgelesen werden. Die Autoren [26] schlussfolgerten, dass das Entschlüsseln von Gesichtsausdrücken bis zum 7. Lebensjahr vorranging auf der Augenpartie beruht, so dass die Bedeckung anderer Teile des Gesichtes – unter anderem des Mund-Nasen-Bereiches – nur einen geringen Einfluss auf diesen Prozess hat. Demgegenüber erklären Gori et al. [32] ihre Befunde der schlechteren Emotionserkennung mit Maske bei jüngeren Kindern damit, dass sich vor allem Kleinkinder noch in der emotionalen Entwicklung befinden. Es stellt sich die Frage, ob durch das Tragen von Masken die Entwicklung sozialer Kompetenzen von Kindern gestört oder verzögert werden könnte.

Grundsätzlich weisen Ruba und Pollack [36] jedoch darauf hin, dass das Erkennen von Emotionen durch das Tragen eines Mund-Nasen-Schutzes zwar erschwert wird, den Kindern aber dennoch gelingt. Schließlich werde die Fähigkeit der Emotionserkennung durch weitere Faktoren, wie z. B. Umgebungskontext, Körperhaltung oder Stimmgebung, beeinflusst. Zusammenfassend leiten sie daher ab, dass das Deuten von Emotionen und die damit verbundene soziale Interaktion von Kindern in Zeiten der COVID-19-Pandemie nicht wesentlich beeinträchtigt sind.

Bei der Interpretation der Ergebnisse dieser Experimentalstudien sollten folgende Punkte berücksichtigt werden: (1.) Es handelt sich um ein experimentelles Setting, in dem den Teilnehmenden lediglich Fotoserien präsentiert wurden. Eine Generalisierung der Ergebnisse auf das reale Leben ist daher nur sehr eingeschränkt möglich. (2.) In den Studien von Ruba und Pollack [36] und Stajduhar et al. [31], in denen ältere Kinder untersucht wurden, war die Altersspanne wesentlich größer, während in den Studien, die auch jüngere Kinder ab 3 Jahren betrachteten [26, 32] die Altersspanne der Untersuchungsgruppen kleiner war. Durch weiter gefasste Altersgruppen könnte gegebenenfalls die Varianz des untersuchten Merkmals verloren gehen. (3.) In den Studien wurden unterschiedlich viele Emotionen (3–5) präsentiert.

Zur Verbesserung des Erkennens von Gesichtsausdrücken schlagen Gori et al. Masken vor, die eine Sichtbarkeit des Gesichtes ermöglichen [32]. Dass die Sicht auf das Gesicht dabei nicht unbedingt besser sein muss, zeigen Ergebnisse der Experimentalstudie von Singh et al. [30], in der die Fähigkeit zur Spracherkennung durch das Masketragen untersucht wurde. Hier waren transparente Schutzvorrichtungen, sogenannte Visoren, mit einer verminderten Fähigkeit zur Worterkennung bei Kleinkindern verbunden. Demgegenüber verstanden die Kinder die Sprechenden gleich gut, wenn diese keine Maske oder eine OP-Maske trugen.

Aufgrund der eher geringen Studienzahl, der Fokussierung auf die Emotionserkennung und der fehlenden Datengrundlage für eine Vielzahl anderer psychosozialer Endpunkte kann die Forschungsfrage des vorliegenden Reviews nur eingeschränkt beantwortet werden. Daraus ergeben sich Hinweise für Forschungsbedarfe.

## Forschungslücken und Forschungsbedarf

Die Primärstudien beschäftigten sich nicht mit dem Einfluss des Masketragens auf die Beziehungs‑/Vertrauensentwicklung sowie die Entwicklung sozialer Kompetenz und des Sozialverhaltens und die Teilhabe von Kindern und Jugendlichen. Auch zum Schulerfolg konnten (außer einer Studie zur Prüfungsleistung während eines mündlichen Sprachtests und Elternberichten über Einschränkungen in Lernen und Konzentration) keine Forschungsarbeiten identifiziert werden. Empirische Studien zu Neugeborenen und Säuglingen fehlen, wobei für diese Population die Auswirkungen des Tragens von Masken auch eher im familiären oder klinischen Umfeld als im öffentlichen Raum zu erwarten sind. Themenrelevante Studien aus dem außerschulischen Bereich, wie z. B. Sportvereine sowie Kultur- und Bildungsstätten, wurden nicht aufgefunden. Im Hinblick auf die untersuchten Infektionskrankheiten liegen keine Veröffentlichungen zu Erkältungskrankheiten, MERS (Middle East Respiratory Syndrome), Ebola und Tuberkulose vor. Es fehlen Studien, die das längerfristige „Belästigungsempfinden“ und längerfristige Auswirkungen auf die kindliche Entwicklung untersuchen, wobei eine Adaptation möglich erscheint.

Es fehlen Studien, in denen die Auswirkungen des Masketragens auf die psychosoziale Entwicklung (auch aus längsschnittlicher Perspektive) von der betroffenen Population selbst berichtet werden, da die Angaben in den eingeschlossenen Feldstudien größtenteils nicht über Angaben der Kinder und Jugendlichen selbst, sondern über deren Erzieher:innen, Lehrer:innen oder Eltern gewonnen wurden. Auch qualitative Studien, in denen Expert:innen aus den verschiedenen Bereichen des öffentlichen Raums zur möglichen Beeinflussung der psychosozialen Entwicklung von Kindern und Jugendlichen durch die Pflicht zum Tragen von Masken befragt werden (wie Lehrer:innen, Erzieher:innen oder Entwicklungspsycholog:innen), könnten Hinweise geben, die über den Endpunkt Emotionserkennung hinausgehen.

Eine ausführliche Methodendiskussion des systematischen Reviews befindet sich im Onlinematerial Nr. 7.

## Schlussfolgerungen

Über Auswirkungen des Masketragens im öffentlichen Raum zur Prävention von Infektionserkrankungen auf verschiedene Entwicklungsbereiche von Kindern und Jugendlichen gibt es vor allem seit der COVID-19-Pandemie erste, aber dennoch bislang wenige Erkenntnisse, die angesichts eines recht hohen Verzerrungsrisikos nur eingeschränkt aussagekräftig sind. Die geringe Anzahl von Studien zeigt, dass dieses Thema für Kinder und Jugendliche im öffentlichen Raum kaum Relevanz hatte. Dass die Hälfte der eingeschlossenen Studien erst 2021 erschienen, weist auf die steigende Bedeutung des Forschungsthemas durch die Pandemiesituation hin. Es gibt Hinweise darauf, dass die Fähigkeit zum Lesen der Mimik durch das Masketragen von Kindern/Jugendlichen und ihren Betreuer:innen im Vorschul- und Schulsetting als beeinträchtigt erlebt wird. Diese Beeinträchtigung könnte die soziale Interaktion stören. Diese Beobachtungen wurden in mehreren Experimentalstudien bestätigt. Ob dieser Effekt als schwerwiegend angesehen werden kann, ist jedoch fraglich, da die Fähigkeit zum Deuten von Emotionen abgesehen vom Lesen der Mimik im Bereich Mund und Nase auch über die Berücksichtigung der Augenpartie und durch weitere Faktoren (unter anderem Stimmgebung, Körperhaltung und Umgebungskontext) bestimmt wird. 2 Feldstudien zeigen negative, psychische Folgen des Masketragens für Kinder und Jugendliche während der COVID-19-Pandemie u. a. in Form von Stresserleben, Ängsten, verschlechterter Stimmungslage, Konzentrationsschwierigkeiten und beeinträchtigtem Lernen. Es fehlen Forschungsdaten zu den längerfristigen Folgen des Masketragens in der Öffentlichkeit auf die Endpunkte psychische Entwicklung, Sprachentwicklung, sozioemotionale Entwicklung, soziales Verhalten, Schulleistungen und Teilhabe im Kindes- und Jugendalter.

Dennoch finden sich erste Empfehlungen zum Umgang mit Einschränkungen und Belastungen. Um mögliche Probleme mit der Sprachverständlichkeit und Höranstrengungen durch das Tragen von Masken in der Lernumgebung zu minimieren, sollten nach den Resultaten einer Experimentalstudie, die allerdings mit Studierenden und nicht mit Schulkindern durchgeführt wurde, OP-Masken und N95-Masken (Äquivalent dazu nach Europäischer Norm ist die FFP2-Atemschutzmaske) anstatt Alltagsmasken genutzt werden [37]. So ergaben Letztere eine signifikant größere Einschränkung der Sprachverständlichkeit im Vergleich zu medizinischen Masken. Alternativ wäre der Einsatz von transparenten Trennwänden in Erwägung zu ziehen [38]. Nobrega et al. formulierten praktische Empfehlungen für Eltern und Lehrkräfte, um Beeinträchtigungen der Schulleistungen durch das Masketragen entgegenzuwirken. Hilfreich seien unter anderem langsameres und lauteres Sprechen, der Einsatz von visuellen Hilfen und eine Minderung des Umgebungslärms vonseiten der Lehrkräfte. Darüber hinaus empfehlen die Autor:innen eine regelmäßige Überprüfung der Schulleistung sowie die Beobachtung von Verhaltensänderungen bei den Kindern [14].

Bei der Planung und Umsetzung von Public-Health-Maßnahmen sollten neben Befunden zu Folgen des Masketragens auf verschiedene Entwicklungsbereiche Erkenntnisse zu Einflussfaktoren auf die Compliance des Masketragens berücksichtigt werden [28]. Schulen können schon den Jüngsten die Relevanz von Masken sowie das richtige Trageverhalten vermitteln [28]. Angesichts des Mangels an Daten zum Einfluss des Masketragens auf das Leben und die Entwicklung von Kindern sollten clusterrandomisierte kontrollierte Studien (RCTs) oder methodisch hochwertige Studien im Längsschnitt durchgeführt werden.

## Supplementary Information




