# Supplementary material for: Impact of wearing face masks in public to prevent infectious diseases on the psychosocial development in children and adolescents: a systematic review
Source: Bundesgesundheitsblatt Gesundheitsforschung Gesundheitsschutz. 2021 Oct 25;64(12):1592–602. [Article in German] doi: 10.1007/s00103-021-03443-5 (PMC8543771; doi:10.1007/s00103-021-03443-5)
Supplement: Supplementary file 1 [file 103_2021_3443_MOESM1_ESM.docx]

**Bundesgesundheitsblatt 12/2021**

Onlinematerial zum Beitrag:

**Beeinflussung der psychosozialen Entwicklung von Kindern und Jugendlichen durch das Tragen von Gesichtsmasken im öffentlichen Raum zur Prävention von Infektionskrankheiten: Ein systematischer Review**

Alice Freiberg^1^, Katy Horvath^2^, Taurai Monalisa Hahne^3^, Stephanie Drössler^1^, Daniel Kämpf^1^, Anke Spura^4^, Bernhard Buhs^4^, Nadine Reibling^4^, Freia De Bock^4^, Christian Apfelbacher^3^, Andreas Seidler^1^

^1^ Institut und Poliklinik für Arbeits- und Sozialmedizin, Medizinische Fakultät Carl Gustav Carus, Technische Universität Dresden, Dresden, Deutschland

^2^ Klinik für Kinder- und Jugendpsychiatrie, Psychosomatik und Psychotherapie, Helios Park-Klinikum Leipzig, Leipzig, Deutschland

^3^ Institut für Sozialmedizin und Gesundheitssystemforschung, Medizinische Fakultät, Otto-von-Guericke Universität Magdeburg, Magdeburg, Deutschland

^4^ Bundeszentrale für gesundheitliche Aufklärung, Köln, Deutschland

**Korrespondenzadresse:**

Dr. rer. medic. Alice Freiberg, MPH

Institut und Poliklinik für Arbeits- und Sozialmedizin

Medizinische Fakultät Carl Gustav Carus

Technische Universität Dresden

Fetscherstraße 74

01307 Dresden

Deutschland

[alice.freiberg@tu-dresden.de](mailto:alice.freiberg@tu-dresden.de)

Inhalt:

1. Suchstrings in Datenbanken

2. Für Vorwärtssuche genutzte Studien

3. Für Referenzlistensuche genutzte Studien

4. Ausgeschlossene Volltexte mit Ausschlussgrund

5. Datenextraktion der Primärstudien

6. Datenextration der Reviews

7. Methodendiskussion des systematischen Reviews

## Suchstrings in Datenbanken

## Suchstring MEDLINE via PubMed

| **Nr.** | **Suchterme** |
| --- | --- |
| **1** | newborn*[tw] OR "infant, newborn"[mh] OR neonat*[tw] OR baby[tw] OR babies[tw] OR toddler*[tw] OR kindergartner*[tw] OR preschool*[tw] OR pre-school*[tw] OR schoolchild*[tw] OR schoolkid*[tw] OR pupil*[tw] OR student*[tw] OR students[mh] OR preteen*[tw] OR pre-teen*[tw] OR teen*[tw] OR adolescen*[tw] OR adolescent[mh] OR puberty[tw] OR puberty[mh] OR pubescent*[tw] OR youth*[tw] OR young*[tw] OR juvenile*[tw] OR infan*[tw] OR infant[mh] OR child*[tw] OR child[mh] OR kid[tw] OR kids[tw] OR boy[tw] OR boys[tw] OR girl[tw] OR girls[tw] OR pediatric*[tw] OR paediatric*[tw] OR minor*[tw] OR minors[mh] OR underage*[tw] |
| **2** | mask*[tw] OR masks[mh] OR facemask*[tw] OR “face pack”[tw] OR “face packs”[tw] OR “face guard”[tw] OR “face guards”[tw] OR “face covering”[tw] OR “face coverings”[tw] OR “face coverage”[tw] OR “face coverages”[tw] OR “face shield”[tw] OR “face shields”[tw] OR “faceshield*”[tw] OR “face piece”[tw] OR “face pieces”[tw] OR facepiece*[tw] OR visor*[tw] OR veil*[tw] OR glasses[tw] OR “eye protection”[tw] OR “eye guard”[tw] OR “eye shield”[tw] OR eyewear[tw] OR goggles[tw] OR "Eye Protective Devices"[Mesh] OR "Personal Protective Equipment"[mh] OR “protective equipment”[tw] |
| **3** | "respiratory tract infections"[mh] OR "respiratory tract infections"[tw] OR rhinovirus[mh] OR (respiratory[tw] AND (infect*[tw] OR illness[tw] OR symptom*[tw] OR virus*[tw] OR syndrome[tw])) OR bronchitis[mh] OR bronchit*[tw] OR “common cold”[mh] OR “common cold”[tw] OR coryza[tw] OR flu[tw] OR laryngitis[mh] OR laryngit*[tw] OR pharyngitis[mh] OR pharyngit*[tw] OR nasopharyngit*[tw] OR rhinopharyngit*[tw] OR cough[mh] OR cough[tw] OR tonsillitis[mh] OR tonsillit*[tw] OR pneumonia[mh] OR pneumon*[tw] OR bronchopneumon*[tw] OR pleuropneumon*[tw] OR sinusitis[mh] OR sinusit*[tw] OR rhinosinusit*[tw] OR nasosinusit*[tw] OR ((acute[tw] OR viral[tw] OR virus*[tw] OR bacter*[tw]) AND (rhinit*[tw])) OR ((throat*[tw]) AND (infect*[tw] OR inflam*[tw])) |
| **4** | **(coronavirus OR “corona virus” OR coronavirinae OR coronaviridae OR betacoronavirus OR covid19 OR “covid 19” OR nCoV OR “CoV 2” OR CoV2 OR sarscov2 OR 2019nCoV OR “novel CoV” OR “wuhan virus”) OR ((wuhan OR hubei OR huanan) AND (“severe acute respiratory” OR pneumonia) AND (outbreak)) OR “Coronavirus”[Mesh] OR “Coronavirus Infections”[Mesh] OR “COVID-19” [Supplementary Concept] OR “severe acute respiratory syndrome coronavirus 2” [Supplementary Concept] OR “Betacoronavirus”[Mesh]** |
| **5** | “sars virus”[mh] OR "severe acute respiratory syndrome"[mh] OR SARS[tw] OR "severe acute respiratory syndrome"[tw] |
| **6** | MERS[tw] OR "middle east respiratory syndrome coronavirus"[mh] OR "middle east respiratory syndrome”[tw] |
| **7** | “influenza, human"[mh] OR influenza[tw] OR “Influenza A virus”[mh] OR “Influenza B virus”[mh] OR "influenza a virus, h1n1 subtype"[mh] OR H1N1[tw] OR “swine flu”[tw] OR “swine influenza”[tw] OR "Influenza A Virus, H2N2 Subtype"[mh] OR H2N2[tw] OR “asian flu”[tw] OR “asian influenza”[tw] OR "Influenza A Virus, H3N2 Subtype"[mh] OR H3N2[tw] OR “Hong Kong flu”[tw] OR “Hong Kong influenza”[tw] OR "Influenza A Virus, H5N1 Subtype"[mh] OR H5N1[tw] OR "Influenza A Virus, H7N9 Subtype"[mh] OR H7N9[tw] OR “bird flu”[tw] OR “bird influenza”[tw] OR “avian flu”[tw] OR “avian influenza”[tw] |
| **8** | "hemorrhagic fever, ebola"[mh] OR "ebolavirus"[mh] OR ebola[tw] OR ebolavirus[tw] OR ebola-virus[tw] |
| **9** | Tuberculosis[mh] OR tuberculosis[tw] OR phthisis[tw] |
| **10** | #3 OR #4 OR #5 OR #6 OR #7 OR #8 OR #9 |
| **11** | #1 AND #2 AND #10 (Filters: from 1980 - 2020) |

## Suchstring EMBASE via Ovid

| **Nr.** | **Suchterme** |
| --- | --- |
| **1** | newborn.tw. or exp newborn/ or neonat*.tw. or baby.tw. or babies.tw. or exp baby/ or toddler*.tw. or exp toddler/ or kindergartner.tw. or preschool*.tw. or pre-school*.tw. or exp preschool child/ or schoolchild*.tw. or exp school child/ or schoolkid.tw. or pupil.tw. or student.tw. or exp students/ or preteen*.tw. or pre-teen*.tw. or teen*.tw. or adolescen*.tw. or exp adolescent/ or puberty.tw. or exp puberty/ or pubescent*.tw. or youth*.tw. or young*.tw. or juvenile.tw. or exp juvenile/ or infan*.tw. or exp infant/ or child*.tw. or exp child/ or kid.tw. or boy.tw. or exp boy/ or girl.tw. or exp girl/ or pediatric*.tw. or paediatric*.tw. or minor.tw. or underage*.tw. |
| **2** | mask.tw. or exp mask/ or facemask.tw. or exp face mask/ or face pack.tw. or face guard.tw. or face covering.tw. or face coverage.tw. or face shield.tw. or faceshield.tw. or face piece.tw. or facepiece.tw. or visor.tw. or veil.tw. or glasses.tw. or eye protection.tw. or eye guard.tw. or eye shield.tw. or eyewear.tw. or goggles.tw. or exp eye protective device/ or exp protective equipment/ or protective equipment.tw. |
| **3** | exp respiratory tract infection/ or respiratory tract infection.tw. or exp Rhinovirus/ or exp Rhinovirus infection/ or (respiratory and (infect* or illness or symptom* or virus* or syndrome)).tw. or bronchit*.tw. or exp bronchitis/ or common cold.tw. or exp common cold/ or coryza.tw. or flu.tw. or laryngit*.tw. or exp laryngitis/ or pharyngit*.tw. or nasopharyngit*.tw. or rhinopharyngit*.tw. or exp pharyngitis/ or cough.tw. or exp coughing/ or tonsillit*.tw. or exp tonsillitis/ or pneumon*.tw. or bronchopneumon*.tw. or pleuropneumon*.tw. or exp pneumonia/ or sinusit*.tw. or rhinosinusit*.tw. or nasosinusit*.tw. or exp sinusitis/ or ((acute or viral or virus* or bacter*) and rhinit*).tw. or (throat* and (infect* or inflam*)).tw. |
| **4** | **(coronavir* or corona virus* or betacoronavir* or covid19 or covid 19 or nCoV or CoV 2 or CoV2 or sarscov2 or 2019nCoV or 2019 novel coronavirus* or 2019 novel CoV or wuhan virus*).mp. or ((wuhan or hubei or huanan) and (severe acute respiratory or pneumonia*) and outbreak*).mp. or** exp Coronavirus infection/ or exp Coronavirus/ or exp Betacoronavirus/ |
| **5** | exp severe acute respiratory syndrome/ or exp SARS coronavirus/ or SARS.tw. or severe acute respiratory syndrome.tw. |
| **6** | exp Middle East respiratory syndrome coronavirus/ or MERS.tw. or middle east respiratory syndrome.tw. |
| **7** | exp influenza/ or influenza.tw. or exp Influenza virus A/ or exp Influenza A virus/ or exp Influenza virus B/ or exp Influenza B virus/ or exp "influenza A (H1N1)"/ or exp "Influenza A virus (H1N1)"/ or H1N1.tw. or exp swine influenza/ or swine flu.tw. or swine influenza.tw. or exp Influenza virus A H2N2/ or H2N2.tw. or exp Asian influenza/ or asian flu.tw. or asian influenza.tw. or exp "Influenza A virus (H3N2)"/ or H3N2.tw. or exp Hong Kong influenza/ or Hong Kong influenza.tw. or Hong Kong flu.tw. or exp "Influenza A virus (H5N1)"/ or H5N1.tw. or exp "Influenza A virus (H7N9)"/ or H7N9.tw. or exp avian influenza/ or bird flu.tw. or bird influenza.tw. or avian flu.tw. or avian influenza.tw. |
| **8** | exp Ebola hemorrhagic fever/ or exp Ebolavirus/ or ebola.tw. or ebolavirus.tw. or ebola-virus.tw. |
| **9** | exp tuberculosis/ or exp lung tuberculosis/ or tuberculosis.tw. or phthisis.tw. |
| **10** | or/3-9 |
| **11** | 1 and 2 and 10 |

## Suchstring PsycINFO via EBSCOhost

| **Nr.** | **Suchterme** |
| --- | --- |
| **1** | (TX newborn) OR (TX neonat*) OR (DE "Neonatal Period") OR (TX baby) OR (TX toddler) OR (TX kindergartner) OR (DE "Kindergarten Students") OR (TX preschool*) OR (TX pre-school*) OR (DE "Preschool Students") OR (DE "Nursery School Students") OR (TX child*) OR (TX schoolchild*) OR (TX schoolkid) or (TX pupil) OR (TX student) OR (DE "Students") OR (DE "Middle School Students") OR (DE "Primary School Students") OR (DE "Junior High School Students") OR (DE "Intermediate School Students") OR (DE "High School Students") OR (DE "Elementary School Students") OR (TX preteen*) OR (TX pre-teen*) OR (DE "Early Adolescence") OR (TX teen*) OR (TX adolescen*) OR (TX puberty) OR (DE "Puberty") OR (TX pubescent*) OR (TX youth*) OR (TX young*) OR (TX juvenile) OR (TX infan*) OR (TX child*) OR (TX kid) OR (TX boy) OR (DE "Human Males") OR (TX girl) OR (DE "Human Females") OR (TX pediatric*) OR (TX paediatric*) OR (TX minor) OR (TX underage*) |
| **2** | (TX mask) OR (TX facemask) OR (TX face pack) OR (TX face guard) OR (TX face covering) OR (TX face coverage) OR (TX face shield) OR (TX faceshield) OR (TX face piece) OR (TX facepiece) OR (TX visor) OR (TX veil) OR (TX glasses) OR (TX eye protection) OR (TX eye guard) OR (TX eye shield) OR (TX eyewear) OR (TX goggles) OR (TX protective equipment) OR (DE "Personal Protective Equipment") |
| **3** | (DE “Respiratory Tract Disorders”) OR (TX “respiratory tract infection”) OR (TX (respiratory AND (infect* OR illness OR symptom* OR virus* OR syndrome)) OR (TX bronchit*) OR (TX “common cold”) OR (TX coryza) OR (TX flu) OR (TX laryngit*) OR (DE “Laryngeal Disorders”) OR (TX pharyngit*) OR (TX nasopharyngit*) OR (TX rhinopharyngit*) OR (DE “Pharyngeal Disorders”) OR (TX cough) OR (TX tonsillit*) OR (TX pneumon*) OR (TX bronchopneumon*) OR (TX pleuropneumon*) OR (DE “Pneumonia”) OR (TX sinusit*) OR (TX rhinosinusit*) OR (TX nasosinusit*) OR (TX (acute OR viral OR virus* OR bacter*) AND rhinit*) OR (TX (throat* AND (infect* OR inflam*)) |
| **4** | (TX coronavir*) OR (TX corona virus*) OR (TX betacoronavir*) OR (TX “covid 19”) OR (TX nCoV) OR (TX “CoV 2”) OR (TX CoV2) OR (TX **sarscov2) OR (TX 2019nCoV) OR (TX “2019 novel coronavirus*”) OR (TX “2019 novel CoV”) OR (TX “wuhan virus*”) OR (TX (wuhan OR hubei OR huanan) AND (severe acute respiratory OR pneumonia*) AND (outbreak*)) OR (DE “Coronavirus”)** |
| **5** | (DE “Severe Acute Respiratory Syndrome”) OR (TX SARS) OR (TX “Severe Acute Respiratory Syndrome”) |
| **6** | (DE “Middle East Respiratory Syndrome”) OR (TX MERS) OR (TX “Middle East Respiratory Syndrome”) |
| **7** | (DE “Influenza”) OR (TX influenza) OR (TX H1N1) OR (DE “Swine Influenza”) OR (TX swine flu) OR (TX swine influenza) OR (TX H2N2) OR (TX asian flu) OR (TX asian influenza) OR (TX H3N2) OR (TX Hong Kong influenza) OR (TX Hong Kong flu) OR (TX H5N1) OR (TX H7N9) OR (TX bird flu) OR (TX bird influenza) OR (TX avian flu) OR (TX avian influenza) |
| **8** | (TX ebola) OR (TX ebolavirus) OR (TX ebola-virus) |
| **9** | (DE "Tuberculosis") OR (DE "Pulmonary Tuberculosis") OR (TX tuberculosis) OR (TX phthisis) |
| **10** | S3 OR S4 OR S5 OR S6 OR S7 OR S8 OR S9 |
| **11** | S1 AND S2 AND S10 |

## Suchstring PSYNDEX via EBSCOhost

| **Nr.** | **Suchterme** |
| --- | --- |
| **1** | (TX newborn) OR (TX neonat*) OR (DE "Neonatal Period") OR (TX baby) OR (TX toddler) OR (TX kindergartner) OR (DE "Kindergarten Students") OR (TX preschool*) OR (TX pre-school*) OR (DE "Preschool Students") OR (DE "Nursery School Students") OR (TX child*) OR (TX schoolchild*) OR (TX schoolkid) or (TX pupil) OR (TX student) OR (DE "Students") OR (DE "Middle School Students") OR (DE "Primary School Students") OR (DE "Junior High School Students") OR (DE "Intermediate School Students") OR (DE "High School Students") OR (DE "Elementary School Students") OR (TX preteen*) OR (TX pre-teen*) OR (DE "Early Adolescence") OR (TX teen*) OR (TX adolescen*) OR (TX puberty) OR (DE "Puberty") OR (TX pubescent*) OR (TX youth*) OR (TX young*) OR (TX juvenile) OR (TX infan*) OR (TX child*) OR (TX kid) OR (TX boy) OR (DE "Human Males") OR (TX girl) OR (DE "Human Females") OR (TX pediatric*) OR (TX paediatric*) OR (TX minor) OR (TX underage*) |
| **2** | (TX mask) OR (TX facemask) OR (TX face pack) OR (TX face guard) OR (TX face covering) OR (TX face coverage) OR (TX face shield) OR (TX faceshield) OR (TX face piece) OR (TX facepiece) OR (TX visor) OR (TX veil) OR (TX glasses) OR (TX eye protection) OR (TX eye guard) OR (TX eye shield) OR (TX eyewear) OR (TX goggles) OR (TX protective equipment) OR (DE "Personal Protective Equipment") |
| **3** | S1 AND S2 |

## Suchstring PsyArXiv

| **Nr.** | **Suchterme** |
| --- | --- |
| **1** | (newborn OR neonat* OR baby OR toddler OR kindergartner OR preschool* OR pre-school* OR child* OR schoolchild* OR schoolkid OR pupil OR student OR preteen* OR pre-teen* OR teen* OR adolescen* OR puberty OR pubescent* OR youth* OR young* OR juvenile OR infan* OR child* OR kid OR boy OR girl OR pediatric* OR paediatric* OR minor OR underage*)  AND  (mask OR facemask OR “face pack” OR “face guard” OR “face covering” OR “face coverage” OR “face shield” OR faceshield OR “face piece” OR facepiece OR visor OR veil OR glasses OR “eye protection OR “eye guard” OR “eye shield” OR eyewear OR goggles OR “protective equipment”) |

## Suchstring preVIEW: COVID-19

| **Nr.** | **Suchterme** |
| --- | --- |
| **1** | (mask OR facemask OR “face pack” OR “face guard” OR “face covering” OR “face coverage” OR “face shield” OR faceshield OR “face piece” OR facepiece OR visor OR veil OR glasses OR “eye protection OR “eye guard” OR “eye shield” OR eyewear OR goggles OR “protective equipment”) |

## Suchstring Preprints.org

| **Nr.** | **Suchterme** |
| --- | --- |
| **1** | mask OR facemask |

## 2. Für Vorwärtssuche genutzte Studien

Es fand eine Vorwärtssuche der eingeschlossenen Volltexte über ein sogenanntes „Citation Tracking“ in der Web of Science Core Collection statt.

| **Studie** | **Referenz** | **Anzahl der Studien, in denen Referenz zitiert wurde** |
| --- | --- | --- |
| Allison et al., 2010 | Allison MA, Guest-Warnick G, Nelson D, Pavia AT, Srivastava R, Gesteland PH, Rolfs RT, Andersen S, Calame L, Young P, Byington CL. Feasibility of elementary school children's use of hand gel and facemasks during influenza season. Influenza Other Respir Viruses. 2010;4(4):223-9. | *n* = 6 |
| Coniam, 2015 | David Coniam (2005) The Impact of Wearing a Face Mask in a High-Stakes Oral Examination: An Exploratory Post-SARS Study in Hong Kong, Language Assessment Quarterly: An International Journal, 2:4, 235-261. | *n* = 5 |
| Gori, 2021 | Gori M, Schiatti L, Amadeo MB (2021) Masking Emotions: Face Masks Impair How We Read Emotions. Frontiers in psychology 12:669432-669432. 10.3389/fpsyg.2021.669432 | *n* = 1 |
| Kisielinski, 2021 | Kisielinski K, Giboni P, Prescher A et al. (2021) Is a Mask That Covers the Mouth and Nose Free from Undesirable Side Effects in Everyday Use and Free of Potential Hazards? International Journal of Environmental Research and Public Health 18:4344. https://www.mdpi.com/1660-4601/18/8/4344 | *n* = 0 |
| Mickells, 2021 | Mickells GE, Figueroa J, West KW, Wood A, McElhanon BO (2021) Adherence to Masking Requirement During the COVID-19 Pandemic by Early Elementary School Children. Journal of School Health 91:555-561. https://doi.org/10.1111/josh.13033 | *n* = 0 |
| Qin, 2021 | Qin Z, Shi L, Xue Y et al. (2021) Prevalence and Risk Factors Associated With Self-reported Psychological Distress Among Children and Adolescents During the COVID-19 Pandemic in China. JAMA Network Open 4:e2035487-e2035487. 10.1001/jamanetworkopen.2020.35487 | *n* = 0 |
| Rao, 2006 | Rao N. Sars, preschool routines and children's behaviour: Observations from preschools in Hong Kong. Int J Early Child. 2006;38(2):11-22. | *n* = 2 |
| Roberson et al., 2012 | Roberson D, Kikutani M, Döge P, Whitaker L, Majid A. Shades of emotion: what the addition of sunglasses or masks to faces reveals about the development of facial expression processing. Cognition. 2012;125(2):195-206. | *n* = 6 |
| Ruba & Pollak, 2020 | Ruba AL, Pollak SD. Children's emotion inferences from masked faces: Implications for social interactions during COVID-19. PLoS One. 2020;15(12):e0243708. | *n* = 1 |
| Schwarz, 2021 | Schwarz S, Jenetzky E, Krafft H, Maurer T, Martin D (2021) Corona child studies "Co-Ki": first results of a Germany-wide register on mouth and nose covering (mask) in children. Monatsschrift Kinderheilkunde : Organ der Deutschen Gesellschaft fur Kinderheilkunde. 10.1007/s00112-021-01133-91-10. 10.1007/s00112-021-01133-9 | *n* = 2 |
| Sim et al., 2014 | Sim SW, Moey KS, Tan NC. The use of facemasks to prevent respiratory infection: a literature review in the context of the Health Belief Model. Singapore Med J. 2014;55(3):160-7. | *n* = 42 |
| Singh, 2021 | Singh L, Tan A, Quinn PC (2021) Infants recognize words spoken through opaque masks but not through clear masks. Dev Sci. 10.1111/desc.1311710.1111/desc.13117 | *n* = 0 |
| Stajduhar, 2021 | Stajduhar A, Ganel T, Avidan G, Rosenbaum R, Freud E (2021) Face Masks Disrupt Holistic Processing and Face Perception in School-Age Children. psyarxiv. ttps://doi.org/10.31234/osf.io/fygjq. Zugegriffen: 05.08.2021 | *n* = 0 |

## 3. Für Referenzlistensuche genutzte Studien

Es wurden die Referenzlisten der eingeschlossenen Volltexte und von 117 themenrelevanten Artikeln gesichtet.

| **Studie** | **Referenz** | **Anzahl der Studien in der Referenzliste** |
| --- | --- | --- |
| **Eingeschlossene Studien** | | |
| Allison et al., 2010 | Allison MA, Guest-Warnick G, Nelson D, Pavia AT, Srivastava R, Gesteland PH, Rolfs RT, Andersen S, Calame L, Young P, Byington CL. Feasibility of elementary school children's use of hand gel and facemasks during influenza season. Influenza Other Respir Viruses. 2010;4(4):223-9. | *n* = 30 |
| Coniam, 2015 | David Coniam (2005) The Impact of Wearing a Face Mask in a High-Stakes Oral Examination: An Exploratory Post-SARS Study in Hong Kong, Language Assessment Quarterly: An International Journal, 2:4, 235-261. | *n* = 34 |
| Gori, 2021 | Gori M, Schiatti L, Amadeo MB (2021) Masking Emotions: Face Masks Impair How We Read Emotions. Frontiers in psychology 12:669432-669432. 10.3389/fpsyg.2021.669432 | *n* = 65 |
| Kisielinski, 2021 | Kisielinski K, Giboni P, Prescher A et al. (2021) Is a Mask That Covers the Mouth and Nose Free from Undesirable Side Effects in Everyday Use and Free of Potential Hazards? International Journal of Environmental Research and Public Health 18:4344. https://www.mdpi.com/1660-4601/18/8/4344 | *n* = 178 |
| Mickells, 2021 | Mickells GE, Figueroa J, West KW, Wood A, McElhanon BO (2021) Adherence to Masking Requirement During the COVID-19 Pandemic by Early Elementary School Children. Journal of School Health 91:555-561. https://doi.org/10.1111/josh.13033 | *n* = 35 |
| Qin, 2021 | Qin Z, Shi L, Xue Y et al. (2021) Prevalence and Risk Factors Associated With Self-reported Psychological Distress Among Children and Adolescents During the COVID-19 Pandemic in China. JAMA Network Open 4:e2035487-e2035487. 10.1001/jamanetworkopen.2020.35487 | *n* = 33 |
| Rao, 2006 | Rao N. Sars, preschool routines and children's behaviour: Observations from preschools in Hong Kong. Int J Early Child. 2006;38(2):11-22. | *n* = 13 |
| Roberson et al., 2012 | Roberson D, Kikutani M, Döge P, Whitaker L, Majid A. Shades of emotion: what the addition of sunglasses or masks to faces reveals about the development of facial expression processing. Cognition. 2012;125(2):195-206. | *n* = 39 |
| Ruba & Pollak, 2020 | Ruba AL, Pollak SD. Children's emotion inferences from masked faces: Implications for social interactions during COVID-19. PLoS One. 2020;15(12):e0243708. | *n* = 50 |
| Schwarz, 2021 | Schwarz S, Jenetzky E, Krafft H, Maurer T, Martin D (2021) Corona child studies "Co-Ki": first results of a Germany-wide register on mouth and nose covering (mask) in children. Monatsschrift Kinderheilkunde : Organ der Deutschen Gesellschaft fur Kinderheilkunde. 10.1007/s00112-021-01133-91-10. 10.1007/s00112-021-01133-9 | *n* = 22 |
| Sim et al., 2014 | Sim SW, Moey KS, Tan NC. The use of facemasks to prevent respiratory infection: a literature review in the context of the Health Belief Model. Singapore Med J. 2014;55(3):160-7. | *n* = 44 |
| Singh, 2021 | Singh L, Tan A, Quinn PC (2021) Infants recognize words spoken through opaque masks but not through clear masks. Dev Sci. 10.1111/desc.1311710.1111/desc.13117 | *n* = 69 |
| Stajduhar, 2021 | Stajduhar A, Ganel T, Avidan G, Rosenbaum R, Freud E (2021) Face Masks Disrupt Holistic Processing and Face Perception in School-Age Children. psyarxiv. ttps://doi.org/10.31234/osf.io/fygjq. Zugegriffen: 05.08.2021 | *n* = 52 |
| **Themenrelevante Artikel** | | |
| Adhikari et al., 2020 | Adhikari SP ,Pariyar J ,Sapkota K ,Gurung TK, Adhikari SR. Evaluation of Knowledge, Attitude, Practice and Hospital Experience Regarding COVID-19 among Post-partum Mothers at a Tertiary Care Center: A Cross-sectional Study. Kathmandu Univ Med J (KUMJ). 2020 COVID-19 SPECIAL ISSUE;18(70):10-14. | *n* = 17 |
| Ahmad et al., 2017 | Ahmad I, Altaf S, Ahmad HM. Assessment of knowledge, practice and barrier in use of facemask among university students. PJMHS 2017,11(4):1657-8. | *n* = 7 |
| Aiello et al., 2010 | Aiello AE, Coulborn RM, Perez V, Davis BM, Uddin M, Murray GF, Shay DK, Waterman SH, Monto AS. A randomized intervention trial of mask use and hand hygiene to reduce seasonal influenza-like illness and influenza infections among young adults in a university setting. Int J Infect Dis 2010,14(1): e320. | *n* = 0 |
| Aiello et al., 2010 | Aiello AE, Murray GF, Perez V, Coulborn RM, Davis BM, Uddin M, Shay DK, Waterman SH, Monto AS. Mask use, hand hygiene, and seasonal influenza-like illness among young adults: a randomized intervention trial. J Infect Dis. 2010;201(4):491-8. | *n* = 26 |
| Aiello et al., 2012 | Aiello AE, Perez V, Coulborn RM, Davis BM, Uddin M, Monto AS. Facemasks, hand hygiene, and influenza among young adults: a randomized intervention trial. PLoS One. 2012;7(1):e29744. | *n* = 20 |
| Aledort et al., 2007 | Aledort JE, Lurie N, Wasserman J, Bozzette SA. Non-pharmaceutical public health interventions for pandemic influenza: an evaluation of the evidence base. BMC Public Health. 2007 Aug 15;7:208.Ale | *n* = 55 |
| Aronu et al., 2020 | Aronu AE, Chinawa JM, Nduagubam OC, Ossai EN, Chinawa AT, Igwe WC. Maternal perception of masking in children as a preventive strategy for COVID-19 in Nigeria: A multicentre study. PLoS One. 2020 Nov 19;15(11):e0242650. | *n* = 31 |
| Atcherson et al., 2017 | Atcherson SR, Mendel LL, Baltimore WJ, Patro C, Lee S, Pousson M, Spann MJ. The Effect of Conventional and Transparent Surgical Masks on Speech Understanding in Individuals with and without Hearing Loss. J Am Acad Audiol. 2017 Jan;28(1):58-67. | *n* = 23 |
| Barasheed et al., 2016 | Barasheed O, Alfelali M, Mushta S, Bokhary H, Alshehri J, Attar AA, Booy R, Rashid H. Uptake and effectiveness of facemask against respiratory infections at mass gatherings: a systematic review. Int J Infect Dis. 2016 Jun;47:105-11. | *n* = 75 |
| Barcelo & Sheen, 2020 | Barceló J, Sheen GC. Voluntary adoption of social welfare-enhancing behavior: Mask-wearing in Spain during the COVID-19 outbreak. PLoS One. 2020 Dec 1;15(12):e0242764. | *n* = 39 |
| Beck et al., 2004 | Beck M, Antle BJ, Berlin D, Granger M, Meighan K, Neilson BJ, Shama W, Westland J, Kaufman M. Wearing masks in a pediatric hospital: developing practical guidelines. Can J Public Health. 2004 Jul-Aug;95(4):256-7. | *n* = 9 |
| Betsch et al., 2020 | Betsch C, Korn L, Sprengholz P, Felgendreff L, Eitze S, Schmid P, Böhm R. Social and behavioral consequences of mask policies during the COVID-19 pandemic. Proc Natl Acad Sci U S A. 2020 Sep 8;117(36):21851-21853. | *n* = 16 |
| Beuvelet et al., 2017 | Beuvelet M, Masson C, Gantz D, Levet S, Allenet B, Reine Mallaret M, Landelle C. Healthcare workers’ perception towards the systematic use of mask during a seasonal influenza outbreak in a French University Hospital: a descriptive study. Antimicrobial Resistance and Infection Control 2017, 6(Suppl 3):52 | *n* = 0 |
| Bin-Reza et al., 2012 | Bin-Reza F, Lopez Chavarrias V, Nicoll A, Chamberland ME. The use of masks and respirators to prevent transmission of influenza: a systematic review of the scientific evidence. Influenza Other Respir Viruses. 2012 Jul;6(4):257-67. | *n* = 35 |
| Bottalico et al., 2020 | Bottalico P, Murgia S, Puglisi GE, Astolfi A, Kirk KI. Effect of masks on speech intelligibility in auralized classrooms. J Acoust Soc Am. 2020 Nov;148(5):2878. | *n* = 21 |
| Bressington et al., 2020 | Bressington DT, Cheung TCC, Lam SC, Suen LKP, Fong TKH, Ho HSW, Xiang YT. Association Between Depression, Health Beliefs, and Face Mask Use During the COVID-19 Pandemic. Front Psychiatry. 2020 Oct 22;11:571179. | *n* = 47 |
| Burgess et al., 2012 | Burgess A, Horii M. Risk, ritual and health responsibilisation: Japan's 'safety blanket' of surgical face mask-wearing. Sociol Health Illn. 2012 Nov;34(8):1184-98. | *n* = 54 |
| Canini et al., 2010 | Canini L, Andréoletti L, Ferrari P, D'Angelo R, Blanchon T, Lemaitre M, Filleul L, Ferry JP, Desmaizieres M, Smadja S, Valleron AJ, Carrat F. Surgical mask to prevent influenza transmission in households: a cluster randomized trial. PLoS One. 2010 Nov 17;5(11):e13998. | *n* = 18 |
| Carbon, 2020 | Carbon CC. Wearing Face Masks Strongly Confuses Counterparts in Reading Emotions. Front Psychol. 2020 Sep 25;11:566886. doi: 10.3389/fpsyg.2020.566886. | *n* = 42 |
| Carbon, 2020 | Carbon, Claus-Christian, The Psychology of Wearing Face Masks in Times of the COVID-19 Pandemic (April 24, 2020). Available at SSRN: https://ssrn.com/abstract=3584834 or http://dx.doi.org/10.2139/ssrn.3584834 | *n* = 20 |
| Chen et al., 2020 | Chen X, Ran L, Liu Q, Hu Q, Du X, Tan X. Hand Hygiene, Mask-Wearing Behaviors and Its Associated Factors during the COVID-19 Epidemic: A Cross-Sectional Study among Primary School Students in Wuhan, China. Int J Environ Res Public Health. 2020 Apr 22;17(8):2893. | *n* = 21 |
| Chodosh et al., 2020 | Chodosh J, Weinstein BE, Blustein J. Face masks can be devastating for people with hearing loss. BMJ. 2020 Jul 9;370:m2683. | *n* = 16 |
| Cook, 2020 | Cook TM. Personal protective equipment during the coronavirus disease (COVID) 2019 pandemic - a narrative review. Anaesthesia. 2020 Jul;75(7):920-927. | *n* = 42 |
| Chu et al., 2020 | Chu DK, Akl EA, Duda S, Solo K, Yaacoub S, Schünemann HJ; COVID-19 Systematic Urgent Review Group Effort (SURGE) study authors. Physical distancing, face masks, and eye protection to prevent person-to-person transmission of SARS-CoV-2 and COVID-19: a systematic review and meta-analysis. Lancet. 2020 Jun 27;395(10242):1973-1987. | *n* = 87 |
| Corey et al., 2020 | Corey RM, Jones U, Singer AC. Acoustic effects of medical, cloth, and transparent face masks on speech signals. J Acoust Soc Am. 2020 Oct;148(4):2371. | *n* = 10 |
| Coronado et al., 2020 | Coronado F, Blough S, Bergeron D, Proia K, Sauber-Schatz E, Beltran M, Rau KT, McMichael A, Fortin T, Lackey M, Rohs J, Sparrow T, Baldwin G. Implementing Mitigation Strategies in Early Care and Education Settings for Prevention of SARS-CoV-2 Transmission - Eight States, September-October 2020. MMWR Morb Mortal Wkly Rep. 2020 Dec 11;69(49):1868-1872. | *n* = 9 |
| Cotrin et al., 2020 | Cotrin P, Bahls AC, da Silva DO, Girão VMP, Pinzan-Vercelino CRM, de Oliveira RCG, Oliveira RC, Carvalho MDB, Pelloso SM, Valarelli FP, Freitas KMS. The Use of Facemasks During the COVID-19 Pandemic by the Brazilian Population. J Multidiscip Healthc. 2020 Oct 19;13:1169-1178. | *n* = 43 |
| Cowling et al., 2008 | Cowling BJ, Fung RO, Cheng CK, Fang VJ, Chan KH, Seto WH, Yung R, Chiu B, Lee P, Uyeki TM, Houck PM, Peiris JS, Leung GM. Preliminary findings of a randomized trial of non-pharmaceutical interventions to prevent influenza transmission in households. PLoS One. 2008 May 7;3(5):e2101. | *n* = 40 |
| Cowling, 2010 | Cowling BJ, Zhou Y, Ip DK, Leung GM, Aiello AE. Face masks to prevent transmission of influenza virus: a systematic review. Epidemiol Infect. 2010 Apr;138(4):449-56. | *n* = 42 |
| Crowe, 2020 | Crowe AL. Communication skills with children in paediatric anaesthesia: challenges while wearing a face mask. BMJ Paediatr Open. 2020 Oct 27;4(1):e000846. | *n* = 9 |
| Del Valle et al., 2010 | Del Valle SY, Tellier R, Settles GS, Tang JW. Can we reduce the spread of influenza in schools with face masks? Am J Infect Control. 2010;38(9):676-7. | *n* = 5 |
| Dias et al., 2020 | Dias JV, Contreiras M, Oom P. SARS-CoV-2 Pandemic: Should Children Wear Masks? Acta Med Port 2020;33(10):711. | *n* = 5 |
| Ehrhardt et al., 2020 | Ehrhardt J, Ekinci A, Krehl H, Meincke M, Finci I, Klein J, Geisel B, Wagner-Wiening C, Eichner M, Brockmann SO. Transmission of SARS-CoV-2 in children aged 0 to 19 years in childcare facilities and schools after their reopening in May 2020, Baden-Württemberg, Germany. Euro Surveill. 2020 Sep;25(36):2001587. | *n* = 8 |
| Ersin & Kartal, 2020 | Ersin F, Kartal M. The determination of the perceived stress levels and health-protective behaviors of nursing students during the COVID-19 pandemic. Perspect Psychiatr Care. 2020:10.1111/ppc.12636. | *n* = 29 |
| Esposito & Principi, 2020 | Esposito S, Principi N. To mask or not to mask children to overcome COVID-19. Eur J Pediatr. 2020;179(8):1267-1270. | *n* = 20 |
| Esposito & Principi, 2020 | Esposito S, Principi N. Mask-wearing in pediatric age. Eur J Pediatr. 2020;179(8):1341-1342. | *n* = 7 |
| Ferng et al., 2011 | Ferng YH, Wong-McLoughlin J, Barrett A, Currie L, Larson E. Barriers to mask wearing for influenza-like illnesses among urban Hispanic households. Public Health Nurs. 2011;28(1):13-23. | *n* = 29 |
| Fischer et al., 2012 | Fischer AH, Gillebaart M, Rotteveel M, Becker D, Vliek M. Veiled Emotions : The Effect of Covered Faces on Emotion Perception and Attitudes. Social Psychological and Personality Science 2012 3. | *n* = 26 |
| Fisher et al., 2020 | Fisher KA, Barile JP, Guerin RJ, Vanden Esschert KL, Jeffers A, Tian LH, Garcia-Williams A, Gurbaxani B, Thompson WW, Prue CE. Factors Associated with Cloth Face Covering Use Among Adults During the COVID-19 Pandemic - United States, April and May 2020. MMWR Morb Mortal Wkly Rep. 2020 Jul 17;69(28):933-937. | *n* = 9 |
| Forgie et al., 2009 | Forgie SE, Reitsma J, Spady D, Wright B, Stobart K. The "fear factor" for surgical masks and face shields, as perceived by children and their parents. Pediatrics. 2009 Oct;124(4):e777-81. | *n* = 10 |
| Gagnon et al., 2014 | Gagnon M, Gosselin P, Maassarani R. Children's ability to recognize emotions from partial and complete facial expressions. J Genet Psychol. 2014 Sep-Dec;175(5-6):416-30. | *n* = 32 |
| Gilbert et al., 2020 | Gilbert LK, Strine TW, Szucs LE, Crawford TN, Parks SE, Barradas DT, Njai R, Ko JY. Racial and Ethnic Differences in Parental Attitudes and Concerns About School Reopening During the COVID-19 Pandemic - United States, July 2020. MMWR Morb Mortal Wkly Rep. 2020 Dec 11;69(49):1848-1852. | *n* = 10 |
| Goh et al., 2019 | Goh DYT, Mun MW, Lee WLJ, Teoh OH, Rajgor DD. A randomised clinical trial to evaluate the safety, fit, comfort of a novel N95 mask in children. Sci Rep. 2019 Dec 12;9(1):18952. | *n* = 42 |
| Goldin et al., 2020 | Goldin A, Weinstein BE, Shiman N. How do medical masks degrade speech perception? *Hearing Review*. 2020;27(5):8-9. | *n* = 3 |
| Green, 2021 | Green J, Staff L, Bromley P, Jones L, Petty J. The implications of face masks for babies and families during the COVID-19 pandemic: A discussion paper. J Neonatal Nurs. 2021;27(1):21-25. | *n* = 48 |
| Guarnera et al., 2015 | Guarnera M, Hichy Z, Cascio MI, Carrubba S. Facial Expressions and Ability to Recognize Emotions From Eyes or Mouth in Children. Eur J Psychol. 2015 May 29;11(2):183-96. | *n* = 59 |
| Gupta et al., 2020 | Madhu Gupta, Khushi Gupta, Sarika Gupta. The use of facemasks by the general population to prevent transmission of Covid 19 infection: A systematic review. May 2020. DOI: 10.1101/2020.05.01.20087064 | *n* = 20 |
| Hampton et al., 2020 | Hampton T, Crunkhorn R, Lowe N, Bhat J, Hogg E, Afifi W, De S, Street I, Sharma R, Krishnan M, Clarke R, Dasgupta S, Ratnayake S, Sharma S. The negative impact of wearing personal protective equipment on communication during coronavirus disease 2019. J Laryngol Otol. 2020 Jul;134(7):577-581. | *n* = 30 |
| Heald et al., 2020 | Heald AH, Stedman M, Tian Z, Wu P, Fryer AA. Modelling the impact of the mandatory use of face coverings on public transport and in retail outlets in the UK on COVID-19-related infections, hospital admissions and mortality. Int J Clin Pract. 2020 Oct 18:e13768. | *n* = 39 |
| Howard et al., 2021 | Howard J, Huang A, Li Z, Tufekci Z, Zdimal V, van der Westhuizen HM, von Delft A, Price A, Fridman L, Tang LH, Tang V, Watson GL, Bax CE, Shaikh R, Questier F, Hernandez D, Chu LF, Ramirez CM, Rimoin AW. An evidence review of face masks against COVID-19. Proc Natl Acad Sci U S A. 2021 Jan 26;118(4):e2014564118. | *n* = 141 |
| Huppertz et al., 2020 | Huppertz HI, Berner R, Schepker R, Kopp M, Oberle A, Fischbach T, Rodeck B, Knuf M, Keller M, Simon A, Hübner J. Verwendung von Masken bei Kindern zur Verhinderung der Infektion mit SARS-CoV-2: Stellungnahme der Deutschen Gesellschaft für Pädiatrische Infektiologie (DGPI), des Berufsverbandes der Kinder- und Jugendärzte (bvkj e. V.), der Deutschen Gesellschaft für Kinder- und Jugendmedizin (DGKJ), der Gesellschaft für Pädiatrische Pneumologie (GPP), der Deutschen Gesellschaft für Sozialpädiatrie und Jugendmedizin (DGSPJ), der Süddeutschen Gesellschaft für Kinder- und Jugendmedizin (SGKJ) und der Deutschen Gesellschaft für Kinder- und Jugendpsychiatrie, Psychosomatik und Psychotherapie (DGKJP) [Use of masks by children to prevent infection with SARS-CoV-2]. Monatsschr Kinderheilkd. 2020 Dec 18:1-5. | *n* = 24 |
| Javid et al., 2020 | Javid B, Weekes MP, Matheson NJ. Covid-19: should the public wear face masks? BMJ. 2020 Apr 9;369:m1442. | *n* = 13 |
| Jefferson et al., 2009 | Jefferson T, Del Mar C, Dooley L, Ferroni E, Al-Ansary LA, Bawazeer GA, van Driel ML, Foxlee R, Rivetti A. Physical interventions to interrupt or reduce the spread of respiratory viruses: systematic review. BMJ. 2009 Sep 21;339:b3675. | *n* = 24 |
| Jin et al., 2020 | Jin K, Min J, Jin X. Re: Esposito et al.: To mask or not to mask children to overcome COVID-19. Eur J Pediatr. 2020;179(8):1339-1340. | *n* = 4 |
| Kenyon, 2020 | Kenyon C. Widespread use of face masks in public may slow the spread of SARS CoV-2: an ecological study. doi: https://doi.org/10.1101/2020.03.31.20048652 | *n* = 21 |
| Larson et al., 2010 | Larson EL, Ferng YH, Wong-McLoughlin J, Wang S, Haber M, Morse SS. Impact of non-pharmaceutical interventions on URIs and influenza in crowded, urban households. Public Health Rep. 2010 Mar-Apr;125(2):178-91. | *n* = 67 |
| Lau et al., 2007 | Lau JT, Kim JH, Tsui HY, Griffiths S. Anticipated and current preventive behaviors in response to an anticipated human-to-human H5N1 epidemic in the Hong Kong Chinese general population. BMC Infect Dis. 2007 Mar 15;7:18. | *n* = 30 |
| Lau et al., 2010 | Lau JT, Griffiths S, Choi KC, Lin C. Prevalence of preventive behaviors and associated factors during early phase of the H1N1 influenza epidemic. Am J Infect Control. 2010 Jun;38(5):374-80. | *n* = 16 |
| Li et al., 2005 | Li Y, Tokura H, Guo YP, Wong AS, Wong T, Chung J, Newton E. Effects of wearing N95 and surgical facemasks on heart rate, thermal stress and subjective sensations. Int Arch Occup Environ Health. 2005 Jul;78(6):501-9. | *n* = 12 |
| Lopez et al., 2020 | Lopez L, Nguyen T, Weber G, Kleimola K, Bereda M, Liu Y, Accorsi EK, Skates SJ, Santa Maria JP, Smith KR, Kalinich M. Seroprevalence of anti-SARS-CoV-2 IgG Antibodies in the Staff of a Public School System in the Midwestern United States. medRxiv [Preprint]. 2020 Oct 27:2020.10.23.20218651. | *n* = 20 |
| Llamas et al., 2008 | Llamas, C, Harrison, P, Donnelly, D & Watt, D 2008, 'Effects of different types of face coverings on speech acoustics and intelligibility', *York Papers in Linguistics Series 2*, no. 9, pp. 80-104. | *n* = 38 |
| MacIntyre et al., 2009 | MacIntyre CR, Cauchemez S, Dwyer DE, Seale H, Cheung P, Browne G, Fasher M, Wood J, Gao Z, Booy R, Ferguson N. Face mask use and control of respiratory virus transmission in households. Emerg Infect Dis. 2009 Feb;15(2):233-41. | *n* = 32 |
| MacIntyre et al., 2015 | MacIntyre CR, Chughtai AA. Facemasks for the prevention of infection in healthcare and community settings. BMJ. 2015 Apr 9;350:h694. | *n* = 155 |
| Marler & Ditton, 2021 | Marler H, Ditton A. "I'm smiling back at you": Exploring the impact of mask wearing on communication in healthcare. Int J Lang Commun Disord. 2021 Jan;56(1):205-214. | *n* = 44 |
| Marta et al., 2020 | Calbi Marta, Langiulli Nunzio, Ferroni Francesca, Montalti Martina, Kolesnikov Anna, Gallese Vittorio, Umiltà Maria Alessandra. I see how you feel: facial expressions’ recognition and distancing in the time of COVID-19. 2020. 10.21203/rs.3.rs-107145/v1 | *n* = 56 |
| Matuschek et al., 2020 | Matuschek C, Moll F, Fangerau H, Fischer JC, Zänker K, van Griensven M, Schneider M, Kindgen-Milles D, Knoefel WT, Lichtenberg A, Tamaskovics B, Djiepmo-Njanang FJ, Budach W, Corradini S, Häussinger D, Feldt T, Jensen B, Pelka R, Orth K, Peiper M, Grebe O, Maas K, Gerber PA, Pedoto A, Bölke E, Haussmann J. Face masks: benefits and risks during the COVID-19 crisis. Eur J Med Res. 2020 Aug 12;25(1):32. | *n* = 20 |
| Matusiak et al., 2020 | Matusiak Ł, Szepietowska M, Krajewski P, Białynicki-Birula R, Szepietowski J. Face masks use during the COVID-19 pandemic: Differences in attitudes and practices between medical and non-medical students. A survey of 2256 students in Poland. Adv Clin Exp Med. 2020;29(10):1201-1203. | *n* = 9 |
| Matusiak et al., 2020 | Matusiak Ł, Szepietowska M, Krajewski P, Białynicki-Birula R, Szepietowski JC. Inconveniences due to the use of face masks during the COVID-19 pandemic: A survey study of 876 young people. Dermatol Ther. 2020;33(4):e13567. | *n* = 9 |
| Matusiak et al., 2020 | Matusiak Ł, Szepietowska M, Krajewski PK, Białynicki-Birula R, Szepietowski JC. The use of face masks during the COVID-19 pandemic in Poland: A survey study of 2315 young adults. Dermatol Ther. 2020;33(6):e13909. | *n* = 11 |
| Maynard et al., 2016 | Maynard SL, Kao R, Craig DG. Impact of personal protective equipment on clinical output and perceived exertion. J R Army Med Corps. 2016 Jun;162(3):180-3. doi: 10.1136/jramc-2015-000541. | *n* = 8 |
| Meilicke et al., 2013 | Meilicke G, Riedmann K, Biederbick W, Müller U, Wierer T, Bartels C. Hygiene perception changes during the influenza A H1N1 pandemic in Germany: incorporating the results of two cross-sectional telephone surveys 2008-2009. BMC Public Health. 2013 Oct 16;13:959. | *n* = 28 |
| Mendel et al., 2008 | Mendel LL, Gardino JA, Atcherson SR. Speech understanding using surgical masks: a problem in health care? J Am Acad Audiol. 2008 Oct;19(9):686-95. | *n* = 29 |
| Mehta et al., 2020 | Mehta UM, Venkatasubramanian G, Chandra PS. The "mind" behind the "mask": Assessing mental states and creating therapeutic alliance amidst COVID-19. Schizophr Res. 2020 Aug;222:503-504. | *n* = 11 |
| Mitze et al., 2020 | Mitze T, Kosfeld R, Rode J, Wälde K. Face masks considerably reduce COVID-19 cases in Germany. Proc Natl Acad Sci U S A. 2020 Dec 22;117(51):32293-32301. | *n* = 23 |
| Moran et al., 2016 | Moran KR, Del Valle SY. A Meta-Analysis of the Association between Gender and Protective Behaviors in Response to Respiratory Epidemics and Pandemics. PLoS One. 2016 Oct 21;11(10):e0164541. | *n* = 133 |
| Morishima & Kishida, 2018 | Morishima M, Kishida K. Understanding attitudes toward hygiene mask use in Japanese daily life by using a repeated cross-sectional survey. Work. 2018;61(2):303-311. | *n* = 21 |
| Muzzi et al., 2021 | Muzzi E, Chermaz C, Castro V, Zaninoni M, Saksida A, Orzan E. Short report on the effects of SARS-CoV-2 face protective equipment on verbal communication. Eur Arch Otorhinolaryngol. 2021 Jan 3:1–6. doi: 10.1007/s00405-020-06535-1. | *n* = 10 |
| Nobrega et al., 2020 | Nobrega M, Opice R, Lauletta MM, Nobrega CA. How face masks can affect school performance. Int J Pediatr Otorhinolaryngol. 2020 Nov;138:110328. | *n* = 9 |
| Palmiero et al., 2016 | Palmiero AJ, Symons D, Morgan JW 3rd, Shaffer RE. Speech intelligibility assessment of protective facemasks and air-purifying respirators. J Occup Environ Hyg. 2016 Dec;13(12):960-968. | *n* = 24 |
| Pereira-Ávila et al.,2020 | Pereira-Ávila FMV, Lam SC, Góes FGB, Gir E, Pereira-Caldeira NMV, Teles SA, Caetano KAA, Goulart MCEL, Bazilio TR, Silva ACOE. Factors associated with the use and reuse of face masks among Brazilian individuals during the COVID-19 pandemic. Rev Lat Am Enfermagem. 2020 Sep 7;28:e3360. | *n* = 19 |
| Pfattheicher et al., 2020 | Pfattheicher S, Nockur L, Böhm R, Sassenrath C, Petersen MB. The Emotional Path to Action: Empathy Promotes Physical Distancing and Wearing of Face Masks During the COVID-19 Pandemic. Psychol Sci. 2020 Nov;31(11):1363-1373. | *n* = 37 |
| Rabinowicz et al., 2020 | Rabinowicz S, Leshem E, Pessach IM. COVID-19 in the Pediatric Population-Review and Current Evidence. Curr Infect Dis Rep. 2020;22(11):29. | *n* = 107 |
| Radonovich et al., 2010 | Radonovich LJ Jr, Yanke R, Cheng J, Bender B. Diminished speech intelligibility associated with certain types of respirators worn by healthcare workers. J Occup Environ Hyg. 2010 Jan;7(1):63-70. | *n* = 35 |
| Ravens-Sieberer et al., 2020 | Ravens-Sieberer U, Kaman A, Otto C, Adedeji A, Devine J, Erhart M, Napp AK, Becker M, Blanck-Stellmacher U, Löffler C, Schlack R, Hurrelmann K. Mental Health and Quality of Life in Children and Adolescents During the COVID-19 Pandemic-Results of the Copsy Study. Dtsch Arztebl Int. 2020 Oct 20;117(48):828-829. | *n* = 5 |
| Ribeiro et al., 2020 | Ribeiro VV, Dassie-Leite AP, Pereira EC, Santos ADN, Martins P, Irineu RA. Effect of Wearing a Face Mask on Vocal Self-Perception during a Pandemic. J Voice. 2020 Sep 30:S0892-1997(20)30356-8. | *n* = 33 |
| Roberge, 2011 | Roberge R. Facemask use by children during infectious disease outbreaks. Biosecur Bioterror. 2011;9(3):225-31. | *n* = 68 |
| Saeidi et al., 2016 | Saeidi, R., Huhtakallio, I., & Alku, P. (2016). Analysis of Face Mask Effect on Speaker Recognition. INTERSPEECH. | *n* = 19 |
| Saunders et al., 2020 | Saunders GH, Jackson IR, Visram AS. Impacts of face coverings on communication: an indirect impact of COVID-19. Int J Audiol. 2020 Nov 27:1-12. | *n* = 48 |
| Scheid et al., 2020 | Scheid JL, Lupien SP, Ford GS, West SL. Commentary: Physiological and Psychological Impact of Face Mask Usage during the COVID-19 Pandemic. Int J Environ Res Public Health. 2020;17(18):6655. | *n* = 64 |
| Sergi & Leung, 2020 | Sergi CM, Leung AKC. The facemask in public and healthcare workers: a need, not a belief. Public Health. 2020 Jun;183:67-68. | *n* = 15 |
| Schlög & Jones, 2020 | Schlögl M, A Jones C. Maintaining Our Humanity Through the Mask: Mindful Communication During COVID-19. J Am Geriatr Soc. 2020 May;68(5):E12-E13. | *n* = 7 |
| Seale et al., 2020 | Seale H, Dyer CEF, Abdi I, Rahman KM, Sun Y, Qureshi MO, Dowell-Day A, Sward J, Islam MS. Improving the impact of non-pharmaceutical interventions during COVID-19: examining the factors that influence engagement and the impact on individuals. BMC Infect Dis. 2020 Aug 17;20(1):607. | *n* = 79 |
| Sengul et al., 2020 | Sengul H, Bulut A, Coskun SN. Psychological effect of covid-19 pandemic on university students in turkey. Bayrakol 2020. | *n* = 27 |
| Shack et al., 2020 | Shack AR, Arkush L, Reingold S, Weiser G. Masked paediatricians during the COVID-19 pandemic and communication with children. J Paediatr Child Health. 2020;56(9):1475-1476. | *n* = 3 |
| Simmerman et al., 2011 | Simmerman JM, Suntarattiwong P, Levy J, Jarman RG, Kaewchana S, Gibbons RV, Cowling BJ, Sanasuttipun W, Maloney SA, Uyeki TM, Kamimoto L, Chotipitayasunondh T. Findings from a household randomized controlled trial of hand washing and face masks to reduce influenza transmission in Bangkok, Thailand. Influenza Other Respir Viruses. 2011 Jul;5(4):256-67. | *n* = 29 |
| Siu, 2016 | Siu JY. Qualitative study on the shifting sociocultural meanings of the facemask in Hong Kong since the severe acute respiratory syndrome (SARS) outbreak: implications for infection control in the post-SARS era. Int J Equity Health. 2016 May 4;15:73. | *n* = 28 |
| Smart et al., 2020 | Smart NR, Horwell CJ, Smart TS, Galea KS. Assessment of the Wearability of Facemasks against Air Pollution in Primary School-Aged Children in London. Int J Environ Res Public Health. 2020 Jun 2;17(11):3935. | *n* = 29 |
| Smith et al., 2021 | Smith LE, Duffy B, Moxham-Hall V, Strang L, Wessely S, Rubin GJ. Anger and confrontation during the COVID-19 pandemic: a national cross-sectional survey in the UK. J R Soc Med. 2021;114(2):77-90. | *n* = 59 |
| Spitzer 2020 | Spitzer M. Masked education? The benefits and burdens of wearing face masks in schools during the current Corona pandemic. Trends Neurosci Educ. 2020;20:100138. | *n* = 76 |
| Stebbins et al., 2009 | Stebbins S, Downs JS, Vukotich CJ Jr. Using nonpharmaceutical interventions to prevent influenza transmission in elementary school children: parent and teacher perspectives. J Public Health Manag Pract. 2009;15(2):112-7. | *n* = 15 |
| Suess et al., 2011 | Suess T, Remschmidt C, Schink S, Luchtenberg M, Haas W, Krause G, Buchholz U. Facemasks and intensified hand hygiene in a German household trial during the 2009/2010 influenza A(H1N1) pandemic: adherence and tolerability in children and adults. Epidemiol Infect. 2011;139(12):1895-901. | *n* = 14 |
| Suess et al., 2012 | Suess T, Remschmidt C, Schink SB, Schweiger B, Nitsche A, Schroeder K, Doellinger J, Milde J, Haas W, Koehler I, Krause G, Buchholz U. The role of facemasks and hand hygiene in the prevention of influenza transmission in households: results from a cluster randomised trial; Berlin, Germany, 2009-2011. BMC Infect Dis. 2012;12:26. | *n* = 31 |
| Szczesniak et al., 2020 | Szczesniak D, Ciulkowicz M, Maciaszek J, Misiak B, Luc D, Wieczorek T, Witecka KF, Rymaszewska J. Psychopathological responses and face mask restrictions during the COVID-19 outbreak: Results from a nationwide survey. Brain Behav Immun. 2020 Jul;87:161-162. | *n* = 10 |
| Tang & Wong, 2004 | Tang CS, Wong CY. Factors influencing the wearing of facemasks to prevent the severe acute respiratory syndrome among adult Chinese in Hong Kong. Prev Med. 2004 Dec;39(6):1187-93. | *n* = 40 |
| Taylor et al., 2009 | Taylor M, Raphael B, Barr M, Agho K, Stevens G, Jorm L. Public health measures during an anticipated influenza pandemic: Factors influencing willingness to comply. Risk Manag Healthc Policy. 2009;2:9-20. | *n* = 23 |
| Teasdale et al., 2014 | Teasdale E, Santer M, Geraghty AW, Little P, Yardley L. Public perceptions of non-pharmaceutical interventions for reducing transmission of respiratory infection: systematic review and synthesis of qualitative studies. BMC Public Health. 2014;14:589. | *n* = 64 |
| Tooher et al., 2013 | Tooher R, Collins JE, Street JM, Braunack-Mayer A, Marshall H. Community knowledge, behaviours and attitudes about the 2009 H1N1 Influenza pandemic: a systematic review. Influenza Other Respir Viruses. 2013 Nov;7(6):1316-27. | *n* = 30 |
| Truong et al. 2006 | Truong J, Jain S, Tan J, Keegan D, Matsui D, Rieder MJ. Young children's perceptions of physicians wearing standard precautions versus customary attire. Pediatr Emerg Care. 2006;22(1):13-7. | *n* = 9 |
| Uchida et al., 2016 | Uchida M, Kaneko M, Hidaka Y, Yamamoto H, Honda T, Takeuchi S, Saito M, Kawa S. Effectiveness of vaccination and wearing masks on seasonal influenza in Matsumoto City, Japan, in the 2014/2015 season: An observational study among all elementary schoolchildren. Prev Med Rep. 2016;5:86-91. | *n* = 27 |
| van der Sande et al., 2008 | van der Sande M, Teunis P, Sabel R. Professional and home-made face masks reduce exposure to respiratory infections among the general population. PLoS One. 2008 Jul 9;3(7):e2618. | *n* = 16 |
| Wan-Arfah et al., 2012 | Wan-Arfah N, Norsa'adah B, Naing NN, Zaliha I, Azriani AR, Nik-Rosmawati NH, Mohamed-Rusli A. Knowledge, attitudes and practices on influenza a (H1N1) among Kelantanese schoolchildren. Southeast Asian J Trop Med Public Health. 2012 Nov;43(6):1489-501. | *n* = 19 |
| WHO, 2006 | World Health Organization Writing Group, Bell D, Nicoll A, Fukuda K, Horby P, Monto A, Hayden F, Wylks C, Sanders L, van Tam J. Non-pharmaceutical interventions for pandemic influenza, national and community measures. Emerg Infect Dis. 2006 Jan;12(1):88-94. | *n* = 35 |
| WHO, 2020 | World Health Organization. (‎2020)‎. Mask use in the context of COVID-19: interim guidance, 1 December 2020. World Health Organization. https://apps.who.int/iris/handle/10665/337199. License: CC BY-NC-SA 3.0 IGO | *n* = 171 |
| WHO, 2020 | WHO. Advice on the use of masks for children in the community in the context of COVID-19. 2020 | *n* = 62 |
| Wong & Tang, 2005 | Wong CY, Tang CS. Practice of habitual and volitional health behaviors to prevent severe acute respiratory syndrome among Chinese adolescents in Hong Kong. J Adolesc Health. 2005 Mar;36(3):193-200. | *n* = 40 |
| Wong et al., 2013 | Wong CK, Yip BH, Mercer S, Griffiths S, Kung K, Wong MC, Chor J, Wong SY. Effect of facemasks on empathy and relational continuity: a randomised controlled trial in primary care. BMC Fam Pract. 2013 Dec 24;14:200 | *n* = 37 |
| Xiao et al., 2020 | Xiao J, Shiu EYC, Gao H, Wong JY, Fong MW, Ryu S, Cowling BJ. Nonpharmaceutical Measures for Pandemic Influenza in Nonhealthcare Settings-Personal Protective and Environmental Measures. Emerg Infect Dis. 2020 May;26(5):967-975. | *n* = 50 |

## 4. Ausgeschlossene Volltexte mit Ausschlussgrund

| **Studie** | **Referenz** | **Ausschlussgrund** |
| --- | --- | --- |
| Anonym, 2004 | Centers for Disease Control and Prevention (CDC). Experiences with influenza-like illness and attitudes regarding influenza prevention--United States, 2003-04 influenza season. MMWR Morb Mortal Wkly Rep. 2004;53(49):1156-8. | **Outcome:** Bereitschaft zum Maskentragen |
| Adhikari et al., 2020 | Adhikari SP ,Pariyar J ,Sapkota K ,Gurung TK, Adhikari SR. Evaluation of Knowledge, Attitude, Practice and Hospital Experience Regarding COVID-19 among Post-partum Mothers at a Tertiary Care Center: A Cross-sectional Study. Kathmandu Univ Med J (KUMJ). 2020 COVID-19 SPECIAL ISSUE;18(70):10-14. | **Outcome:** Prävalenz des Maskentragens |
| Ahmad et al., 2017 | Ahmad I, Altaf S, Ahmad HM. Assessment of knowledge, practice and barrier in use of facemask among university students. PJMHS 2017,11(4):1657-8. | **Population:** Studierende |
| Aiello et al., 2010 | Aiello AE, Coulborn RM, Perez V, Davis BM, Uddin M, Murray GF, Shay DK, Waterman SH, Monto AS. A randomized intervention trial of mask use and hand hygiene to reduce seasonal influenza-like illness and influenza infections among young adults in a university setting. Int J Infect Dis 2010,14(1): e320. | **Population:** Studierende |
| Aiello et al., 2010 | Aiello AE, Murray GF, Perez V, Coulborn RM, Davis BM, Uddin M, Shay DK, Waterman SH, Monto AS. Mask use, hand hygiene, and seasonal influenza-like illness among young adults: a randomized intervention trial. J Infect Dis. 2010;201(4):491-8. | **Population:** Studierende |
| Aiello et al., 2012 | Aiello AE, Perez V, Coulborn RM, Davis BM, Uddin M, Monto AS. Facemasks, hand hygiene, and influenza among young adults: a randomized intervention trial. PLoS One. 2012;7(1):e29744. | **Population:** Studierende |
| Al Naam et al., 2021 | Al Naam YA, Elsafi SH, Alkharraz ZS, Alfahad OA, Al-Jubran KM, Al Zahrani EM. Community practice of using face masks for the prevention of COVID-19 in Saudi Arabia. PLoS One. 2021 Feb 19;16(2):e0247313. | **Population:** Erwachsene |
| Amirav, 2013 | Amirav I. Evidence based design of face masks for infants. Int J Pharm. 2013;457(1):342-6. | **Exposition:** Inhalationsmasken |
| Anbarasu & Bhuvaneswari, 2020 | Anbarasu A, Bhuvaneswari M. Challenges Of The COVID-19 Pandemic For Child And Adolescent Mental Health: Promoting Psychosocial And Positive Well-Being. European Journal of Molecular & Clinical Medicine, 2020,7(7):326-333 | **Studiendesign:** Expertenmeinung |
| Anonym, 2009 | Anonym. The new influenza a virus: A/Mexico/2009 (H1N1) practice point for caregivers of children and youth. Can J Infect Dis Med Microbiol 2009,20(3):88. | **Studiendesign:** Abstract |
| Anonym, 2020 | Anonym. COVID-19 Stats: Percentage of Middle and High School Students Aged 13-21 Years Attending In-Person Classes Who Reported Observing Fellow Students Wearing a Mask All the Time,* by School Setting and Activity - United States, October 2020. MMWR Morb Mortal Wkly Rep. 2021 Feb 12;70(6):223. | **Studiendesign:** Statistik |
| Apanga et al., 2020 | Apanga PA, Kamal Lettor IB, Akunvane R. Practice of COVID-19 Preventive Measures and Its Associated Factors among Students in Ghana. Am J Trop Med Hyg. 2020 Dec 7;104(2):526–31. | **Population:** Studierende |
| Apidechkul, 2010 | Apidechkul, T. An outbreak of Influenza A (H1N1) 2009 at Mae Fah Luang University, Chiang Rai Province, northern Thailand. Retrovirology 2010,7:P187. | **Studiendesign:** Abstract |
| Barceló & Sheen, 2020 | Barceló J, Sheen GC. Voluntary adoption of social welfare-enhancing behavior: Mask-wearing in Spain during the COVID-19 outbreak. PLoS One. 2020 Dec 1;15(12):e0242764. | **Population:** Erwachsene |
| Bartek et al., 2021 | Bartek N, Peck JL, Garzon D, VanCleve S. Addressing the Clinical Impact of COVID-19 on Pediatric Mental Health. J Pediatr Health Care. 2021 Jul-Aug;35(4):377-386. | **Exposition:** COVID-19 Erkrankung allgemein |
| Beck, 2004 | Beck M, Antle BJ, Berlin D, Granger M, Meighan K, Neilson BJ, Shama W, Westland J, Kaufman M. Wearing masks in a pediatric hospital: developing practical guidelines. Can J Public Health. 2004;95(4):256-7. | **Studiendesign:** Kommentar |
| Betsch et al., 2021 | Betsch C, Korn L, Felgendreff L, Eitze S, Thaiss H. School opening during the SARS-CoV-2 pandemic: Public acceptance of wearing fabric masks in class. Public Health Pract (Oxf). 2021 Nov;2:100115. | **Outcome:** Öffentliche Akzeptanz von Masken |
| Beuvelet et al., 2017 | Beuvelet M, Masson C, Gantz D, Levet S, Allenet B, Mallaret MR, Landelle C. Healthcare workers’ perception towards the systematic use of mask during a seasonal influenza outbreak in a French University Hospital: a descriptive study. Antimicrobial Resistance and Infection Control 2017, 6(Suppl 3):52. | **Setting:** Krankenhaus |
| Biermann et al., 2021 | Biermann M, Schulze A, Unterseher F, Atanasova K, Watermann P, Krause-Utz A, Stahlberg D, Bohus M, Lis S. Trustworthiness appraisals of faces wearing a surgical mask during the Covid-19 pandemic in Germany: An experimental study. PLoS One. 2021 May 18;16(5):e0251393. | **Population:** Erwachsene |
| Bradley et al., 2020 | Bradley EH, An MW, Fox E. Reopening Colleges During the Coronavirus Disease 2019 (COVID-19) Pandemic-One Size Does Not Fit All. JAMA Netw Open. 2020;3(7):e2017838. | **Studiendesign:** Kommentar |
| Brambilla et al., 2020 | Brambilla I, Tosca MA, De Filippo M, Licari A, Piccotti E, Marseglia GL, Ciprandi G. Special Issues for Coronavirus Disease 2019 in Children and Adolescents. Obesity (Silver Spring). 2020;28(8):1369. | **Studiendesign:** Leserbrief |
| Burgess & Horii, 2012 | Burgess A, Horii M. Risk, ritual and health responsibilisation: Japan's 'safety blanket' of surgical face mask-wearing. Sociol Health Illn. 2012;34(8):1184-98. | **Studiendesign:** historische Analyse |
| Cenk et al., 2020 | Cenk M, Yegit C, Ergenekon A, Aksoy AT, Bilicen G, Gokdemir Y, Erdem E, Arman A, Karakoc F, Karadag B. (2020). Effect of COVID-19 Pandemic on Anxiety Levels of Children with Cystic Fibrosis and Healthy Children. 10.22541/au.160218230.00714939/v1. | **Exposition:** COVID-19 Erkrankung allgemein |
| Charney et al., 2021 | Charney SA, Camarata SM, Chern A. Potential Impact of the COVID-19 Pandemic on Communication and Language Skills in Children. Otolaryngol Head Neck Surg. 2021 Jul;165(1):1-2. | **Studiendesign:** Kommentar |
| Chaudhary et al., 2010 | Chaudhary V, Singh RK, Agrawal VK, Agarwal A, Kumar R, Sharma M. Awareness, perception and myths towards swine flu in school children of Bareilly, Uttar Pradesh. Indian J Public Health. 2010;54(3):161-4. | **Outcome:** Prävalenz des Maskentragens |
| Chen et al., 2020 | Chen X, Ran L, Liu Q, Hu Q, Du X, Tan X. Hand Hygiene, Mask-Wearing Behaviors and Its Associated Factors during the COVID-19 Epidemic: A Cross-Sectional Study among Primary School Students in Wuhan, China. Int J Environ Res Public Health. 2020;17(8):2893. | **Outcome:** Verhaltensweisen des Maskentragens |
| Cheng et al., 2020 | Cheng SY, Wang CJ, Shen AC, Chang SC. How to Safely Reopen Colleges and Universities During COVID-19: Experiences From Taiwan. Ann Intern Med. 2020;173(8):638-641. | **Studiendesign:** Kommentar |
| Cheok et al., 2021 | Cheok GJW, Gatot C, Sim CHS, Ng YH, Tay KXK, Howe TS, Koh JSB. Appropriate attitude promotes mask wearing in spite of a significant experience of varying discomfort. Infect Dis Health. 2021 May;26(2):145-151. | **Population:** Erwachsene |
| Crowe, 2020 | Crowe AL. Communication skills with children in paediatric anaesthesia: challenges while wearing a face mask. BMJ Paediatr Open. 2020;4(1):e000846. | **Studiendesign:** Kommentar |
| Cui et al., 2020 | Cui Y, Li Y, Zheng Y; Chinese Society of Child & Adolescent Psychiatry. Mental health services for children in China during the COVID-19 pandemic: results of an expert-based national survey among child and adolescent psychiatric hospitals. Eur Child Adolesc Psychiatry. 2020;29(6):743-748. | **Exposition:** COVID-19 Erkrankung allgemein |
| Davis et al., 2020 | Davies SH, Della Porta A, Renjilian CB, Sit L, Ginsburg KR. Lessons Learned: Achieving Critical Mass in Masking Among Youth in Congregate Living. J Adolesc Health. 2020;67(2):298-299. | **Studiendesign:** Leserbrief |
| Dawson et al., 2021 | Dawson P, Worrell MC, Malone S, Tinker SC, Fritz S, Maricque B, Junaidi S, Purnell G, Lai AM, Neidich JA, Lee JS, Orscheln RC, Charney R, Rebmann T, Mooney J, Yoon N, Petit M, Schmidt S, Grabeel J, Neill LA, Barrios LC, Vallabhaneni S, Williams RW, Goddard C, Newland JG, Neatherlin JC, Salzer JS; CDC COVID-19 Surge Laboratory Group. Pilot Investigation of SARS-CoV-2 Secondary Transmission in Kindergarten Through Grade 12 Schools Implementing Mitigation Strategies - St. Louis County and City of Springfield, Missouri, December 2020. MMWR Morb Mortal Wkly Rep. 2021 Mar 26;70(12):449-455. | **Outcome:** Krankheitsübertragung von SARS-CoV-2 |
| DeJonckheere et al., 2021 | DeJonckheere M, Waselewski M, Amaro X, Frank A, Chua KP. Views on COVID-19 and Use of Face Coverings Among U.S. Youth. J Adolesc Health. 2021 May;68(5):873-881. | **Population:** Erwachsene |
| Del Valle et al., 2010 | Del Valle SY, Tellier R, Settles GS, Tang JW. Can we reduce the spread of influenza in schools with face masks? Am J Infect Control. 2010;38(9):676-7. | **Studiendesign:** Kommentar |
| Dhakshinya et al., 2020 | M. Dhakshinya, S. Balaji Ganesh, D. Ezhilarasan. Awareness about usage of face mask and hand gloves-a questionnaire survey. International Journal of Pharmaceutical Research 2020; 2 (Suppl 2): 2592–2601. | **Population:** Erwachsene |
| Dias et al., 2020 | Dias JV, Contreiras M, Oom P. SARS-CoV-2 Pandemic: Should Children Wear Masks? Acta Med Port 2020;33(10):711. | **Studiendesign:** Kommentar |
| Ehrhardt et al., 2020 | Ehrhardt J, Ekinci A, Krehl H, Meincke M, Finci I, Klein J, Geisel B, Wagner-Wiening C, Eichner M, Brockmann SO. Transmission of SARS-CoV-2 in children aged 0 to 19 years in childcare facilities and schools after their reopening in May 2020, Baden-Württemberg, Germany. Euro Surveill. 2020 Sep;25(36):2001587. | **Outcome:** Krankheitsübertragung von SARS-CoV-2 |
| Ehrhardt et al., 2021 | Eberhart M, Orthaber S, Kerbl R. The impact of face masks on children-A mini review. Acta Paediatr. 2021 Jun;110(6):1778-1783. | **Outcome:** Maskentragen im Allgemeinen |
| Ersin & Kartal, 2020 | Ersin F, Kartal M. The determination of the perceived stress levels and health-protective behaviors of nursing students during the COVID-19 pandemic. Perspect Psychiatr Care. 2020:10.1111/ppc.12636. | **Population:** Studierende |
| Esposito & Principi, 2020 | Esposito S, Principi N. To mask or not to mask children to overcome COVID-19. Eur J Pediatr. 2020;179(8):1267-1270. | **Studiendesign:** Kommentar |
| Esposito & Principi, 2020 | Esposito S, Principi N. Mask-wearing in pediatric age. Eur J Pediatr. 2020;179(8):1341-1342. | **Studiendesign:** Antwortschreiben |
| Esposito et al., 2021 | Esposito, Susanna & Cotugno, Nicola & Principi, Nicola. (2021). Comprehensive and safe school strategy during COVID-19 pandemic. Italian Journal of Pediatrics. 47. 10.1186/s13052-021-00960-6. | **Studiendesign:** Debatte |
| Ferdous et al., 2020 | Ferdous MZ, Islam MS, Sikder MT, Mosaddek ASM, Zegarra-Valdivia JA, Gozal D. Knowledge, attitude, and practice regarding COVID-19 outbreak in Bangladesh: An online-based cross-sectional study. PLoS One. 2020;15(10):e0239254. | **Outcome:** Prävalenz des Maskentragens |
| Ferng et al., 2011 | Ferng YH, Wong-McLoughlin J, Barrett A, Currie L, Larson E. Barriers to mask wearing for influenza-like illnesses among urban Hispanic households. Public Health Nurs. 2011;28(1):13-23. | **Setting:** familiäres Umfeld |
| Fore, 2020 | Fore HH. A wake-up call: COVID-19 and its impact on children's health and wellbeing. Lancet Glob Health. 2020;8(7):e861-e862. | **Studiendesign:** Kommentar |
| Gallagher & Schleyer, 2020 | Gallagher TH, Schleyer AM. "We Signed Up for This!" - Student and Trainee Responses to the Covid-19 Pandemic. N Engl J Med. 2020;382(25):e96. | **Population:** Studierende |
| Ganesh et al., 2020 | Ganesh.S, Balaji & Dhakshinya, M. (2021). Awareness about Usage of Face Mask and Hand Gloves - A Questionnaire Survey. International Journal of Pharmaceutical Research. 12. 10.31838/ijpr/2020.SP2.283. | **Population:** Erwachsene |
| Gettings et al., 2021 | Gettings J, Czarnik M, Morris E, et al. Mask Use and Ventilation Improvements to Reduce COVID-19 Incidence in Elementary Schools — Georgia, November 16–December 11, 2020. MMWR Morb Mortal Wkly Rep 2021;70:779–784. | **Outcome:** Maskentragen im Allgemeinen |
| Gillespie et al., 2021 | Gillespie DL, Meyers LA, Lachmann M, Redd SC, Zenilman JM. The Experience of 2 Independent Schools With In-Person Learning During the COVID-19 Pandemic. J Sch Health. 2021 May;91(5):347-355. | **Outcome:** Maskentragen im Allgemeinen |
| Green, 2021 | Green J, Staff L, Bromley P, Jones L, Petty J. The implications of face masks for babies and families during the COVID-19 pandemic: A discussion paper. J Neonatal Nurs. 2021;27(1):21-25. | **Studiendesign:** Diskussionspapier |
| Grundmann et al., 2021 | Grundmann F, Epstude K, Scheibe S. Face masks reduce emotion-recognition accuracy and perceived closeness. PLoS One. 2021 Apr 23;16(4):e0249792. | **Population:** Erwachsene |
| Guimarães et al., 2020 | Guimarães AC, Mau LB, Maunsell RCK. COVID-19 in children: considerations for returning to school. Braz J Otorhinolaryngol. 2020 Nov-Dec;86(6):667-668. | **Studiendesign:** Editorial |
| Guzek et al., 2020 | Guzek D, Skolmowska D, Głąbska D. Analysis of Gender-Dependent Personal Protective Behaviors in a National Sample: Polish Adolescents' COVID-19 Experience (PLACE-19) Study. Int J Environ Res Public Health. 2020;17(16):5770. | **Outcome:** Prävalenz des Maskentragens |
| Hacımustafaoğlu, 2020 | Hacımustafaoğlu M. COVID-19 and re-opening of schools: Opinions with scientific evidence. Turk Pediatri Ars. 2020 Dec 16;55(4):337-344. | **Exposition:** COVID-19 Erkrankung allgemein |
| Hageman, 2020 | Hageman JR. Can Students Safely Return to School in the Age of COVID-19? Pediatr Ann. 2020;49(9):e363-e364. | **Studiendesign:** Editorial |
| Hajdúk et al., 2020 | Hajdúk M, Dančík D, Januška J, Svetský V, Straková A, Turček M, Vašečková B, Forgáčová Ľ, Heretik A, Pečeňák J. Psychotic experiences in student population during the COVID-19 pandemic. Schizophr Res. 2020;222:520-521. | **Studiendesign:** Leserbrief |
| Hashikawa et al., 2020 | Hashikawa AN, Sells JM, DeJonge PM, Alkon A, Martin ET, Shope TR. Child Care in the Time of Coronavirus Disease-19: A Period of Challenge and Opportunity. J Pediatr. 2020;225:239-245. | **Studiendesign:** Kommentar |
| Heyer, 2021 | Heyer C. The coronavirus through children's glasses. [German]. MMW Fortschr Med. 2021; 163 (3) | **Studiendesign:** Leserbrief |
| Hommes et al., 2021 | Hommes F, van Loon W, Thielecke M, Abramovich I, Lieber S, Hammerich R, Gehrke-Beck S, Linzbach E, Schuster A, von dem Busche K, Theuring S, Gertler M, Martinez GE, Richter J, Bergmann C, Bölke A, Böhringer F, Mall MA, Rosen A, Krannich A, Keller J, Bethke N, Kurzmann M, Kurth T, Kirchberger V, Seybold J, Mockenhaupt FP, Study Group B. SARS-CoV-2 Infection, Risk Perception, Behaviour and Preventive Measures at Schools in Berlin, Germany, during the Early Post-Lockdown Phase: A Cross-Sectional Study. Int J Environ Res Public Health. 2021 Mar 8;18(5):2739. | **Outcome:** Maskentragen im Allgemeinen |
| Hossain et al., 2020 | Hossain MA, Jahid MIK, Hossain KMA, Walton LM, Uddin Z, Haque MO, Kabir MF, Arafat SMY, Sakel M, Faruqui R, Hossain Z. Knowledge, attitudes, and fear of COVID-19 during the Rapid Rise Period in Bangladesh. PLoS One. 2020;15(9):e0239646. | **Outcome:** Prävalenz des Maskentragens |
| Howard, 2021 | Howard MC. The relations between age, face mask perceptions and face mask wearing. J Public Health (Oxf). 2021 Feb 10:fdab018. | **Population:** Erwachsene |
| Hyde, 2021 | Hyde Z. COVID-19, children and schools: overlooked and at risk. Med J Aust. 2021 Mar;214(4):190-191.e1. | **Studiendesign:** Meinung |
| Jin et al., 2020 | Jin K, Min J, Jin X. Re: Esposito et al.: To mask or not to mask children to overcome COVID-19. Eur J Pediatr. 2020;179(8):1339-1340. | **Studiendesign:** Antwortschreiben |
| Kalaskar et al., 2021 | Kalaskar, Ritesh & Balasubramanian, Shruti & Kalaskar, Ashita. (2021). Assessment of Awareness and Behavioural Patterns among Children Regarding COVID-19. JOURNAL OF CLINICAL AND DIAGNOSTIC RESEARCH. 15. 10.7860/JCDR/2021/47485.14925. | **Outcome:** Verhaltensweisen und Kenntnisse |
| Kecojevic et al., 2020 | Kecojevic A, Basch CH, Sullivan M, Davi NK. The impact of the COVID-19 epidemic on mental health of undergraduate students in New Jersey, cross-sectional study. PLoS One. 2020;15(9):e0239696. | **Population:** Studierende |
| Kerbl, 2021 | Kerbl R. COVID-19: Masken für Kinder? Eine Einschätzung von UNICEF und WHO [COVID-19: masks for children? An estimation by UNICEF and WHO]. Monatsschr Kinderheilkd. 2021 Jan 18:1-2. German. | **Studiendesign:** Zusammenfassung |
| Langbehn et al., 2020 | Langbehn, Andrew & Yermol, Dasha & Zhao, Fangyun Olivia & Thorstenson, Christopher & Niedenthal, Paula. (2020). Wearing N95, Surgical and Cloth Face Masks Compromises the Communication of Emotion. 10.21203/rs.3.rs-133686/v1. | **Population:** Erwachsene |
| Liu et al., 2020 | Liu X, Luo WT, Li Y, Li CN, Hong ZS, Chen HL, Xiao F, Xia JY. Psychological status and behavior changes of the public during the COVID-19 epidemic in China. Infect Dis Poverty. 2020;9(1):58. | **Exposition:** COVID-19 Pandemie im Allgemeinen |
| Ludvigsson, 2021 | Ludvigsson JF. Little evidence for facemask use in children against COVID-19. Acta Paediatr. 2021 Mar;110(3):742-743. | **Studiendesign:** Editorial |
| Maarefvand et al., 2020 | Maarefvand M, Hosseinzadeh S, Farmani O, Safarabadi Farahani A, Khubchandani J. Coronavirus Outbreak and Stress in Iranians. Int J Environ Res Public Health. 2020;17(12):4441. | **Population:** Erwachsene |
| Magee et al., 2020 | Magee M, Lewis C, Noffs G, Reece H, Chan JCS, Zaga CJ, Paynter C, Birchall O, Rojas Azocar S, Ediriweera A, Kenyon K, Caverlé MW, Schultz BG, Vogel AP. Effects of face masks on acoustic analysis and speech perception: Implications for peri-pandemic protocols. J Acoust Soc Am. 2020 Dec;148(6):3562. | **Population:** Erwachsene |
| Mallapaty, 2020 | Mallapaty S. How schools can reopen safely during the pandemic. Nature. 2020;584(7822):503-504. | **Studiendesign:** Zeitungsartikel |
| Matovu et al., 2021x | Matovu JKB, Kabwama SN, Ssekamatte T, Ssenkusu J, Wanyenze RK. COVID-19 Awareness, Adoption of COVID-19 Preventive Measures, and Effects of COVID-19 Lockdown Among Adolescent Boys and Young Men in Kampala, Uganda. J Community Health. 2021 Aug;46(4):842-853. | **Outcome:** Maskentragen im Allgemeinen |
| Matusiak et al., 2020 | Matusiak Ł, Szepietowska M, Krajewski P, Białynicki-Birula R, Szepietowski J. Face masks use during the COVID-19 pandemic: Differences in attitudes and practices between medical and non-medical students. A survey of 2256 students in Poland. Adv Clin Exp Med. 2020;29(10):1201-1203. | **Population:** Erwachsene |
| Matusiak et al., 2020 | Matusiak Ł, Szepietowska M, Krajewski P, Białynicki-Birula R, Szepietowski JC. Inconveniences due to the use of face masks during the COVID-19 pandemic: A survey study of 876 young people. Dermatol Ther. 2020;33(4):e13567. | **Population:** Erwachsene |
| Matusiak et al., 2020 | Matusiak Ł, Szepietowska M, Krajewski PK, Białynicki-Birula R, Szepietowski JC. The use of face masks during the COVID-19 pandemic in Poland: A survey study of 2315 young adults. Dermatol Ther. 2020;33(6):e13909. | **Population:** Erwachsene |
| Medvedev, 2020 | Medvedev MM. Management of mother-newborn dyads in the COVID-19 era. Lancet Child Adolesc Health. 2020;4(10):710-711. | **Studiendesign:** Kommentar |
| Mehta et al., 2020 | Mehta UM, Venkatasubramanian G, Chandra PS. The "mind" behind the "mask": Assessing mental states and creating therapeutic alliance amidst COVID-19. Schizophr Res. 2020;222:503-504. | **Studiendesign:** Leserbrief |
| Morishima & Kishida, 2018 | Morishima M, Kishida K. Understanding attitudes toward hygiene mask use in Japanese daily life by using a repeated cross-sectional survey. Work. 2018;61(2):303-311. | **Population:** Studierende |
| Muzzi et al., 2021 | Muzzi E, Chermaz C, Castro V, Zaninoni M, Saksida A, Orzan E. Short report on the effects of SARS-CoV-2 face protective equipment on verbal communication. Eur Arch Otorhinolaryngol. 2021 Jan 3:1–6. | **Population:** Erwachsene |
| Pearl, 2020 | Pearl PL. Child neurology, COVID-19, and crisis in society. Dev Med Child Neurol. 2020;62(10):1113. | **Studiendesign:** Editorial |
| Pereira-Ávila et al., 2021 | Pereira-Ávila FMV, Lam SC, Gir E, Góes FGB, Freire MEM, Silva ACOE. Factors associated to the practice of using masks by the population of Paraíba during the COVID-19 pandemic. Rev Esc Enferm USP. 2021 Jun 2;55:e03735. Portuguese, English. | **Population:** Erwachsene |
| Prousa, 2020 | Prousa. Studie zu psychologischen und psychovegetativen Beschwerden durch die aktuellen Mund-Nasenschutz-Verordnungen in Deutschland. 2020. | **Population:** Erwachsene |
| Proverbio & Cerri, 2021 | Proverbio, Alice & Cerri, Alice. (2021). Surgical Masks Impair People's Ability To Accurately Classify Emotional Expressions, Except For Anger. 10.21203/rs.3.rs-640471/v1. | **Population:** Studierende |
| Pourret & Saillet, 2020 | Pourret O, Saillet E. Wear your mask, but think about deaf students. Nature. 2020;586(7830):629-630. | **Studiendesign:** Zeitungsartikel |
| Rabinowicz et al., 2020 | Rabinowicz S, Leshem E, Pessach IM. COVID-19 in the Pediatric Population-Review and Current Evidence. Curr Infect Dis Rep. 2020;22(11):29. | **Setting:** klinisches Setting |
| Rader et al., 2021 | Rader B, White LF, Burns MR, Chen J, Brilliant J, Cohen J, Shaman J, Brilliant L, Kraemer MUG, Hawkins JB, Scarpino SV, Astley CM, Brownstein JS. Mask-wearing and control of SARS-CoV-2 transmission in the USA: a cross-sectional study. Lancet Digit Health. 2021 Mar;3(3):e148-e157. | **Population:** Erwachsene |
| Reszke et al., 2021 | Reszke R, Szepietowska M, Krajewski PK, Matusiak Ł, Białynicki-Birula R, Szepietowski JC. Face Mask Usage among Young Polish People during the COVID-19 Epidemic-An Evolving Scenario. Healthcare (Basel). 2021 May 27;9(6):638. | **Population:** Erwachsene |
| Roberge, 2011 | Roberge R. Facemask use by children during infectious disease outbreaks. Biosecur Bioterror. 2011;9(3):225-31. | **Outcome:** respiratorische Parameter |
| Scheid et al., 2020 | Scheid JL, Lupien SP, Ford GS, West SL. Commentary: Physiological and Psychological Impact of Face Mask Usage during the COVID-19 Pandemic. Int J Environ Res Public Health. 2020;17(18):6655. | **Studiendesign:** Kommentar |
| Sengul et al., 2020 | Sengul H, Bulut A, Coskun SN. Psychological effect of covid-19 pandemic on university students in turkey. Bayrakol 2020. | **Population:** Studierende |
| Shack et al., 2020 | Shack AR, Arkush L, Reingold S, Weiser G. Masked paediatricians during the COVID-19 pandemic and communication with children. J Paediatr Child Health. 2020;56(9):1475-1476. | **Setting:** Krankenhaus |
| Slimani et al., 2021 | Slimani M, Miarka B, Znazen H, Moalla W, Hammami A, Paravlic A, Bragazzi NL. Effect of a Warm-Up Protocol with and without Facemask-Use against COVID-19 on Cognitive Function: A Pilot, Randomized Counterbalanced, Cross-Sectional Study. Int J Environ Res Public Health. 2021 May 30;18(11):5885. | **Population:** Studierende |
| Smith et al., 2021 | Smith LE, Duffy B, Moxham-Hall V, Strang L, Wessely S, Rubin GJ. Anger and confrontation during the COVID-19 pandemic: a national cross-sectional survey in the UK. J R Soc Med. 2021;114(2):77-90. | **Population:** Erwachsene |
| Spitzer 2020 | Spitzer M. Masked education? The benefits and burdens of wearing face masks in schools during the current Corona pandemic. Trends Neurosci Educ. 2020;20:100138. | **Studiendesign:** Stellungnahme |
| Stebbins et al., 2009 | Stebbins S, Downs JS, Vukotich CJ Jr. Using nonpharmaceutical interventions to prevent influenza transmission in elementary school children: parent and teacher perspectives. J Public Health Manag Pract. 2009;15(2):112-7. | **Outcome:** Bereitschaft zum Maskentragen |
| Suess et al., 2011 | Suess T, Remschmidt C, Schink S, Luchtenberg M, Haas W, Krause G, Buchholz U. Facemasks and intensified hand hygiene in a German household trial during the 2009/2010 influenza A(H1N1) pandemic: adherence and tolerability in children and adults. Epidemiol Infect. 2011;139(12):1895-901. | **Setting:** familiäres Umfeld |
| Suess et al., 2012 | Suess T, Remschmidt C, Schink SB, Schweiger B, Nitsche A, Schroeder K, Doellinger J, Milde J, Haas W, Koehler I, Krause G, Buchholz U. The role of facemasks and hand hygiene in the prevention of influenza transmission in households: results from a cluster randomised trial; Berlin, Germany, 2009-2011. BMC Infect Dis. 2012;12:26. | **Setting:** familiäres Umfeld |
| Sujadi et al., 220 | Sujadi E, Fadhli M, Kamil D, Ridha Ds M, Sonafist Y, Meditamar MO, Ahmad B. An anxiety analysis of educators, students and parents facing the new normal era in education sector in indonesia. Asian J Psychiatr. 2020;53:102226. | **Exposition:** COVID-19 Pandemie im Allgemeinen |
| Tan et al., 2021 | Tan M, Wang Y, Luo L, Hu J. How the public used face masks in China during the coronavirus disease pandemic: A survey study. Int J Nurs Stud. 2021 Mar;115:103853. | **Population:** Erwachsene |
| Taylor et al., 2009 | Taylor M, Raphael B, Barr M, Agho K, Stevens G, Jorm L. Public health measures during an anticipated influenza pandemic: Factors influencing willingness to comply. Risk Manag Healthc Policy. 2009;2:9-20. | **Population:** Erwachsene |
| Teasdale et al., 2014 | Teasdale E, Santer M, Geraghty AW, Little P, Yardley L. Public perceptions of non-pharmaceutical interventions for reducing transmission of respiratory infection: systematic review and synthesis of qualitative studies. BMC Public Health. 2014;14:589. | **Population:** Erwachsene |
| Truong et al. 2006 | Truong J, Jain S, Tan J, Keegan D, Matsui D, Rieder MJ. Young children's perceptions of physicians wearing standard precautions versus customary attire. Pediatr Emerg Care. 2006;22(1):13-7. | **Setting:** Krankenhaus |
| Truong et al. 2021 | Truong TL, Beck SD, Weber A. The impact of face masks on the recall of spoken sentences. J Acoust Soc Am. 2021 Jan;149(1):142. | **Studiendesign:** Leserbrief |
| Uchida et al., 2016 | Uchida M, Kaneko M, Hidaka Y, Yamamoto H, Honda T, Takeuchi S, Saito M, Kawa S. Effectiveness of vaccination and wearing masks on seasonal influenza in Matsumoto City, Japan, in the 2014/2015 season: An observational study among all elementary schoolchildren. Prev Med Rep. 2016;5:86-91. | **Outcome:** Wirksamkeit von Masken |
| Verd et al., 2021 | Verd, S., Moll, P., Serra, C. and Ginovart, G. (2021), Mothers’ masks should be improved or removed during pandemic as they inhibit bonding with neonates. Acta Paediatr, 110: 1689-1689. | **Studiendesign:** Korrespondenz |
| Vereen et al., 2021 | Vereen RN, Lazard AJ, Frank SC, Pulido M, Richter APC, Higgins ICA, Shelus VS, Vandegrift SM, Hall MG, Ribisl KM. Motivations, barriers, and communication recommendations for promoting face coverings during the COVID-19 pandemic: Survey findings from a diverse sample. PLoS One. 2021 May 7;16(5):e0251169. | **Population:** Erwachsene |
| Villani et al., 2020 | Villani A, Bozzola E, Staiano A, Agostiniani R, Del Vecchio A, Zamperini N, Marino F, Vecchio D, Corsello G. Facial masks in children: the position statement of the Italian pediatric society. Ital J Pediatr. 2020;46(1):132. | **Studiendesign:** Kommentar |
| Walger et al., 2020 | Walger P, Heininger U, Knuf M, Exner M, Popp W, Fischbach T, Trapp S, Hübner J, Herr C, Simon A; German Society for Hospital Hygiene (DGKH); German Society for Pediatric Infectious Diseases (DGPI); German Academy for Pediatric and Adolescent Medicine (DAKJ); Society of Hygiene, Environmental and Public Health Sciences (GHUP); Professional Association of Pediatricians in Germany (bvkj e.V.). Children and adolescents in the CoVid-19 pandemic: Schools and daycare centers are to be opened again without restrictions. The protection of teachers, educators, carers and parents and the general hygiene rules do not conflict with this. GMS Hyg Infect Control. 2020;15:Doc11. | **Studiendesign:** Empfehlung der Fachgesellschaften |
| Wang et al., 2020 | Wang C, Chudzicka-Czupała A, Grabowski D, Pan R, Adamus K, Wan X, Hetnał M, Tan Y, Olszewska-Guizzo A, Xu L, McIntyre RS, Quek J, Ho R, Ho C. The Association Between Physical and Mental Health and Face Mask Use During the COVID-19 Pandemic: A Comparison of Two Countries With Different Views and Practices. Front Psychiatry. 2020;11:569981. | **Population:** Erwachsene |
| Wang et al., 2020 | Wang C, Pan R, Wan X, Tan Y, Xu L, Ho CS, Ho RC. Immediate Psychological Responses and Associated Factors during the Initial Stage of the 2019 Coronavirus Disease (COVID-19) Epidemic among the General Population in China. Int J Environ Res Public Health. 2020;17(5):1729. | **Population:** Erwachsene |
| Wang et al., 2020 | Wang C, Pan R, Wan X, Tan Y, Xu L, McIntyre RS, Choo FN, Tran B, Ho R, Sharma VK, Ho C. A longitudinal study on the mental health of general population during the COVID-19 epidemic in China. Brain Behav Immun. 2020;87:40-48. | **Population:** Erwachsene |
| Wong & Tang, 2005 | Wong CY, Tang CS. Practice of habitual and volitional health behaviors to prevent severe acute respiratory syndrome among Chinese adolescents in Hong Kong. J Adolesc Health. 2005;36(3):193-200. | **Outcome:** Prävalenz des Maskentragens |
| Xiao et al., 2020 | Xiao H, Shu W, Li M, Li Z, Tao F, Wu X, Yu Y, Meng H, Vermund SH, Hu Y. Social Distancing among Medical Students during the 2019 Coronavirus Disease Pandemic in China: Disease Awareness, Anxiety Disorder, Depression, and Behavioral Activities. Int J Environ Res Public Health. 2020;17(14):5047. | **Population:** Studierende |

## 5. Datenextraktion der Primärstudien

| **Studie** | **Allgemein** | **Setting** | **Population** | **Exposition/ Intervention** | **Kontrolle/ Vergleich** | **Outcome** | **Ergebnisse** | **Kommentare** |
| --- | --- | --- | --- | --- | --- | --- | --- | --- |
| Erstautor:in, Jahr | Design:  Land:  Zeitraum der Studie:  Anzahl der Untersuchungswellen:  Dauer des Follow-ups (z. B. Mittelwert, Ausmaß):  Name der Kohorte: | Beschreibung:  Einschlusskriterien:  # eingeladen:  # Baseline:  # Follow-up:  Response:  Loss to follow-up: | Beschreibung der Zielpopulation:  Einschlusskriterien:  # eingeladen:  # Baseline:  # Follow-up:  Alter:  % weiblich:  Response:  Loss to follow-up: | Beschreibung und Bewertung | Beschreibung und Bewertung | Beschreibung und Bewertung | Beschreibung themenrelevanter Ergebnisse | Finanzierung *(keine, öffentliche, Non-Profit-, Industrie-Finanzierung, nicht berichtet)*  Interessenkonflikt *(keiner, vorhanden, nicht berichtet)*  Einverständnis einer Ethikkommission *(ja, nein, nicht berichtet)*  Stärken (+) und Schwächen (-) |
| Allison, 2010 | **Design:** unkontrollierte Interventionsstudie (Pilotstudie)  **Land:** USA, Salt Lake City  **Zeitraum der Studie:** Beginn: 22. Januar 2007, Dauer: 4 Wochen  **Anzahl der Untersuchungswellen:** *n* = 1 | **Beschreibung:** Grundschule (1.–6. Klasse)  **Einschlusskriterien:** k.A.  **# eingeladen:** k.A.  **# Baseline:** Schulen: *n* = 2  Klassen: *n* = 19  **Response:** k.A. | **Beschreibung:** Grundschulkinder (und Lehrer:innen)  **Einschlusskriterien:** k.A.  **# eingeladen:** k.A.  **# Baseline:** *n* = 503  **Alter:** k.A.  **% weiblich:** k.A.  **Response:** k.A. | **Verwendung von Händedesinfektion und Gesichtsmasken zur Prävention von Influenza** durch Lehrer:innen und Schüler:innen (2 Phasen):  - Phase 1: Nutzung der Händedesinfektion in den ersten 2 Wochen  - Phase 2: Tragen der Masken in den zweiten 2 Wochen (OP-Masken), unabhängig vom Erkrankungs-stadium, mit täglich neuen Masken, in Kindergröße mit Disney-Motiven für Kindergarten und 4. Klasse und Erwachsenengröße für 5./6. Klasse und Lehrerschaft | nicht zutreffend | **Barrieren des Maskentragens:**  - Ablenkung  - körperliches Unwohlsein  - Schwierigkeit beim Lesen von Gesichts-ausdrücken (offene Fragen im Fragebogen für die Lehrer:innen, einschließlich schriftlicher Kommentare der Schüler:innen, Rück-meldungen in Follow-up-Meetings) | **Deskriptive Ergebnisse:**   - Masken führten zu Ablenkung und körperlichem Unwohlsein - Tragen von Masken erschwerten für Schüler:innen und Lehrer:innen das Erkennen der Gesichtsausdrücke der Anderen (Zitat: *„Gesichtsausdrücke sind ein wichtiger Bestandteil der Kommunikation und ich fühlte mich beim Tragen der Masken gehemmt.“*) - einige Lehrer:innen erwähnten, dass sie ihren Unterrichtsstil ändern müssten, wenn sie im Falle einer Influenza-Pandemie Masken tragen müssten - Adhärenz gegenüber den Gesichtsmasken sank über den Studienzeitraum | **Finanzierung:** öffentliche und industrielle Förderung (Autor:innen geben an, dass sie keine finanzielle Beziehung mit dem Förderunternehmen hatten)  **Interessenkonflikt:** k.A.  **Einverständnis einer Ethikkommission:** ja  + freiwillige Intervention  + Intervention fand unter Realbedingungen statt  + der Fragebogen der Lehrer:innen basiert auf dem Health-Belief-Modell, wurde selbst entwickelt und wurde mit Lehrer:innen einer anderen Schule in einem Vortest getestet  - keine Vergleichsgruppe vorhanden  - keine Informationen zum Rekrutierungsprozess und zur Response  - deskriptive Ergebnisauswertung basierend auf subjektiven Aussagen basierend auf Aussagen der Lehrerschaft, nicht der Kinder selbst  - geringe Akzeptanz und Adhärenz des Maskentragens  - keine Berücksichtigung der Confounder Alter und Geschlecht in den Ergebnisanalysen  - kurze Interventionsdauer |
| Coniam, 2005 | **Design:** Mixed-Methods-Studie:  1. Prüflinge (Schülerinnen):  - Umfrage  - 10 % der Kohorte: qualitative Interviews  2. Untersucher:innen:  - schriftlicher Bericht  - ausführliche Interviews mit Fokusgruppen von 4 Untersucher:innen  **Land:** Hongkong  **Zeitraum der Studie:** März 2004 (1 Jahr post-SARS)  **Anzahl der Untersuchungswellen:** *n* = 1 | **Beschreibung:** Schule mit Schülerinnen, Klassenstufe 11  **Einschlusskriterien:** k.A.  **# eingeladen:** k.A.  **# Baseline:**  Schule: *n* = 1  Klassen: *n* = 5  **Response:** k.A. | **Beschreibung:** Schülerinnen (Prüflinge) der 11. Klasse einer Mädchenschule  **Einschlusskriterien:** k.A.  **# eingeladen:** k.A.  **# Baseline:** *n* = 186  **Alter:** k.A.  **% weiblich:** 100%  **Response:** k.A. | **Tragen chirurgischer Masken während der SARS-Epidemie,** die 2003 in Hong Kong auftrat, durch die Prüflinge und Untersu-cher:innen während einer mündlichen Englischprüfung für die 11. Klasse  - Prüfung dauert 10 Minuten pro Prüfling und besteht aus 2 Teilen:  1. Rollenspiel zwischen Prüfling und Untersucher:innen  2. Gruppen-diskussion zwischen 4 Prüflingen | **Kontrollbedingung:** Prüfsituation ohne Maskentragen | **- mündliche Fähigkeiten** (einschließlich Aussprache, Vokabular, Grammatik, Interaktion, Verständlichkeit, Hörbarkeit): mündlicher Test (6 Level für jede Dimension, 1: Vorschullevel, 6: Expertenlevel)  - **Prüfleistung:** Einzelfrage (für Prüflinge)  - **Verständnis des Gesagten:** Einzelfrage (für Prüflinge)  - **Sprachlaut-stärke und Sprechtempo** beim Masken-tragen: Einzelfrage (für Prüflinge)  **- fehlende nonverbale Kommunikation**, einschließlich Gesichtsausdrücke: Interviews mit 10% der Prüflinge, schriftliche Berichte der Untersucher:innen, Fokus-gruppendiskussion mit Untersu-cher:innen | **Mündliche Fähigkeiten** (t Test)   \|  \| Ohne Maske  M (SD) \| Mit Maske  M (SD) \| p \| \| --- \| --- \| --- \| --- \| \| **Rollenspiel** \|  \|  \|  \| \| Aussprache \| 3,56 (1,33) \| 3,35 (0,77) \| 0,68 \| \| Vokabular \| 3,40 (0,69) \| 3,33 (0,66) \| 0,301 \| \| Grammatik \| 3,54 (0,83) \| 3,49 (0,77) \| 0,507 \| \| Verständlichkeit \| 3,72 (0,77) \| 3,70 (0,77) \| 0,794 \| \| Hörbarkeit \| 3,62 (0,70) \| 3,57 (0,78) \| 0,472 \| \| **Gruppen-diskussion** \|  \|  \|  \| \| Aussprache \| 3,37 (0,74) \| 3,33 (0,77) \| 0,581 \| \| Vokabular \| 3,27 (0,85) \| 3,35 (0,81) \| 0,332 \| \| Grammatik \| 3,34 (0,85) \| 3,34 (0,86) \| 0,931 \| \| Verständlichkeit \| 3,46 (0,87) \| 3,47 (0,85) \| 0,840 \| \| Hörbarkeit \| 3,37 (0,87) \| 3,39 (0,86) \| 0,774 \| \| Interaktion \| 3,54 (0,94) \| 3,62 (0,96) \| 0,402 \|   **Prüfleistung und Verständnis des Gesagten** (t Test)   \|  \| Ohne Maske  M (SD) \| Mit Maske  M (SD) \| p \| \| --- \| --- \| --- \| --- \| \| **Prüfleistung …** (Skala 1–7, 1: geringstes Level) \|  \|  \|  \| \| … im Rollenspiel \| 4,53 (0,96) \| 3,92 (0,98) \| < 0,001 \| \| … in Grppendiskussion \| 4,19 (1,05) \| 3,54 (1,09) \| < 0,00 \| \| **Verständnis des Gesagten in der Gruppen-diskussion** (Skala: 1–3, 3: höheres Verständnis) \| 2,28 (0,74) \| 1,48 (0,60) \| < 0,00 \|   **Sprechtempo und Sprachlautstärke beim Maskentragen** (Prävalenz)   \|  \| **%** \| \| --- \| --- \| \| **Sprechtempo** \|  \| \| Viel langsamer als ohne Maske \| 9,7 \| \| Etwas langsamer als ohne Maske \| 62,1 \| \| **Sprachlautstärke** \|  \| \| Viel lauter als ohne Maske \| 2,6 \| \| Etwas lauter als ohne Maske \| 56,3 \| \| Gleiche Sprachlautstärke \| 11,1 \|   **Zitate der Prüflinge während der Interviews:**   - *“Ich konnte die Gesichter der Anderen beim Maskentragen nicht sehen.“* - *„Beim Maskentragen verschwanden alle Gesichtsausdrücke.“*   **Deskriptive Ergebnisse der Untersucher:innen aus schriftlichen Berichten:**   - 7 von 15 Untersucher:innen empfanden, dass sie in der Prüfsituation, in der Masken getragen wurden, etwas nachsichtiger bewerteten   🡪 Post-hoc-Vergleich der Testergebnisse mittels t Test zwischen der „nachsichtigeren“ Gruppe und der „Standardgruppe“ zeigte jedoch keinen statistisch signifikanten Unterschied   - 7 Untersucher:innen empfanden die Sprachsignale der Prüflinge in der Maskensituation verminderter; 8 Untersucher:innen sahen dabei keine Probleme; 1 Untersucher:in kommentierte jedoch: *„Ich denke, sie waren mit Masken viel besser, ich hatte den Eindruck, dass sie sich freier zu Sprechen fühlten.“* - 8 Untersucher:innen gaben an, dass [die Prüflinge] proaktiv versuchten, das eigene Verhalten beim Maskentragen zu verändern; wobei einige *„mehr Körpersprache nutzten“*, *„mehr Augenkontakt herstellten“* und *„lauter sprachen“*   🡪 einige dieser Ergebnisse wurden in den Interviews mit den Untersucher:innen bestätigt | **Finanzierung:** k.A.  **Interessenkonflikt:** k.A.  **Einverständnis einer Ethikkommission:** k.A.  + Messung der mündlichen Fähigkeit mit einem validierten Instrument  + Messung der Reaktionen und Wahrnehmungen des Maskentragens während des Englischtests sowohl durch Schülerinnen selbst als auch durch Untersucher:innen  + Analysemethoden (Multifacetten-Rasch-Analyse, Chi-Quadrat-Statistik, t Test, qualitative Interviewauswertung)  + 8 verschiedene Testversionen wurden verwendet, um sicherzugehen, dass die Schülerinnen mit unterschiedlichen Tests getestet wurden und sie ihren Mitschülerinnen keine Hinweise zum Test zwischen den Testsessions geben konnten  + die Confounder Alter und Geschlecht sollten auf die Ergebnisse keinen Einfluss haben, da nur Schülerinnen einer bestimmten Jahrgangsstufe untersucht wurden  - keine Informationen zum Rekrutierungsprozess und zur Response  - kein echter Englischtest, sondern ein Probetest (auch wenn in der Studie versucht wurde, die echte Testsituation so real wie möglich darzustellen)  - alle zu prüfenden Schülerinnen legten 2 Versionen der mündlichen Prüfung ab (eine mit und eine ohne Maske), aber es ist unwahrscheinlich, dass durch einen „Übungseffekt“ der 2. Test zu einem besseren Testergebnis führte, da Schülerinnen der 11. Klasse in Hongkong mit dieser Art der mündlichen Testsituation vertraut sind  - Untersucher:innen der mündlichen Tests waren nicht gegenüber dem Expositionsstatus verblindet  - Zeitfaktor: zu Beginn der SARS-Epidemie hätten sich Gesichtsmasken zweifelsohne unangenehm angefühlt; nach einer gewissen Zeit des Tragens dürften sich die Menschen daran angepasst haben |
| Gori, 2021 | **Design:** Experimentalstudie  **Land:** Italien  **Zeitraum der Studie:** Mai 2020 (erste 2 Wochen des ersten Lockdowns in Italien)  **Anzahl der Untersuchungswellen:** *n* = 1 | **Beschreibung:** experimentelles Setting (Fotos wurden über ein Smartphone zu Hause gezeigt, Unterstützung der Kleinkinder durch Bezugsperson)  **Einschlusskriterien:** k.A.  **# eingeladen:** nicht zutreffend  **# Baseline:** k.A.  **Response:** k.A. | **Beschreibung:** Kinder im Alter von 3–5 Jahren und 6–8 Jahren (und Erwachsene)  **Einschlusskriterien:** Italienisch als Muttersprache  **# eingeladen:** k.A  **# Baseline:** *N* = 81  3–5-Jährige: *n* = 31  6–8-Jährige: *n* = 49  (Erwachsene: *n* = 39)  **Alter:**  3–5-Jährige: *M* = 4,3 Jahre (SD: 0,7)  6–8-Jährige: *M* = 6,8 Jahre (SD: 0,8)  (Erwachsene: *M* = 27,4 Jahre (SD: 2,1))  **% weiblich:**  k.A.  **Response:** k.A. | **Tragen einer Maske:**  - Experiment, in dem Kinder aufgefordert wurden Gesichter bestimmten Emotionen zuzuordnen  - Fotos von 4 Emotionen (Freude, Wut, Angst, Traurigkeit) in 2 Intensitätsgraden (mild, extrem) und einem neutralen Gesichtsaustruck 🡪 insgesamt 40 Fotos (4x4 Emotionen je mit und ohne Maske, 8x neutraler Gesichtsausdruck) in zufälliger Reihenfolge gezeigt  - Fotos einer entsprechenden Datenbank entnommen (ER-40 color emotional stimuli database) | **Kein Maskentragen:**  Experiment in der gleichen Art und Weise, aber es wurde das gesamte Gesicht ohne Maskierung der Mundpartie gezeigt | **Erkennen emotionaler Gesichtsausdrücke:**  richtige Zuordnung zu folgenden 4 Emotionen:  1. Traurigkeit  2. Wut  3. Angst  4. Freude  - Operationalisierung: a) Anteil richtiger Zuordnung mit und ohne Maske, b) Beeinträchtigung durch das Maskentragen (Differenz des Anteils richtiger Zuordnung mit und ohne Maske) | **Richtige Zuordnung der Emotionen:**  **1. zweifache ANOVA:**   - statistisch signifikanter Haupteffekt der Testbedingung: *F*(1,116) = 48,7, p < 0,001, Effektstärke = 0,4 - statistisch signifikanter Haupteffekt der Altersgruppe: *F*(1,116) = 190,2, p < 0,001, Effektstärke = 0,2 - statistisch signifikante Interaktion zwischen Testbedingung x Altersgruppe [*F*(2,116) = 29, *p*< 0,001, *η_p_^2^*= 0,1]   **2. t-Test innerhalb der Gruppe:**   - Kinder 3–5 Jahre: *t*(30) = 11,94, *p*< 0,001 - Kinder 6–8 Jahre: *t*(30) = 4,61, *p*< 0,001 - (Erwachsene: *t*(30) = 8,1, *p*< 0,001) - in allen Altersklassen war der Anteil richtiger Zuordnungen für die Bilder mit Maske geringer als für die Bilder ohne Maske   **3.** **Vergleich zwischen den Altersgruppen mittels t-Test:**  **3a): richtige Zuordnung von Emotionen bei Bildern ohne Maske:**   - Kinder 3–5 Jahre vs. Erwachsene: *t*(40,2) = 7,1, *p* < 0,001 - Kinder 6–8 Jahre vs. Erwachsene: *t*(78,9) = 8,8, *p* < 0,001 - Kinder 3–5 Jahre vs. Kinder 6–8 Jahre: *t*(54) = -0,88, *p* = 0,9 - Kindern im Alter von 3–5 und 6–8 Jahren fällt die richtige Zuordnung von Emotionen bei Bildern ohne Maske schwerer als Erwachsenen, wobei es keinen Unterschied zwischen den Kleinkindern und Grundschulkindern gibt   **3b): richtige Zuordnung von Emotionen bei Bildern mit Maske:**   - Kinder 3–5 Jahre vs. Erwachsene: *t*(42,2) = 9,02, *p* < 0,001 - Kinder 6–8 Jahre vs. Erwachsene: *t*(85,2) = 4,9, *p* < 0,001 - Kinder 3–5 Jahre vs. Kinder 6–8 Jahre: *t*(49) = -5,52, *p* < 0,001 - Kindern im Alter von 3–5 und 6–8 Jahren fällt die richtige Zuordnung von Emotionen bei Bildern mit Maske schwerer als Erwachsenen, wobei es Kleinkindern im Vergleich zu Grundschulkindern noch schwerer fällt   **Beeinträchtigung des Erkennens von Emotionen durch das Maskentragen:**  **1. einfache ANOVA:** statistisch signifikante Beeinträchtigung aufgrund des Maskentragens: *F*(1,116) = 28,96, p < 0,001, Effektstärke = 0,3  **2. Vergleich zwischen den Altersgruppen mittels t-Test:**   - Kinder 3–5 Jahre vs. Erwachsene: *t*(54,4) = -5,4, *p* < 0,001 - Kinder 6–8 Jahre vs. Erwachsene: *t*(85,9) = -2,1, *p* = 0,1 - Kinder 3–5 Jahre vs. Kinder 6–8 Jahre: *t*(60,1) = 6,89, *p* < 0,001 - der negative Effekt des Maskentragens war bei Kleinkindern statistisch signifikant stärker ausgeprägt im Vergleich zu Grundschulkindern oder Erwachsenen - allerdings gab es dahingehend keinen Unterschied zwischen Grundschulkindern und Erwachsenen | **Finanzierung:** k.A.  **Interessenkonflikt:** keiner  **Einverständnis einer Ethikkommission:** ja  + elterliche Zustimmung  + Auswahl der Fotographien aus validierter Datenbank  + zufällige Reihenfolge der Fotos  + Untersuchung des Einflusses der Confounders Alter auf die Ergebnisse  + statistische Analyse (ANOVA, t-Test)  - keine Informationen zum Rekrutierungsprozess und zur Response  - keine Untersuchung des Einflusses des Confounders Geschlecht auf die Ergebnisse  - experimentelles Setting (Exposition wurde nicht unter Realbedingungen untersucht)  - Darstellung der Bilder über kleinen Smartphon-Bildschirm  - eindimensionales Outcome (Ja-Nein-Entscheidung)  - keine Untersuchung eines zeitlichen Zusammen-hangs |
| Mickells, 2021 | **Design:** Längsschnittstudie  **Land:** USA, Atlanta  **Zeitraum der Studie:** 17. August–11. September 2020 (Beginn des Schuljahres 2020/21)  **Anzahl der Untersuchungswellen:** *n* = 19 (tägliche Abfrage an 19 Schultagen)  **Dauer des Follow-ups:** 4 Wochen | **Beschreibung:** Kinderkrippe, Kindergärten sowie 1. und 2. Klassen der Grundschulen eines Schulbezirks katholischer Schulen in Atlanta, USA  **Einschlusskriterien:** k.A.  **# eingeladen:** k.A.  **# Baseline:**  Schulen: *n* = 15 (davon 8 Schulen mit Kinderkrippe)  Klassen: Kinderkrippe: *n* = 10, Kindergarten: *n* = 28, 1. Klasse: *n* = 27, 2. Klasse: *n* = 27  **Response:** k.A. | **Beschreibung:** Kinder, die Kinderkrippe, Kindergärten sowie 1. und 2. Klassen der Grundschule besuchten (und Erzieher:innen und Lehrer:innen)  **Einschlusskriterien:** k.A.  **# eingeladen:** nicht zutreffend  **# Baseline:** *n* = 983 (Schätzung)  **Alter:** k.A.  **% weiblich:** k.A.  **Response:** k.A. | **Tragen von Gesichtsmasken im Kindergarten und in der Schule zur Prävention von COVID-19:** Maskenpflicht bestand für gesamten Schulbezirk | nicht zutreffend | **Unerwünschte Ereignisse durch das Maskentragen:**  - Stresserleben, Angst, Zusammenbruch  - Frustration | **Deskriptive Ergebnisse der Beobachtungen:**   - Insgesamt geringe Zahl berichteter unerwünschter Ereignisse (*n*= 59) - die am häufigsten berichteten unerwünschten Ereignisse im Zusammenhang mit dem Tragen der Masken waren Stresserleben, Angst und psychischer Zusammenbruch (*n*= 13) - Lehrerschaft berichtete zudem von Frustration der Kinder über Schwierigkeiten beim Hören und Kommunizieren mit den Erzieher:innen und Lehrer:innen sowie Mitschüler:innen (*n*= 8) - Zunahme von Einnässen („Bathroom accidents“) aufgrund der erschwerten Kommunikation (kein Erkennen der Mimik und erschwertes Hören) (*n* = 2) | **Finanzierung:** öffentliche Förderung  **Interessenkonflikt:** keiner  **Einverständnis einer Ethikkommission:** k.A.  + Untersuchung fand unter Realbedingungen statt  + Einladung aller Erzieher:innen und Lehrer:innen deren E-Mail-Adressen bekannt waren (Vollerhebung)  + Aufklärung im Vorfeld über Wirkung und mögliche Nebenwirkungen des Maskentragens  + Längsschnittuntersuchung  - keine Vergleichsgruppe vorhanden  - keine Angabe zur Response  - es wurde nicht berichtet, ob der Fragebogen validiert wurde oder nicht  - deskriptive Ergebnisauswertung basierend auf subjektiven Aussagen basierend auf Aussagen der Lehrerschaft, nicht der Kinder selbst  - keine Berücksichtigung der Confounder Alter und Geschlecht in den Ergebnisanalysen  - nach Aussage der Studienautor:innen geringe Repräsentativität, da nur katholische Schulen teilnahmen (weniger Persons of Color, Kinder mit besonderen Bedürfnissen und geringerem sozioökonomischen Status) |
| Qin, 2021 | **Design:** Querschnittsstudie  **Land:** China, Guangdong-Region  **Zeitraum der Studie:** 8.–30. März 2020  **Anzahl der Untersuchungswellen:** *n* = 1 | **Beschreibung:** Grundschulen und weiterführende Schulen (10 %aller Schulen in jeder Stadt der Region)  **Einschlusskriterien:** k.A.  **# eingeladen:** k.A.  **# Baseline:** k.A.  **Response:** k.A. | **Beschreibung:** schulpflichtige Kinder und Jugendliche  **Einschlusskriterien:** k.A.  **# eingeladen:** k.A  **# Baseline:** beantwortete Fragebögen: *n* = 1.310.600. valide Fragebögen (genutzt für Analysen): *n* = 1.199.320  **Alter:**  *M =*12,4 Jahre (SD: 3,01)  **% weiblich:**  48,4 %  **Response:** k.A. | **Tragen einer Maske während der COVID-19-Pandemie in Situationen**, in denen es empfohlen wurde:  - 4 Abstufungen: immer, meistens, manchmal, selten | **Kein Maskentragen:**  - 1 Abstufung nie | **Psychische Belastung:** General Health Questionnaire (GHQ-12) (Wert ≥ 3: psychologische Belastung) | **Zusammenhang zwischen der Häufigkeit des Maskentragens und der psychologischen Belastung** (multivariate logistische Regression)   \|  \| OR (95 % KI) \| \| --- \| --- \| \| **Unadjustiert** \| \| \| Immer \| 1,0 \| \| Meistens \| 1,44 (1,41–1,46) \| \| Manchmal \| 1,77 (1,70–1,83) \| \| Selten \| 2,19 (2,09–2,30) \| \| Nie \| 2,64 (2,45–2,83) \| \| **Adjustiert für die geographische Region** \| \| \| Häufigkeit des Maskentragens \| 1,39 (1,18–1,64) \| | **Finanzierung:** öffentliche Förderung  **Interessenkonflikt:** keiner  **Einverständnis einer Ethikkommission:** ja  + elterliche Zustimmung  + zweistufiges, randomisiertes Cluster-Sampling: 1.) Schulen, 2.) Schüler:innen  + Abstufung der Expositionsvariable  + valides und reliables Instrument zur Erhebung des Outcomes  + statistische Analyse (multivariate logistische Regression)  + hohe Repräsentativität aufgrund der Rekrutierungsmethode und der hohen Stichprobengröße  - keine Angabe zur Response  - keine Berücksichtigung der Confounder Alter und Geschlecht  - keine Untersuchung eines zeitlichen Zusammen-hangs |
| Rao, 2006 | **Design:** Mixed-Methods-Studie:  - Beobachtungen: durchgeführt durch 3 erfahrene Dozierende  - Umfrage mit 10 Rektor:innen der Vorschulen  **Land:** Hongkong  **Zeitraum der Studie:**  - Beobachtungen: innerhalb eines Monats nach Schulwiedereröffnungen (18./19. Mai 2003)  - Umfrage mit Rektor:innen: Juli–August 2003  **Anzahl der Untersuchungswellen:** *n* = 1 | **Beschreibung:** Vorschulen (Kindergärten {für Kinder von 2 Jahren 8 Monaten bis 6 Jahren] und Kindertagesstätten [für Kinder von 6 Wochen bis 6 Jahren])  **Einschlusskriterien:** k.A.  **# eingeladen:** k.A.  **# Baseline:**  Beobachtungen: *n* = 20 (Kindergärten: *n* = 18, Kindertagesstätten: *n* = 2)  Umfrage mit Rektor:innen: *n* = 10  **Response:** k.A. | **Beschreibung:** Kinder (und Erzieher:innen) von Vorschulen  **Einschlusskriterien:** k.A.  **# eingeladen:** nicht zutreffend  **# Baseline:** nicht zutreffend  **Alter:** k.A.  **% weiblich:** k.A.  **Response:** nicht zutreffend | **Tragen von Gesichts-masken in Vorschulen während der SARS-Epidemie** während der Wiederöffnung von Schulen nach einer 6-wöchigen Schulschließungen, welche zwischen dem 29 März und dem 18. Mai 2003 bestanden (durch Kinder und Erzieher:innen) | nicht zutreffend | **Soziale Interaktion:** Fragebogen der 3 Beobach-tenden maß soziale Interaktion mit der Kategorie „soziale Interaktion“ (6 Fragen) | **Deskriptive Ergebnisse der Beobachtungen:**  **Kategorie:** Veränderungen der sozialen Interaktion:   - Kinder und Erzieher:innen konnten die Gesichtsausdrücke der Anderen wegen der Gesichtsmaske nicht erkennen - Kinder empfanden Unbehagen beim Tragen der Masken | **Finanzierung:** k.A.  **Interessenkonflikt:** k.A.  **Einverständnis einer Ethikkommission:** k.A.  + Expositionsstatus war während der SARS-Epidemie im stark betroffenen Land Hong Kong  + untersuchte Vorschulen waren repräsentative für Hongkonger Vorschulen hinsichtlich des Standortes, Förderung, Status, Größe, und Studiengebühren (aber es wurde nicht berichtet, wie diese Informationen erhoben wurden)  - nicht-zufällige Auswahl der Stichprobe  - es wurde nicht berichtet, ob der Fragebogen validiert wurde oder nicht  - deskriptive Ergebnisauswertung basierend auf subjektiven Aussagen der Forschenden, nicht der Kinder selbst  - keine Berücksichtigung der Confounder Alter und Geschlecht in den Ergebnisanalysen |
| Roberson, 2012 | **Design:** Experimentalstudie  **Land:** Großbritannien, Essex  **Zeitraum der Studie:** k.A.  **Anzahl der Untersuchungswellen:** *n* = 1 | **Beschreibung:** experimentelles Setting in einer ruhigen Umgebung (Experiment wurde vor einen PC durchgeführt, Fotos wurden auf einem Laptop-Bildschirm gezeigt)  **Einschlusskriterien:** k.A.  **# eingeladen:** nicht zutreffend  **# Baseline:** k.A.  **Response:** k.A. | **Beschreibung:** Kinder (3–4 Jahre, 5–6 Jahre, 7–8 Jahre, 9–10 Jahre)  **Einschlusskriterien:** k.A.  **# eingeladen:** k.A.  **# Baseline:** *N* =80  3–4-Jährige: *n* = 20  5–6-Jährige: *n* = 20  7–8-Jährige: *n* = 20  9–10-Jährige: *n* = 20  **Alter:**  3–4-Jährige: *M* = 3 Jahre 7 Monate  5–6-Jährige: *M* = 5 Jahre 6 Monate  7–8-Jährige: *M* = 7 Jahre 8 Monate  9–10-Jährige: *M* = 9 Jahre 7 Monate  **% weiblich:**  3–4-Jährige: 45% (*n* = 9)  5–6-Jährige: 55% (*n* = 11)  7–8-Jährige: 55% (*n* = 11)  9–10-Jährige: 40% (*n* = 8)  **Response:** k.A. | **Maskierung der Mundpartie:**  - Experiment, in dem Kinder aufgefordert wurden Gesichter bestimmten Emotionen zuzuordnen (Fotos einer Frau und eines Mannes, mit jeweils fünf Emotionen [Freude, positive Überraschung, Wut, Angst, Traurigkeit] in 3 Intensitätsgraden [100%, 80%, 60%] 🡪 2 Sets mit je 15 Fotos  - 2 Proberunden vor der eigentlichen Testphase (Sortierung der Fotos nach Haarfarbe)  - Testphase direkt nach Proberunden (Sortierung der Fotos von Personen nach deren Gesichtsausdrücken zu einer Party in einem Haus, in dem nur glückliche/ positiv überraschte/ wütende/ ängstliche/ traurige Personen waren oder zu einer Party in einem Haus, in dem die Personen nicht glücklich/ positiv überrascht/ wütend/ ängstlich/ traurig waren)  - der Hälfte der Kinder wurden die Fotos der Frau und der anderen Hälfte die Fotos des Mannes gezeigt | **Keine Maskierung der Mundpartie:**  Experiment in der gleichen Art und Weise, aber es wurde das gesamte Gesicht ohne Maskierung der Mundpartie gezeigt | **Erkennen emotionaler Gesichtsausdrücke:** richtige Zuordnung zu folgenden 5 Emotionen:  1. Freude  2. positive Überraschung  3. Wut  4. Angst  5. Traurigkeit | **Durchschnittliche richtige Zuordnung der Emotionen** (5 x 3 x 2 Mixed Design ANOVA [5: Alter, 3: Intensitätsgrad der Emotion, 2: Testbedingung]):   - statistisch signifikante Effekte des Alters [*F*(4,190) = 9,74, *MSe* = 0,03, *p* < 0,01] und der Testbedingung [*F*(1,190) = 12,13, *MSe* = 0,04, *p* < 0,01], und signifikante Interaktion zwischen Alter und Testbedingung [*F*(4,190) = 15,06, *MSe* = 0,04, *p* < 0,01]   **Newman–Keuls paarweise Vergleich der Interaktion zwischen der Testbedingung** (Maske vs. keine Maske) **und Alter** (3–4 Jahre, 5–6 Jahre, 7–8 Jahre, 9–10 Jahre, Erwachsene):   - bei 3–4-Jährigen, 5–6-Jährigen und 7–8-Jährigen gab es keinen statistisch signifikanten Unterschied der richtigen Zuordnung der Emotionen zwischen den beiden Testbedingungen, aber die Zuordnung der Emotionen der unverdeckten Gesichter fiel 9–10-Jährigen und Erwachsenen leichter als die der maskierten Gesichter | **Finanzierung:** k.A.  **Interessenkonflikt:** k.A.  **Einverständnis einer Ethikkommission:** k.A.  + elterliche Zustimmung  + Auswahl der Fotographien ist validiert und wurde schon häufig in der neuropsychologischen Forschung und Entwicklungsforschung genutzt  + zufällige Reihenfolge der Fotos  + statistische Analyse (ANOVA)  - fehlende Angaben zur Rekrutierung und Response  - experimentelles Setting (Exposition wurde nicht unter Realbedingungen untersucht)  - zwar Stratifizierung der Ergebnisse nach Alter, aber keine Berücksichtigung des Confounders Geschlechts  - eindimensionales Outcome (Ja-Nein-Entscheidung) |
| Ruba, 2020 | **Design:** Experimentalstudie  **Land:** USA  **Zeitraum der Studie:** k.A.  **Anzahl der Untersuchungswellen:** *n* = 1 | **Beschreibung:** experimentelles Setting (Fotos wurden auf einem Laptop-Bildschirm gezeigt)  **Einschlusskriterien:** k.A.  **# eingeladen:** nicht zutreffend  **# Baseline:** k.A.  **Response:** k.A. | **Beschreibung:** schulpflichtige Kinder aus öffentlich geförderten Nachmittagsprogrammen (Alter: 7–13 Jahre)  **Einschlusskriterien:** k.A.  **# eingeladen:** k.A  **# Baseline:** *n* = 81  **Alter:**  *M =*9,86 Jahre (SD: 1,84, Spanne: 7,06–12,98)  **% weiblich:**  45,7 %  **Response:** k.A. | **Tragen einer OP-Maske:**  - Experiment, in dem Kinder aufgefordert wurden Ge-sichter be-stimmten Emotionen zuzuordnen (Fotos einer Frau und eines Mannes, mit jeweils 3 Emotionen[(Ärger, Angst, Traurigkeit] in 14 Auflösungsgraden, die sich in 3,3 Sekunden-Intervallen dynamisch höher auflösten  - Fotos wur-den der Matsumoto und Ekman Datenbank (1988) entnommen  - insgesamt mit Kontrollstimuli: 18 Stimuli à 14 Auflösungsgrade: für jedes Kind 252 Antworten gesammelt  - Fotos wurden in randomisierter Reihenfolge gezeigt  - Zuordnung jeden Bildes zu einer der folgenden Emotionen: “glücklich“, „traurig“, „wütend“, „überrascht“, „ängstlich“, „angewidert“ | **Kein Tragen einer OP-Maske:**  Experiment in der gleichen Art und Weise, aber Fotos mit:  a) unbedecktem Gesicht  b) Tragen einer Sonnenbrille | **Erkennen emotionaler Gesichtsausdrücke:** richtige Zuordnung zu folgenden 3 Emotionen:  1. Traurigkeit  2. Wut  3. Angst | **Durchschnittliche richtige Zuordnung aller Emotionen** (3 x 3 x 14 ANCOVA mit Testwiederholung [3: Emotion, 3: Testbedingung, 14: Auflösungsgrad]):   - statistisch signifikanter Effekt der Testbedingung (unbedecktes Gesicht, OP-Maske, Sonnenbrille) [*F*(2,154) =27,19, *p* < 0,001, *η_p_^2^*= 0,26], wobei die Zuordnung genauer war, wenn das Gesicht unbedeckt war [*M*= 0,34, *SD* = 0,47] im Vergleich zum Tragen von OP-Masken [*M*= 0,24, *SD*= 0,43)[ [*t*(80) = 6.57, *p*< 0,001] oder Sonnenbrille [*M*= 0,24, *SD*= 0,43)] [*t*(80) = 6,24, *p*< 0,001] - kein Unterschied zwischen den Testbedingungen des Tragens von OP-Masken und Sonnenbrille [*t*(80) = 0,20, *p* > 0,25] - statistisch signifikante Interaktion zwischen Testbedingung x Auflösungsgrad [*F*(18,1372) = 10,27, *p*< 0,001, *η_p_^2^*= 0,12] - 🡪 insgesamt eher geringer Einfluss der Gesichtsbedeckungen auf die Zuordnung, vor allem dann, wenn die Kinder mehr visuelle Informationen (durch höhere Auflösung) erhielten   **Durchschnittliche richtige Zuordnung der Emotion „Traurigkeit“:**   - statistisch signifikant ungenauere Zuordnung beim Tragen von OP-Masken [*M*= 0,28, *SD*= 0,45] im Vergleich zum unbedeckten Gesicht [*M*= 0,43, *SD*= 0,49] [*t*(80) = 4,60, *p*< 0,001] - kein statistisch signifikanter Unterschied zwischen dem Tragen von OP-Masken und Sonnenbrillen [*M* = 0,37, *SD*= 0,48] [*t*(80) = 2,47, *p* = 0,063] - auch kein statistisch signifikanter Unterschied zwischen dem Tragen von Sonnenbrille und unbedecktem Gesicht [*t*(80) = 0,91, *p*= 0,12] - alle Ergebnisse unabhängig vom Zufall (nach Bonferroni-Holm Korrektur)   **Durchschnittliche richtige Zuordnung der Emotion „Wut“:**   - statistisch signifikant ungenauere Zuordnung beim Tragen von OP-Masken [*M*= 0,27, *SD*= 0,44] im Vergleich zum unbedeckten Gesicht [*M*= 0,34, *SD*= 0,48] [*t*(80) = 2,72, *p*= 0,041] - kein statistisch signifikanter Unterschied zwischen dem Tragen von OP-Masken und Sonnenbrillen [*M* = 0,20, *SD*= 0,40] [*t*(80) = 2,16, *p* = 0,10] - auch statistisch signifikant ungenauere Zuordnung beim Tragen von Sonnenbrillen im Vergleich zu unbedecktem Gesicht [*t*(80) = 5,01, *p* < 0,001] - Ergebnisse für die Testbedingungen „Maskentragen“ und „unbedecktes Gesicht“ unabhängig vom Zufall, jedoch nicht für Testbedingung „Tragen einer Sonnenbrille“ (nach Bonferroni-Holm Korrektur)   **Durchschnittliche richtige Zuordnung der Emotion „Angst“:**   - statistisch signifikant ungenauere Zuordnung beim Tragen von OP-Masken [*M*= 0,18, *SD*= 0,38] im Vergleich zum unbedeckten Gesicht [*M*= 0,25, *SD*= 0,43] [*t*(80) = 2,91, *p*= 0,028] - kein statistisch signifikanter Unterschied zwischen dem Tragen von OP-Masken und Sonnenbrillen [*M* = 0,15, *SD*= 0,35] [*t*(80) = 1,09, *p* > 0,25] - auch statistisch signifikant ungenauere Zuordnung beim Tragen von Sonnenbrillen im Vergleich zu unbedecktem Gesicht [*t*(80) = 3,96, *p* < 0,001] - Ergebnisse nur für die Testbedingung „unbedecktes Gesicht“ unabhängig vom Zufall, jedoch nicht für Testbedingungen „Maskentragen und „Tragen einer Sonnenbrille“ (nach Bonferroni-Holm Korrektur) - Beim Tragen von Masken neigen Kinder dazu, ängstliche Gesichter der Emotion „Überraschung“ zuzuordnen | **Finanzierung:** öffentliche Förderung, Förder:innen hatten keinen Einfluss auf den Forschungsprozess  **Interessenkonflikt:** keiner  **Einverständnis einer Ethikkommission:** ja  + elterliche und kindliche Zustimmung  + zufällige Reihenfolge der Fotos  + dynamische Veränderung der Fotos von gering zu hoch aufgelösten Bildern entspricht eher alltäglichen Situationen der Gesichtserkennung als rein statisches Bild in hoher Auflösung  + Untersuchung des Einflusses der Confounder Alter und Geschlecht auf die Ergebnisse  + statistische Analyse (ANCOVA, inklusive Bonferroni-Holm Korrektur)  - fehlende detaillierte Angaben zur Rekrutierung und Response  - experimentelles Setting (Exposition wurde nicht unter Realbedingungen untersucht)  - eindimensionales Outcome (Ja-Nein-Entscheidung)  - keine Untersuchung eines zeitlichen Zusammen-hangs |
| Schwarz, 2021 | **Design:** Querschnittsstudie (Online-Register)  **Land:** Deutschland  **Zeitraum der Studie:** 20.–26.10.2020  **Anzahl der Untersuchungswellen:** *n* = 1  **Name der Kohorte:** Co-Ki-Register: Masken (Co-Ki: Coronakinderstudien) | **Beschreibung:** Ärzt:innen wurden gebeten selbst Eingaben zu machen und Eltern und Lehrer:innen auf das Register aufmerksam zu machen  **Einschlusskriterien:** nicht zutreffend  **# eingeladen:** nicht zutreffend  **# Baseline:** nicht zutreffend  **Response:** nicht zutreffend | **Beschreibung:** Kinder und Jugendliche (Alter: 0–17 Jahre)  **Einschlusskriterien:** k.A.  **# eingeladen:** nicht zutreffend  **# Baseline:** *n* = 25.930 (durch Einträge von *n* = 17.854 Eltern)  0–6-Jährige: 15,4% (*n* = 4.002)  7–12-Jährige: 55,6% (*n* = 14.407)  13–17-Jährige: 29,0% (*n* = 7.517)  **Alter:** k.A.  **% weiblich:**  47,2 %  **Response:** nicht zutreffend | **Tragen einer Mund-Nasen-Bedeckung (Maske) zur Prävention von COVID-19** | nicht zutreffend | **Symptome und Verhaltensauffälligkeiten, die durch das Tragen von Masken verursacht wurden:**  - quantitative Prävalenz  - qualitative Freitextangaben | **Durch das Maskentragen verursachte Symptome** (Prävalenz)   \|  \| Gesamt \| 0–6 Jahre \| 7–12 Jahre \| 13–17 Jahre \| \| --- \| --- \| --- \| --- \| --- \| \| Konzentrationsschwierigkeiten \| 49,5* \| 24,0 \| 50,8 \| 60,5 \| \| Beeinträchtigung beim Lernen \| 38,0* \| 15,5 \| 38,9 \| 48,2 \|   Unterschiede zwischen den Altersgruppen: *p > 0,0001 (Chi^2^-Test)  **Durch das Maskentragen verursachte Verhaltensveränderungen** (Prävalenz (%))   \|  \| Gesamt \| 0–6 Jahre \| 7–12 Jahre \| 13–17 Jahre \| \| --- \| --- \| --- \| --- \| --- \| \| Das Kind ist häufiger gereizt als sonst \| 60,4* \| 40,0 \| 62,1 \| 66,5 \| \| Das Kind ist weniger fröhlich \| 49,3* \| 36,9 \| 53,3 \| 47,6 \| \| Das Kind möchte nicht mehr zur Schule/ in den Kindergartengehen \| 44,0* \| 31,7 \| 48,9 \| 40,5 \| \| Das Kind ist unruhiger als sonst \| 29,2* \| 29,7 \| 33,2 \| 21,4 \| \| Das Kind schläft schlechter als sonst \| 31,1* \| 24,3 \| 33,2 \| 30,3 \| \| Keine weiteren Auffälligkeiten \| 27,4* \| 35,0 \| 26,6 \| 24,9 \| \| Das Kind hat neue Ängste entwickelt \| 25,3* \| 27,4 \| 27,8 \| 19,7 \| \| Das Kind schläft mehr als sonst \| 25,0* \| 12,3 \| 20,6 \| 39,1 \| \| Das Kind spielt weniger \| 15,5* \| 15,4 \| 18,9 \| 9,1 \| \| Das Kind hat einen größeren Bewegungsdrang als sonst \| 8,6* \| 9,7 \| 10,6 \| 4,2 \|   Unterschiede zwischen den Altersgruppen: *p > 0,0001 (Chi^2^-Test)  **Freitextangaben:**   - 112 Einträge zu Schulunlust bis hin zu Schulangst/Schulverweigerung - Ängste: allgemeine Zukunftsangst, Angst, selbst mit Maske zu ersticken, Angst, vor dem Tod von Angehörigen durch Corona, Angst vor Stigmatisierung durch das Tragen bzw. Nichttragen von Masken im sozialen Umfeld - Albträume und Angststörungen, welche sich auf maskierte Menschen beziehen, deren Mimik und Identität für die Kinder nicht erkennbar sind | **Finanzierung:** öffentliche Förderung  **Interessenkonflikt:** keiner  **Einverständnis einer Ethikkommission:** ja  + Untersuchung unter Realbedingungen der Pandemiesituation  + rasante Entwicklung des weltweit ersten Registers der Art  + hohe Teilnehmendenzahl  + Kombination aus Prävalenzangaben und qualitativen Freitexteintragungen  + Berücksichtigung des Confounders Alter in den Ergebnisanalysen  + Geschlechtsverteilung und Verteilung der Teilnehmenden nach Bundesländern sprechen für Repräsentativität der Zielgruppe  - Convenience Sampling (u. a. über soziale Medien, Ärzteschaft), hierbei gelangte der Link zur Studie laut Autor:innenangabe in Social-Media-Foren von Kritiker:innen der Coronamaßnahmen  - Zugänglichkeit des Online-Registers nicht für alle Bevölkerungsgruppen gleich zugänglich  - keine Vergleichsgruppe vorhanden  - subjektiver und eindimensionaler Outcome (Ja-Nein-Entscheidung)  - Verdachtsfälle von Nebenwirkungen, also medizinische Ereignisse, die im Rahmen der Anwendung von Masken durch die Eltern beobachtet wurden, aber nicht notwendigerweise mit der Maske im Zusammenhang stehen oder von ihr verursacht werden  - kausalattribuiertes Outcome (ggf. präferenzielle Teilnahme durch besonders schwer betroffene Kinder und Jugendliche)  - deskriptive Ergebnisauswertung basierend auf subjektiven Aussagen basierend auf Aussagen der Eltern, nicht der Kinder und Jugendlichen selbst  - keine Berücksichtigung des Confounders Geschlecht in den Ergebnisanalysen  - keine Untersuchung eines zeitlichen Zusammen-hangs |
| Singh, 2021 | **Design:** Experimentalstudie  **Land:** Singapur  **Zeitraum der Studie:** k.A.  **Anzahl der Untersuchungswellen:** *n* = 1 | **Einschlusskriterien:** k.A.  **# eingeladen:** nicht zutreffend  **# Baseline:** k.A.  **Response:** k.A. | **Beschreibung:** Kinder im Alter von 2 Jahren  **Einschlusskriterien:** Englisch als Muttersprache, Einsprachigkeit  **# eingeladen:** k.A  **# Baseline:** *n* = 24  **Alter:**  *M =*22,6 Monate (Spanne: 22 Monate und 1 Tag–23 Monate und 27 Tage)  **% weiblich:**  50 %  **Response:** k.A. | **Tragen einer Maske:**  a) OP-Maske  b) transparenter Visor  - im Experiment wurde den Kindern in der Mittel eines Computerbildschirms die Videoaufnahme einer Frau gezeigt, die folgendes sagte: „Kannst du der/die/das … sehen?“  - in die Lücke wurde jeweils 1 von 18 Zielwörtern eingesetzt  - gleichzeitig wurden den Kindern auf dem Bildschirm auf der einen Seite ein Bild eines der 18 Zielwörter und auf der anderen Seite ein Bild eines von 18 anderen Ablenkungswörter präsentiert  - anschließend wurde über die Augenbewegung der Kinder geprüft, ob ein Zielwort visuell richtig erkannt wurde oder nicht  - die Sprecherin trug entweder eine OP-Maske (in 6 Runden) oder einen Visor (in 6 Runden)  - insgesamt bestand das Experiment aus 18 Runden (mit 2 vorgeschalteten Proberunden) | **Kein Maskentragen:**  - Experiment in der gleichen Art und Weise, aber Wörter wurden ausgesprochen, ohne dass die Sprecherin eine Maske trug (in 6 Runden) | **Worterkennung** von 18 einsilbigen und bildlich vorstellbaren Zielwörtern, die frühzeitig von Englischsprachigen, monolingualen Kindern erworben werden  - Variable: Fixierungszeit des Zielwortes in der Phase vor und nach Vorspielen des Wortes | **Vergleich der Fixierungszeit vor und nach Vorspielen des Zielwortes je nach Exposition** (Confounder: Wortschatzumfang):   - **keine Maske:** t(23) = 3,01, p = 0,006, Cohen’s d = 0,93   🡪 statistisch signifikanter Anstieg der Fixierungszeit nach Abspielen des Zielwortes (spricht dafür, dass das Wort ohne Maske erkannt wurde)   - **OP-Maske:** t(23) = 3,51, p = 0,002, Cohen’s d = 0,86   🡪 statistisch signifikanter Anstieg der Fixierungszeit nach Abspielen des Zielwortes (spricht dafür, dass das Wort unter einer OP-Maske erkannt wurde)   - **Visor:** t(23) = 0,71, p = 0,49   🡪 keine statistisch signifikante Veränderung der Fixierungszeit vor und nach dem Abspielen des Zielwortes (spricht dafür, dass das Wort unter einem Visor nicht erkannt wurde)  **Zwischengruppenvergleich der Differenz der Fixierungszeit eines Zielwortes vor und nach dem Abspielen des Wortes** (mittels ANOVA mit wiederholten Messungen, Confounder: Wortschatzumfang):   - **Haupteffekt:** die Exposition des Maskentragens hat einen Einfluss auf den Anstieg der Fixierungszeit nach Abspielen des Zielwortes (F(2,44) = 3,62, p = 0,03, ηp^2^ =0,14) - **keine Maske versus OP-Maske:** F(1,22) = 0,57, p = 0,46 - **keine Maske versus Visor:** F(1,22) = 7,63, p = 0,01, ηp^2^ = 0,26 | **Finanzierung:** öffentliche Förderung  **Interessenkonflikt:** k.A.  **Einverständnis einer Ethikkommission:** k.A.  + Person, welche die Augenbewegungen auswerte, war gegenüber dem Untersuchungsziel, dem Expositionszustand (Maske versus keine Maske) verblindet und hatte keinen Zugang zum Sprachsignal  + die Reihenfolge der 18 Testrunden war für die einzelnen Kinder als auch zwischen den Kindern randomisiert  - die Rechts-/Linksverteilung der Ziel- und Ablenkungswörter war ausgeglichen  + die Paarung der Ziel- und Ablenkungswörter blieb aus visuellen Gründen konstant  + Outcome „Worterkennung“ wurde über automatisch ablaufende Augenbewegung ermittelt (objektives Messverfahren)  + Confounder Alter fand Berücksichtigung, da es sich um eine altershomogene Gruppe handelte  + der Umfang des Wortschatzes eines Kindes wurde als Confounder in den Analysen berücksichtigt  + statistische Analyse (ANOVA)  - fehlende detaillierte Angaben zur Rekrutierung und Response  - experimentelles Setting (Exposition wurde nicht unter Realbedingungen untersucht), damit können Faktoren wie z. B. Hintergrundgeräusche, die im realen Kontext auftreten, nicht dargestellt werden  - es wurden sehr einfache, einsilbige Wörter verwendet und bei den Ablenkungswörtern handelte es sich um phonetisch sehr unterschiedliche Wörter (einfacher Erkennungsgrad)  - keine Berücksichtigung des Confounders Geschlecht  - keine Untersuchung eines zeitlichen Zusammen-hangs |
| Stajduhar, 2021 | **Design:** Experi-mentalstudie  **Land:** Kanada  **Zeitraum der Studie:** November/Dezember 2020  **Anzahl der Untersuchungswellen:** *n* = 1 | **Beschreibung:** experimentelles Setting:  - Experiment wurde zu Hause am Computer durchgeführt (erreichbar über Link, den Eltern von den Forschenden zur Verfügung gestellt bekamen)  - Experiment konnte zu jeder Zeit durchgeführt werden  - bei Kindern unter 10 Jahren sollten die Eltern beim Lesen der Studienanweisungen helfen  **Einschlusskriterien:** k.A.  **# eingeladen:** nicht zutreffend  **# Baseline:** k.A.  **Response:** k.A. | **Beschreibung:** Schulkinder im Alter von 6–14 Jahren  **Einschlusskriterien:** k.A.  **# eingeladen:** k.A  **# Baseline:** *n* = 72  (Expositionsgruppe: *n* = 37, Vergleichsgruppe: *n* = 35)  **Alter:**  *M =*10,7 Jahre (SD: 2,3)  **% weiblich:**  45,8 %  **Response:** k.A. | **Tragen einer Maske:**  - im Experiment werden den Kindern Bilder mit Gesichtern von Jungen präsentiert, die eine Maske tragen  - 3 Phasen des Experiments (mit insgesamt 48 Runden):  1.) Erkennen von 4 männlichen Gesichtern von 3 Standpunkten aus  2.) Erweiterung um einen weiteren Standpunkt und Hinzunahme verschiedener Lichtverhältnisse  3.) wie 2.), aber zusätzlich mit visuellem Rauschen  - Ausrichtung der Gesichter:  a) aufrecht  b) umgedreht | **Kein Maskentragen:**  - Experiment in der gleichen Art und Weise, aber auf den Bildern tragen Jungen keine Maske | **Fähigkeit zur Gesichtserkennung:**  - Cambridge Face Memory Test – Kids (CFMT-K)  - Variable: Grad der Genauigkeit (Skala: 0–48) | **Deskriptiver Vergleich der Fähigkeit der Gesichtserkennung je nach Exposition:**   - für die Bilder der aufrechten Gesichter mit Maske lag ein circa 20 % niedrigerer CFMT-K-Score im Vergleich zu den Bildern der aufrechten Gesichter ohne Maske vor - spricht für eine eingeschränkte Fähigkeit zur Gesichtserkennung, sowohl im Hinblick auf die ganzheitliche Verarbeitung als auch die Verarbeitung bestimmter Merkmale - Maskeneffekt war für verschiedene Altersgruppen ähnlich groß (junge Kinder: -19,7 %, ältere Kinder: -22,4 %), d. h. sowohl junge als auch ältere Kinder erkannten Gesichter schlechter, wenn sie maskiert waren   **Vergleich der Fähigkeit der Gesichtserkennung je nach Exposition und Ausrichtung** (Maskentragen versus kein Maskentragen, mittels ANOVA mit wiederholten Messungen):   - statistisch signifikanter Effekt der Exposition „Maskentragen“: F(1,68) = 14,31, p < 0,001, ηp^2^ = 0,17 - zudem trat ein starker Effekt der Ausrichtung auf: F(1,68) = 55,31, p < 0,001, ηp^2^ = 0,44 - spricht für eine eingeschränktere Fähigkeit der Gesichtserkennung, wenn das Bild eines Gesichts umgekehrt gezeigt wird - diese Haupteffekte der Exposition und der Ausrichtung werden bekräftigt durch eine aufgezeigte gegenseitige Wechselwirkung zwischen Ausrichtung und Exposition: F(1,68) = 5,38, p < 0,05, ηp^2^ = 0,07 - Effekt der Ausrichtung trat sowohl bei maskierten (F(1,68) = 23,16, p < 0,001) als auch bei unmaskierten Gesichtern auf (F(1,68) = 31,74, p < 0,001), war aber für maskierte Gesichter geringer - spricht für eine Verschiebung der Gesichtswahrnehmung weg von einer ganzheitlichen hin zu einer mehr lokalen/analytischen Verarbeitung | **Finanzierung:** öffentliche Förderung  **Interessenkonflikt:** keiner  **Einverständnis einer Ethikkommission:** ja  + elterliche Zustimmung  + randomisierte Zuteilung zur Expositions- bzw. Vergleichsgruppe  + validierte Outcomeerhebung  + Berücksichtigung des Confounder Alter auf den Effekt des Maskentragens  + statistische Analyse (ANOVA)  - die Verteilung der Ausrichtung der Bilder (aufrecht oder umgedreht) war zwischen den Teilnehmenden ausgeglichen  + statistische Analyse (ANOVA)  - Rekrutierung über Schneeball-Sampling (keine genaueren Angaben vorhanden)  - keine Berücksichtigung des Confounders Geschlecht auf den Effekt des Maskentragens  - experimentelles Setting (Exposition wurde nicht unter Realbedingungen untersucht)  - nur Fotos von Jungen, nicht jedoch von Mädchen präsentiert  - keine Untersuchung eines zeitlichen Zusammen-hangs |

**Abkürzungen:** ANOVA Varianzanalyse, ANCOVA Kovarianzanalyse, F F-Wert, k.A. keine Angabe, KI Konfidenzintervall, M Mittelwert,MSe quatratischer Mittelwert des Fehlers, n Anzahl, OR Odds Ratio, p p-Wert, SD Standardabweichung, vs. versus

## 6. Datenextraktion der Reviews

| **Studie** | **Allgemein** | **Setting** | **Population** | **Exposition/ Intervention** | **Kontrolle/ Vergleich** | **Outcome** | **Ergebnisse** | **Kommentare** |
| --- | --- | --- | --- | --- | --- | --- | --- | --- |
| Erstautor:in, Jahr | Zeitraum des Reviews:  Anzahl der Studien (gesamt, themenrelevant):  Anzahl der Personen: | Beschreibung:  Einschlusskriterien:  Vergleichs-gruppe: | Beschreibung:  Einschlusskriterien:  Vergleichs-gruppe: | Beschreibung:  Einschlusskriterien: | Beschreibung:  Einschluss-kriterien: | Beschreibung:  Einschlusskriterien: | Beschreibung themenrelevanter Ergebnisse | Finanzierung *(keine, öffentliche, Non-Profit-, Industrie-Finanzierung, nicht berichtet)*  Interessenkonflikt *(keiner, vorhanden, nicht berichtet)* |
| Kisielinski, 2021 | **Zeitraum des Reviews:**  - qualitative Evaluation: Publikationen nicht älter als 20 Jahre  - quantitative Evaluation: 2004–31.10.2020  **Anzahl der Studien:**  gesamt: *n* = 109  themenrelevant: *n* = 1  **Anzahl der Personen:** k.A. | **Beschreibung:** k.A.  **Einschlusskriterien:** k.A. | **Beschreibung:** keine Einschränkung im eigentlichen Sinne, aber mit besonderem Fokus auf bestimmte Erkrankungsbilder, Patient:innen- bzw. Nutzer:innengruppen  **Einschlusskriterien:** k.A. | **Beschreibung:** Tragen verschiedener Arten von Masken, welche die Nasen-Mund-Region bedecken, also sowohl industriell gefertigte bzw. selbstgenähte Alltagsmasken als auch medizinische Masken wie OP-Masken oder FFP2-Masken  **Einschluss-kriterien:** k.A. | **Beschreibung:** k.A.  **Einschluss-kriterien:** k.A. | **Beschreibung:**  **-** nachteilige Auswirkungen oder Symptome durch das Tragen von Masken  - quantifizierbare nachteilige Auswirkungen:  1. statistisch signifikante Veränderung eines physiologischen Parameters in eine pathologische Richtung (p < 0,05)  2. statistisch signifikanter Symptomnachweis (p < 0,05)  3. Auftreten von Symptomen bei mindestens 50 % der Teilnehmenden einer Stichprobe (n ≥ 50 %)  **Einschlusskriterien:** k.A. | Die Übersichtsarbeit identifizierte nur eine Studie zur Beeinflussung der psychosozialen Entwicklung von Kindern und Jugendlichen durch die Pflicht zum Tragen von Gesichtsmasken im öffentlichen Raum zur Prävention von Infektionskrankheiten., die den Einschlusskriterien dieses systematischen Reviews genügt – und zwar die Querschnittsstudie von Schwarz et al., 2021. Die Autor:innen des Reviews schlussfolgern, basierend auf den in ihrem Review eingeschlossenen Publikationen, dass die durch das Maskentragen verminderte verbale und non-verbale Kommunikation und die damit einhergehende eingeschränkte soziale Interaktion für Kinder schwerwiegend sein kann. Weitere Forschung ist insbesondere für die vulnerable Gruppe von Kindern notwendig. | **Finanzierung:** keiner angegeben  **Interessenkonflikt:** k.A. |
| Sim, 2014 | **Zeitraum des Reviews:** k.A.  **Anzahl der Studien:**  gesamt: *n* = 51  themenrelevant: *n* = 0  **Anzahl der Personen:** k.A. | **Beschreibung:** Kommune und Krankenhaus-Setting  **Einschluss-kriterien:** k.A. | **Beschreibung:** k.A.  **Einschlusskriterien:** k.A. | **Beschreibung:** Verwendung einer Gesichtsmaske als primäre Präventionsmaßnahme zur Vorbeugung akuter Atemwegsinfektionen  **Einschluss-kriterien:** k.A. | **Beschreibung:** k.A.  **Einschluss-kriterien:** k.A. | **Beschreibung:**  1. Faktoren, welche die Verwendung von Gesichtsmasken beeinflussen (z. B. Alter, Geschlecht, Familienstand, Bildungsstand, Ethnizität, Wohnort)  2. Komponenten des Health-Belief-Modells, welche die Compliance des Tragens von Masken bestimmen: Empfindlichkeit, Schweregrad, Hindernisse, Vorteile, Handlungsanstöße  **Einschlusskriterien:** k.A. | Die Übersichtsarbeit identifizierte keine Studien mit Ergebnissen zur Beeinflussung der psychosozialen Entwicklung von Kindern und Jugendlichen durch die Pflicht zum Tragen von Gesichtsmasken im öffentlichen Raum zur Prävention von Infektionskrankheiten. | **Finanzierung:** k.A.  **Interessenkonflikt:** k.A. |

**Abkürzungen:** k.A. keine Angabe, n Anzahl

## 7. Methodendiskussion des systematischen Reviews

Nach unserem Kenntnisstand ist dies das erste Review, das sich mit der Beeinflussung der psychosozialen Entwicklung von Kindern und Jugendlichen durch das Tragen von Gesichtsmasken auseinandersetzt. Es wurde eine sehr sensitive und umfangreiche Literatursuche unter Nutzung grauer Literatur durchgeführt. Alle Prozessschritte wurden in Pilotphasen getestet und von mindestens zwei Reviewerinnen unter Zuhilfenahme von Entscheidungsleitfäden ausgeführt. Der Übereinstimmungsgrad in beiden Sichtungsphasen war nach Landis und Koch deutlich (Titel-Abstract-Sichtung) bzw. stark (Volltext-Sichtung).

A priori war der Einschluss von Experimentalstudien nicht geplant. Da diese jedoch wichtige themenrelevante Informationen liefern können, wurden sie im Verlauf des Reviewprozesses berücksichtigt. Die kritische Methodenbewertung sollte zunächst lediglich für epidemiologische Beobachtungsstudien vorgenommen werden, wurde aber auf die Studiendesigns „Mixed-Methods-Studien“, „Interventionsstudien“ und „Experimentalstudien“ erweitert, um die methodische Qualität aller eingeschlossenen Primärstudien miteinander vergleichen zu können.
